# Dynamic mortality predictions from serum albumin in dialysis patients using robust joint models with competing risks

**DOI:** 10.1038/s41598-025-21626-x

**Published:** 2025-10-08

**Authors:** Ivan Damgov, Meinhard Kieser, Peter Rutherford, Simon J. Davies, Muh Geot Wong, Carol Pollock, David W. Johnson, Claus Peter Schmitt

**Affiliations:** 1 Division of Pediatric Nephrology, Center for Pediatric and Adolescent Medicine, University of Heidelberg, Im Neuenheimer Feld 430, 69120 Heidelberg, Germany; 2 Institute of Medical Biometry, University of Heidelberg, Heidelberg, Germany; 3 Baxter Healthcare Corporation, Zurich, Switzerland; 4 Faculty of Medicine and Health Sciences, Keele University, Stoke-On-Trent, UK; 5 Department of Renal Medicine, Concord Repatriation General Hospital, University of Sydney, Concord, Australia; 6 Sydney Medical School, Kolling Institute, University of Sydney, Royal North Shore Hospital, St Leonards, New South Wales Australia; 7 Australasian Kidney Trials Network, University of Queensland, Brisbane, Australia; 8 Department of Kidney and Transplant Services, Princess Alexandra Hospital, Brisbane, Australia

**Keywords:** Albumin, All-cause mortality, Dynamic predictions, Haemodialysis, Peritoneal dialysis, End-stage renal disease, Software

## Abstract

This study introduces advanced Joint Models (JM) to enhance mortality prediction in dialysis patients, addressing the inadequacies of existing models which lack clinical precision. We analyzed the relationship between albumin trajectories and mortality in 314 peritoneal dialysis patients over eight years, using training and validation datasets. We developed 12 JM, incorporating seven baseline risk factors; 10 models addressed individual and within-patient trajectory outliers, and six models included the competing events of hemodialysis transfer and kidney transplantation. These were also applied to a hemodialysis cohort of 315 patients. Findings showed a consistent albumin hazard ratio for death across all JM, ranging from 1.22 to 1.28, confirmed by two simulation studies. JM software optimizations achieved 3- to sixfold faster processing and 4.1- to 12.9-fold higher efficiency than conventional software. Longer follow-up improved all JM accuracies, and models with competing risks outperformed those considering only outliers. Across forecasting periods up to five years, all JM consistently surpassed benchmark Cox models, demonstrating significantly higher Area Under Curve (AUC) scores in both peritoneal and hemodialysis groups. In conclusion, our JM combine established risk factors with dynamic individual albumin profiles, integrating outliers and competing risks, substantially enhancing mortality predictions for dialysis patients.

**Supplementary Information:**

The online version contains supplementary material available at 10.1038/s41598-025-21626-x.

## Introduction

While peritoneal dialysis (PD) and hemodialysis (HD) control the life-threatening complications of kidney failure, both are associated with major morbidity and a 10–20-fold increased death risk compared to the age-matched general population^1^. Multiple risk factors for adverse outcomes have been identified, and various death prediction models have been established, yet none are sufficiently accurate for routine clinical use^2,3^. Timely identification of high-risk dialysis patients is crucial for individual risk factor-based shared decision-making. Potential measures include intensified dialysis regimen^4^, modality switch, and (re-)evaluation options for timely kidney transplantation. Accurate population-based risk predictions are also essential for enhancing clinical trial recruitment and informing future health economics.

All-cause mortality provides a comprehensive view of dialysis patient outcomes, encompassing all death causes related to the treatment. Research has demonstrated a negative association between baseline serum albumin levels^5–8^, changes over time and dialysis patient mortality. Some studies have used single summary measures^5,9–13^ others applied albumin as a time-varying covariate in extended Cox model^14–17^. However, these approaches may ignore dynamic serum albumin changes, potentially introducing bias mortality risk estimation^18^. Furthermore, time-varying Cox models assume that longitudinal measurements are free from variability due to technical errors, biological variability, and trends^19^.

Joint models (JM) have been developed to improve prediction by combining a longitudinal sub-model for biomarker evolution, accounting for both intra- and inter-individual variability, with a time-to-event process describing clinical outcome risks. Properly specified JM have shown enhanced efficiency and reduced bias compared to Cox models^19^. They have demonstrated great potential in other fields of medicine for risk factor identification and dynamic predictions, but their application in the field of dialysis is still limited^19^. This may be attributed to the greater computational challenges in JM estimation in large datasets^20^ and model assumptions limiting clinical data applicability^21^. Traditional JM assume normally distributed random effects and error terms in longitudinal biomarker trajectories. Yet, real-life data often includes outliers at the patient level (*b*-outliers) and within patient-specific trends (*e*-outliers)^21^. Furthermore, death on dialysis is only observed if it occurs before other competing events, such as dialysis modality switch or kidney transplantation (KTx). Since treating competing events as independent censoring can overestimate death probability^22^, JM should account for competing risks^23^.

In this study, we developed and investigated novel JM models, incorporating baseline risk profile, complex outlier structures in albumin trajectories, and competing risks. These inform on sensitivity of longitudinal albumin hazard ratio estimates and demonstrate superior prediction accuracy, and updated dynamically with follow-up albumin values. Our advanced software solution achieved several-fold increase in calculation speed and efficiency over proprietary packages and overcomes computational challenges currently limiting broader JM application.

## Materials and methods

### Study design and clinical trial data collection

This study is a post hoc analysis of the IDEAL trial (Australian and New Zealand Clinical Trials Registry number 12609000266268), approved by the trial investigators and conducted in accordance with the Declaration of Helsinki and local regulations. The IDEAL trial’s study design, methodology, and findings have been previously published^24,25^. The trial randomized adult patients with progressive CKD between July 2000 and November 2008 to early dialysis initiation (GFR 10.0–14.0 mL/min) or late start (GFR 5.0–7.0 mL/min). All patients gave written informed consent. Patients were monitored for 8 years, with three-monthly assessments during the first 3 years and six-monthly follow-ups thereafter, tracking parameters such as 24-h urine, biochemistry, medication, clinical outcomes, and dialysis regimen. Each patient was followed from dialysis onset until death, transfer to HD, transplantation, or study dropout. Throughout IDEAL all sites quantified serum albumin with the bromocresol-green colorimetric assay mandated by the protocol, so cross-laboratory normalization to an immunoturbidimetric reference range was not required.

Dialysis patients were included if baseline parameters were documented within ± 90 days of starting dialysis and at least one post-dialysis albumin value was available. A total of 314 PD patients were analysed, split into a training set (N = 236) and a testing set (N = 78). The training set was used for model development, and the testing set evaluated predictive performance. Competing events (switch to HD and KTx) were initially treated as right-censored and later modelled with a cause-specific hazard. The longitudinal outcome was serum albumin levels (g/L) at follow-up visits, and the association between albumin and mortality was explored. Dialysis-specific deaths were included if they occurred within three months of modality switch. The same analyses were applied to 315 HD patients from the IDEAL trial, split into training (N = 236) and testing (N = 79) datasets. Study flowcharts are shown in Figures S1,2.

### Statistical analyses and JM building workflow

The standard JM (‘*nor-nor*’) consists of two parts linked through shared random effects: (i) linear mixed effects sub-model for longitudinal albumin, assuming zero-mean normal distributions for random effects and residual error, and (ii) survival sub-model for time-to-event data defined by a hazard function. We investigated five additional robust JM, which utilized heavy-tailed Student’s *t-*distribution for random effects and/or residual error allowing for the presence of *b-* and/or *e-*outliers. Weibull proportional hazard (PH) model was used to model the baseline hazard of all-cause mortality. Key model assumptions are given in Table 1 (detailed specification in Suppl. Sections 1.3.1 and 1.3.2).

**Table 1:** Joint model nomenclature and constituent parts.

Model Name	Longitudinal Sub-model	Time-to-Event Sub-Model
nor-nor	Random effects: multivariate normal; Error: normal	Survival only (competing risks ignored)
t-t-mod1	${\mathbf{B}}_{\mathbf{i}}$ and ${\mathbf{Z}}_{\mathbf{i}}$ are jointly multivariate *t* with the single degrees-of-freedom parameter,$\gamma$	Survival only (competing risks ignored)
t-t-mod2	${\mathbf{B}}_{\mathbf{i}}$ and ${\mathbf{Z}}_{\mathbf{i}}$ are multivariate *t* with separate degrees of freedom parameters, $\phi$ and $\delta$, respectively	Survival only (competing risks ignored)
nor-t-mod2	Random effects: multivariate normal; Error: as per ‘*t-t-mod2*’	Survival only (competing risks ignored)
t-t-mod3	${\mathbf{B}}_{\mathbf{i}}$ and ${\mathbf{Z}}_{\mathbf{i}}$ are multivariate *t* with separate degrees of freedom parameters, $\phi$ and $\delta$, respectively, whereby dependence between ${Z}_{ij}$ and ${Z}_{i{j}{\prime}}$ has been removed	Survival only (competing risks ignored)
nor-t-mod3	Random effects: multivariate normal; Error: as per ‘*t-t-mod3*’	Survival only (competing risks ignored)
nor-nor-cr	Same as ‘*nor-nor*’	Weibull cause-specific hazard for death, transfer to HD and KTx
t-t-mod1-cr	Same as ‘*t-t-mod1’*	Weibull cause-specific hazard for death, transfer to HD and KTx
t-t-mod2-cr	Same as *‘t-t-mod2’*	Weibull cause-specific hazard for death, transfer to HD and KTx
nor-t-mod2-cr	Same as ‘*nor-t-mod2*’	Weibull cause-specific hazard for death, transfer to HD and KTx
t-t-mod3-cr	Same as ‘*t-t-mod3*’	Weibull cause-specific hazard for death, transfer to HD and KTx
nor-t-mod3-cr	Same as ‘*nor-t-mod3’*	Weibull cause-specific hazard for death, transfer to HD and KTx

To adjust for competing risks, six JM were developed with a cause-specific Weibull PH hazards model, where events of all-cause death, transfer to HD, and KTx each have their own baseline risk factors (Suppl. 1.3.4). A detailed JM building workflow is featured in the Supplementary file (covariate selection in 1.2, model diagnostics and selection in 1.4). Comorbidities were evaluated using the Stoke–Davies comorbidity score^26^. The widely applicable information criterion (WAIC)^27^ was used to assess best fit to the data (the smaller, the better), whereby differences of at least 5 points were considered of importance^28^.

The developed JM were compared to two Cox models fitted to the IDEAL PD training dataset: one using baseline albumin (‘*Cox 1*’) and an extended Cox model with albumin as a time-varying covariate (‘Cox 2’). Both Cox models shared the same baseline risk factors as the JM survival sub-model. An innovative program for JM estimation and prediction was developed using the Hamiltonian Monte Carlo engine Stan^29^ under Bayesian inference (Suppl. Section 2). Speed and sampling were compared with the **robjm** package^20,21^ for 6 JM without competing risks (Suppl. Section 2). The two Cox survival models were fitted with the *coxph*() function in **R** under frequentist estimation. Two simulation studies were performed to validate findings for the effect of longitudinal albumin on the risk of death in the presence of outliers and competing risks (Suppl. Section 4).

The landmark approach^20,30^ was used to generate dynamic individual predictions of all-cause mortality. For new PD/HD patients in the testing datasets with longitudinal observations until a landmark time ‘*s*’, predictions were made for their future albumin value and survival probability at time ‘*s* + *u*’, where ‘*u’* denotes horizon time. Due to the modest size of the prediction datasets, three landmark times were considered: 1 year, 1.5 years and 2 years after starting dialysis, with prediction horizons set at 6 months and 1 year. To evaluate long-term prediction accuracy, additional forecasts at 6-month intervals up to 5 years on dialysis were calculated. Time-dependent Area Under the Curve (AUC) and Brier score (BS) were used to estimate and compare prediction accuracy for survival of the proposed JM and the two Cox models. AUC assessed the model’s ability to discriminate between patients with and without the predicted event, while BS evaluated both discrimination and calibration aspects of the prediction^20,30,31^. Both were calculated using the **timeROC** package in **R**^32^. Additionally, calibration of predictions was assessed using calibration plots, comparing deciles of predicted mortality rates in the testing dataset to observed survival determined using the Kaplan–Meier method^33^. A third simulation study was conducted to validate dynamic predictions findings.

## Results

### JM development (PD training dataset)

**Fig. 1:**
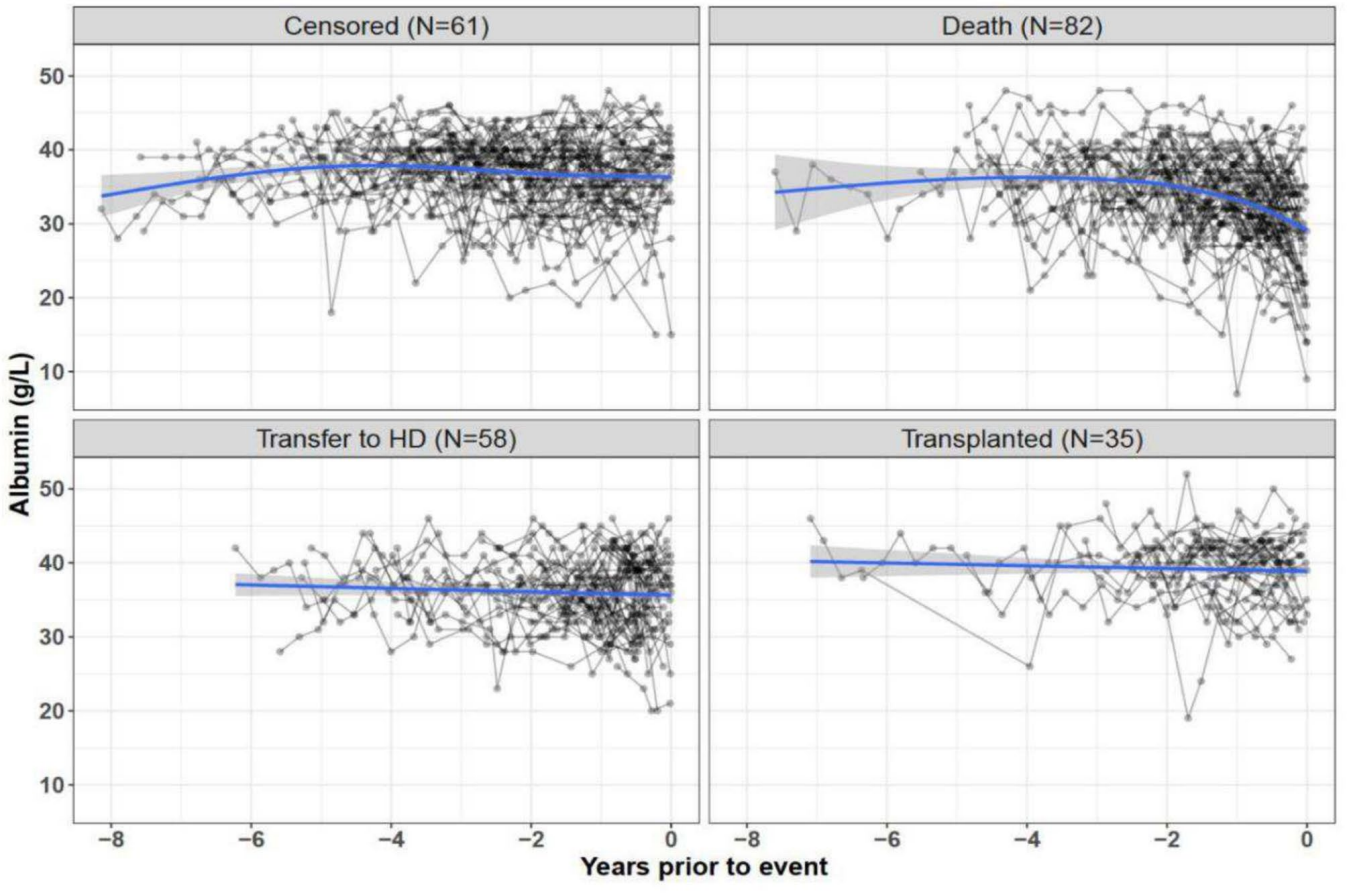
Albumin measurements in patients from the IDEAL trial PD training dataset (N = 236) in reverse time according to event type. The blue lines indicate the LOWESS smoothing curve.

Table S3 presents the baseline characteristics of PD patients in the IDEAL PD training dataset categorized by event status. Stepwise selection (Fig. S3) identified 7 baseline parameters explaining the risk of all-cause mortality: age, gender, Stoke/Davies score, initial dialysis dose, creatinine, urea and serum cholesterol, included in all JM and the two Cox-models. For JM with competing risks, age, BMI, smoking status and cumulative number of peritonitis events were selected as risk factors for the transfer to HD sub-model, while age, ethnicity, Stoke/Davies score, and smoking status were included for the KTx sub-model. Primary causes of death were CVD (50.0% in the PD training and 46.9% in the testing dataset) and infectious diseases (Table S4). Mean serum albumin concentrations declined in the year before death, but remained stable in censored patients and those with a competing event (Fig. 1).

Six link functions modelling the association between albumin trajectory and all-cause mortality were tested in the training dataset The ‘current value’ link function showed best fit (Suppl. 1.3.1, 3.2 and 3.9) and was applied for all JM. The standard JM model (‘*nor-nor*’) with normal distribution assumptions for random effects and measurement error was first fitted to the training dataset. For the survival sub-model, Weibull PH was appropriate to determine the baseline hazard of all-cause mortality (Suppl. 3.3). For the albumin trajectory sub-model, Residuals vs Fitted plot indicated linear time trend for albumin (Fig. S6A), but the quantile–quantile (Q-Q) plot of the standardized conditional residuals demonstrated considerable departures from the normal distribution assumptions (Fig. S6B), characteristic for heavy-tailedness of the residuals. This suggested the presence of outliers in the albumin values, due to outlying individuals or outlying observations within individuals, or a mixture of both.

Ten robust JM were developed to account for these outlier structures (Table 1; Suppl. 1.3.2). The standard JM and five of the robust JM treated competing risks as censoring events. Estimation with the training dataset yielded best fit for model ‘*t-t-mod3*’, the most complex one, and worst fit for the standard JM (Table 2). Table S7 provides posterior summaries of the JM parameters fitted to the IDEAL PD training dataset, showing the impact of clinical and biochemical baseline variables on serum albumin concentrations along with covariance structure, degrees of freedom parameters, and the survival sub-model hazard ratios (HR) of the six JM. Model ‘*t-t-mod3*’ assumed heavy-tailed *t*-distribution for both random effects and error structure, with low estimates of the degrees of freedom parameters $\phi$ and $\delta$ indicating both *b-* and *e-*outliers in albumin trajectory, identified by the JM (Suppl. 3.5). Violation of normality assumption due to outliers mostly impacted the rate of decline in albumin and residual standard error, $\sigma$, with the five robust JM, while the HR of the survival-sub model remained largely unaffected by the modelled albumin outlier structure (Table S7, Fig. 3A). The advanced JM software achieved 3- to sixfold higher speed and 4.1- to 12.9-fold higher sampling efficiency than the recent **robjm** package^20,21^, see Suppl. Section 2.

**Table 2:** Comparison of standard and robust JM fits of IDEAL PD training dataset with and without adjustment for competing risks.

Models without competing risks				Models with competing risks			
Model*	**WAIC**	**Rank**	**Estimation time (min)**	Model**	**WAIC**	**Rank**	**Estimation time (min)**
nor-nor	12,518.14	6	5.8	**nor-nor-cr**	13,112.24	6	19.6
t-t-mod1	12,168.90	3	6.8	**t-t-mod1-cr**	12,760.50	3	20.5
t-t-mod2	12,175.97	4	12	**t-t-mod2-cr**	12,760.96	4	38.1
nor-t-mod2	12,193.00	5	8.7	**nor-t-mod2-cr**	12,785.48	5	23.8
t-t-mod3	11,962.64	1	15	**t-t-mod3-cr**	12,542.83	1	46.9
nor-t-mod3	11,968.14	2	12.2	**nor-t-mod3-cr**	12,556.41	2	38.7

**Fig. 2:**
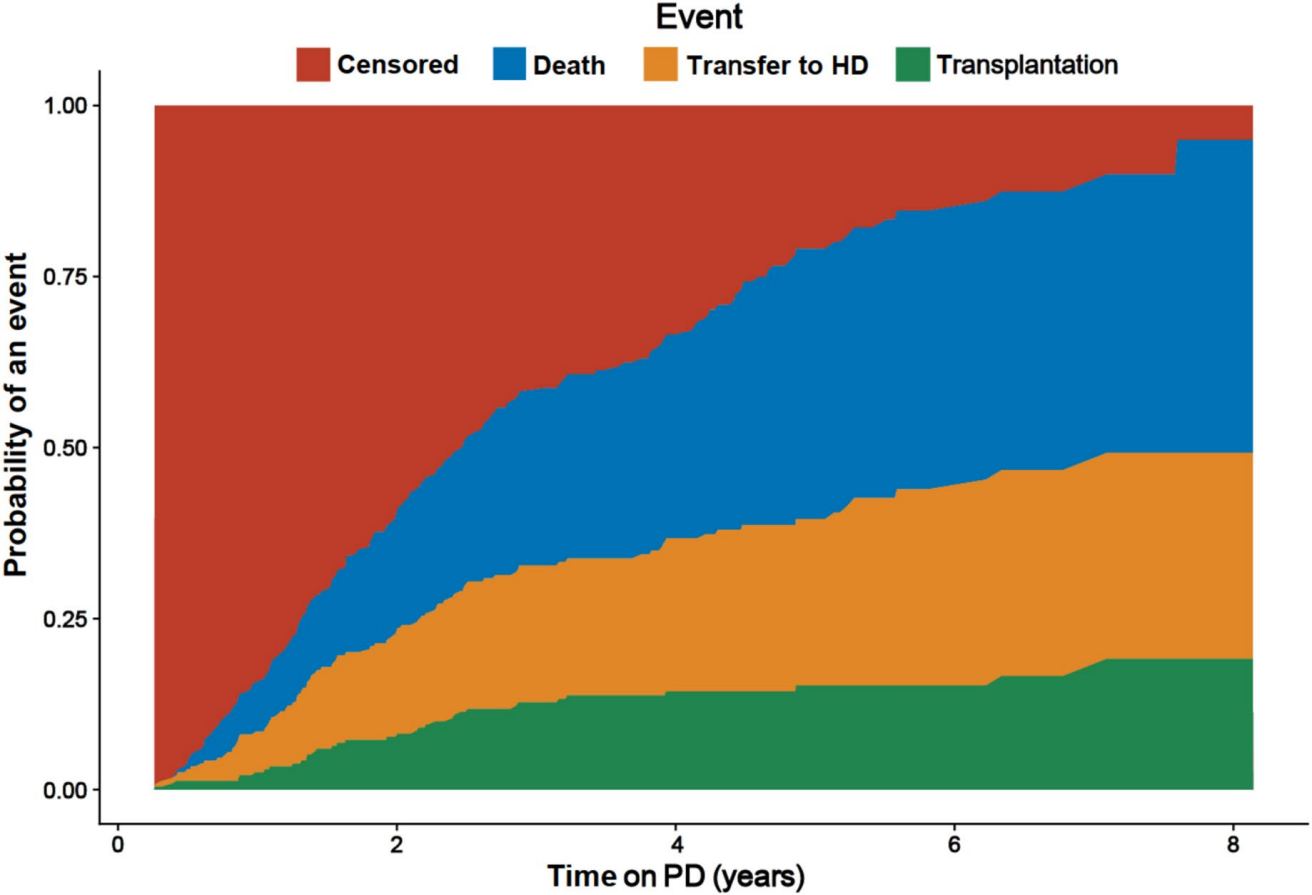
Cumulative incidence plot of competing events with the IDEAL PD training dataset. HD: hemodialysis.

**Fig. 3:**
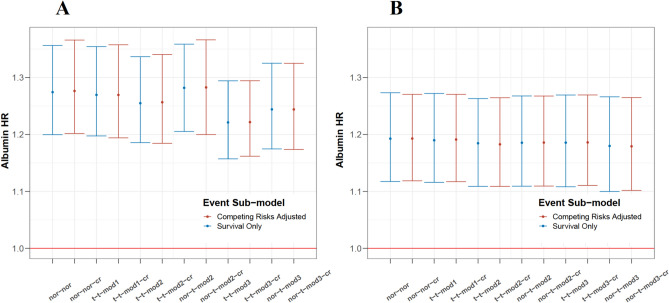
Hazard ratio (HR) per 1 g/L decrease in albumin along with 95% credible intervals estimated from standard and robust JM with and without adjustment for competing risks fitted to A: IDEAL PD training dataset; B: IDEAL HD training dataset.

Cumulative incidences of competing events (death, switch to HD and transplantation) are shown in Fig. 2. The shapes of the cumulative incidence curves for competing events were similar to the death curve, thereby justifying the use of a cause-specific Weibull PH hazard model. Six JM were developed accounting for competing risks (Table 1). Among these, ‘*t-t-mod3-cr*’ model achieved the best fit (Table 2). It shared the same robust mixed model effects structure as model ‘*t-t-mod3*’, and reconfirmed the presence of both *b-* and *e-*outliers in albumin trajectory. Adjustment for competing risks resulted in minor changes in the parameter estimates for the covariates in the albumin sub-model and in the survival sub-model HR estimates, as compared to the six models ignoring competing risks (Tables S7, S8). Across all 12 JM, albumin HR for death was very robust, with a 1 g/L decline in serum albumin increasing the risk of all-cause mortality by 1.22- to 1.28-fold (Fig. 3A). Because the survival sub-model employs a current-value association, the hazard ratio reflects the instantaneous effect of a lower albumin concentration and is independent of the speed at which that concentration was reached. No association was found between longitudinal albumin and the risk of transfer to HD and KTx in any of the six JM considering competing risks (Table S8). Parameters from all JM demonstrated excellent estimation properties (Suppl. 3.4) and were stable under alternative priors (Suppl. 1.3.3). For further comparison, HR results from Cox PH models for all-cause mortality, ‘*Cox-1*’ and ‘*Cox-2*’, considered for dynamic predictions, are also provided (Suppl. 3.6). Two simulation studies in our supplementary materials confirm that the findings generalize beyond the observed data. In 200 IDEAL-like cohorts without competing risks (Supp. 4.1) and in a second set with two competing events added (Supp. 4.2) both consisting of 47,200 patient trajectories, the JM recovered the true parameters with negligible bias.

### Dynamic predictions with JM (PD testing dataset)

**Fig. 4:**
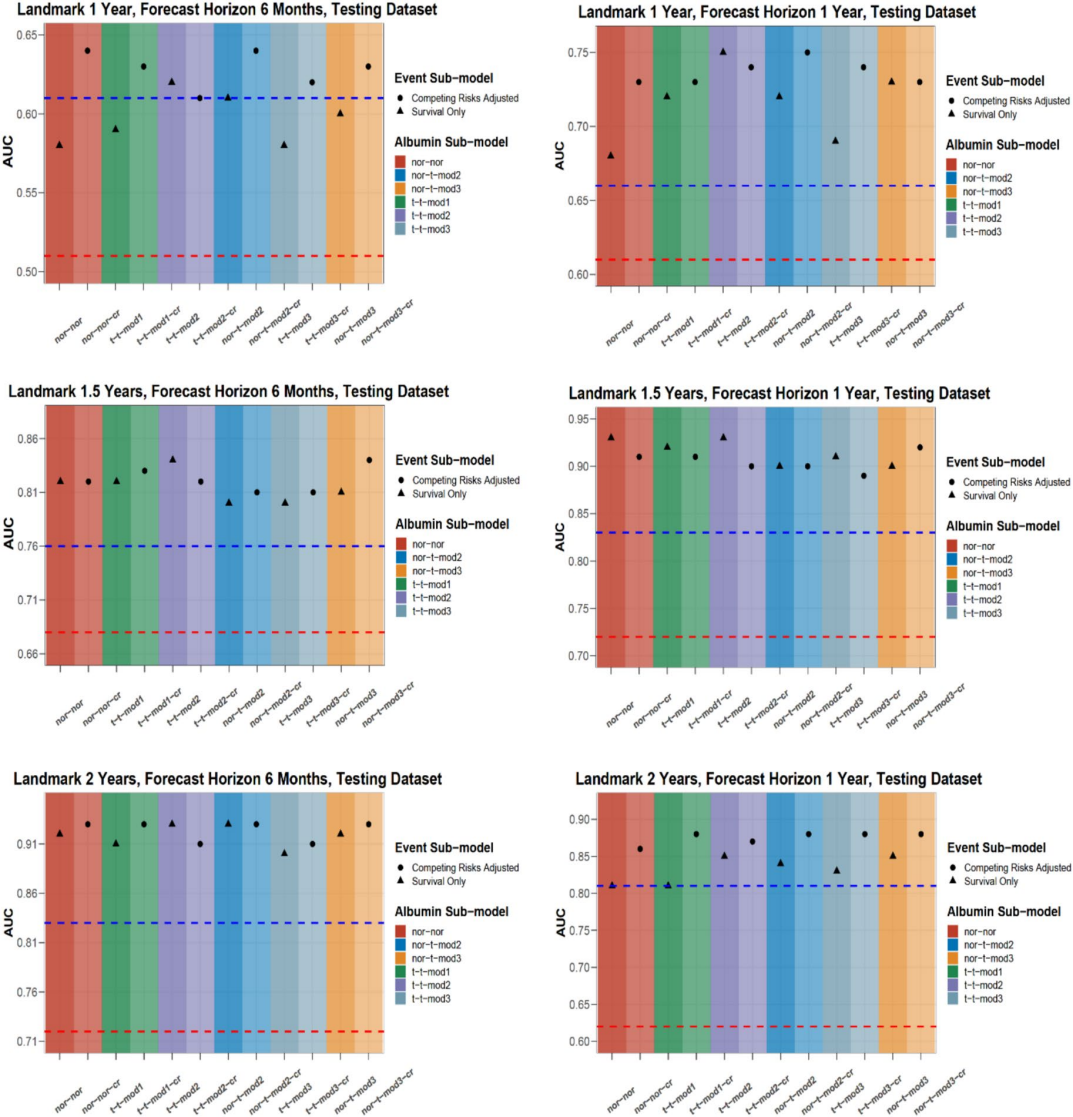
Area under the curve (AUC) of dynamic predictions for all landmark times and two prediction horizons with IDEAL trial PD testing dataset. Higher AUC values indicate better accuracy in terms of greater discrimination of the survival prognosis. Left column displays results for forecast horizons of 6 months, right column displays results for forecast horizons of 1 year. JM which ignore competing risks (black triangles) are placed right next to the JM extension which accounts for competing risks (black circles) grouped according to the longitudinal sub-model type (e.g., ‘nor-nor-cr’ follows ‘nor-nor’). Red dashed horizontal line indicates result from Cox PH model with baseline albumin value (‘Cox-1’ in text) and blue dashed line indicates result from extended Cox PH model with albumin as a time-varying covariate (‘Cox-2’ in text).

Table S12 shows frequencies of subjects in the PD testing dataset who were at risk, died, had a competing event, or were censored within forecast horizons of 6 months and 1 year at landmark times of 1, 1.5, and 2 years after PD start. Figures 4 and S11 display estimated AUC and BS for these landmark times and forecast horizons. The 12 proposed JM demonstrated acceptable to outstanding discrimination^3^, which increased substantially with longer landmark times (AUC range at 1, 1.5, and 2 years: 0.68–0.75, 0.89–0.93, and 0.81–0.88, respectively). Thus, longer follow-up allowed JM to better estimate the individual underlying albumin trajectory, resulting in more accurate survival predictions (Table 3, Figs. 4 and S11). Robust JM ignoring competing risks offered a slight AUC increase over the simpler standard JM model (‘*nor-nor*’) in the 1-year time horizon. Accounting for competing risks increased the AUC values across all albumin sub-models. The JM outperformed both Cox models, with an increasing advantage in AUC at later landmark times in both 6-month and 1-year forecast horizons, and in long-term horizons up to 5 years on PD (Table 4, Table S13).

**Table 3:** Comparison of standard and robust JM fits of IDEAL HD training dataset with and without adjustment for competing risks.

Models without competing risks				Models with competing risks			
Model*	**WAIC**	**Rank**	**Estimation time (min)**	Model**	**WAIC**	**Rank**	**Estimation time (min)**
nor-nor	15,129.87	6	7	**nor-nor-cr**	15,457.22	6	25.1
t-t-mod1	14,634.65	3	8.8	**t-t-mod1-cr**	14,957.66	3	43.3
t-t-mod2	14,665.48	5	21.8	**t-t-mod2-cr**	14,993.02	5	49.1
nor-t-mod2	14,653.97	4	8.1	**nor-t-mod2-cr**	14,983.12	4	33.7
t-t-mod3	14,372.31	1	24.7	**t-t-mod3-cr**	14,689.02	1	46.6
nor-t-mod3	14,385.56	2	10.6	**nor-t-mod3-cr**	14,699.89	2	27

**Table 4:** Area under the curve (AUC) of dynamic predictions for three landmark times and various prediction horizons with IDEAL PD testing dataset*.*

Landmark time (Year)	Forecast Time (Year)	Cox-1	Cox-2	nor	nor-cr	t-t-mod1	t-t-mod1-cr	t-t-mod2	t-t-mod2-cr	nor-t-mod2	nor-t-mod2-cr	t-t-mod3	t-t-mod3-cr	nor-t-mod3	nor-t-mod3-cr
1	1.5	0.51	0.61	0.58	0.64	0.59	0.63	0.62	0.61	0.61	0.64	0.58	0.62	0.6	0.63
	2	0.61	0.66	0.68	0.73	0.72	0.73	0.75	0.74	0.72	0.75	0.69	0.74	0.73	0.73
	2.5	0.66	0.77	0.8	0.84	0.83	0.83	0.84	0.83	0.83	0.84	0.83	0.83	0.84	0.83
	3	0.62	0.74	0.81	0.85	0.8	0.84	0.82	0.84	0.83	0.84	0.82	0.82	0.82	0.82
	3.5	0.61	0.75	0.83	0.86	0.83	0.85	0.84	0.84	0.86	0.84	0.85	0.83	0.84	0.82
	4	0.49	0.63	0.73	0.76	0.72	0.76	0.69	0.75	0.72	0.74	0.72	0.73	0.72	0.73
	4.5	0.56	0.65	0.79	0.78	0.78	0.78	0.77	0.78	0.79	0.77	0.77	0.75	0.75	0.76
	5	0.48	0.68	0.82	0.76	0.86	0.76	0.85	0.77	0.84	0.76	0.81	0.74	0.84	0.73
1.5	2	0.68	0.76	0.82	0.82	0.82	0.83	0.84	0.82	0.8	0.81	0.8	0.81	0.81	0.84
	2.5	0.72	0.83	0.93	0.91	0.92	0.91	0.93	0.9	0.9	0.9	0.91	0.89	0.9	0.92
	3	0.65	0.76	0.85	0.86	0.85	0.87	0.86	0.86	0.83	0.86	0.86	0.85	0.84	0.86
	3.5	0.64	0.76	0.88	0.86	0.85	0.87	0.87	0.86	0.86	0.86	0.87	0.85	0.85	0.86
	4	0.5	0.62	0.72	0.72	0.71	0.73	0.69	0.72	0.7	0.72	0.7	0.72	0.67	0.73
	4.5	0.57	0.65	0.75	0.73	0.71	0.73	0.7	0.71	0.72	0.72	0.7	0.72	0.71	0.72
	5	0.49	0.6	0.78	0.7	0.74	0.69	0.7	0.68	0.72	0.69	0.72	0.68	0.72	0.69
2	2.5	0.72	0.83	0.92	0.93	0.91	0.93	0.93	0.91	0.93	0.93	0.9	0.91	0.92	0.93
	3	0.62	0.81	0.81	0.86	0.81	0.88	0.85	0.87	0.84	0.88	0.83	0.88	0.85	0.88
	3.5	0.61	0.83	0.82	0.83	0.82	0.85	0.86	0.83	0.86	0.83	0.82	0.84	0.87	0.87
	4	0.44	0.6	0.76	0.74	0.73	0.74	0.68	0.71	0.73	0.71	0.63	0.64	0.67	0.71
	4.5	0.54	0.62	0.78	0.75	0.75	0.74	0.7	0.72	0.74	0.72	0.63	0.67	0.7	0.72
	5	0.45	0.59	0.76	0.68	0.75	0.67	0.71	0.65	0.74	0.66	0.63	0.63	0.72	0.66

**Fig. 5:**
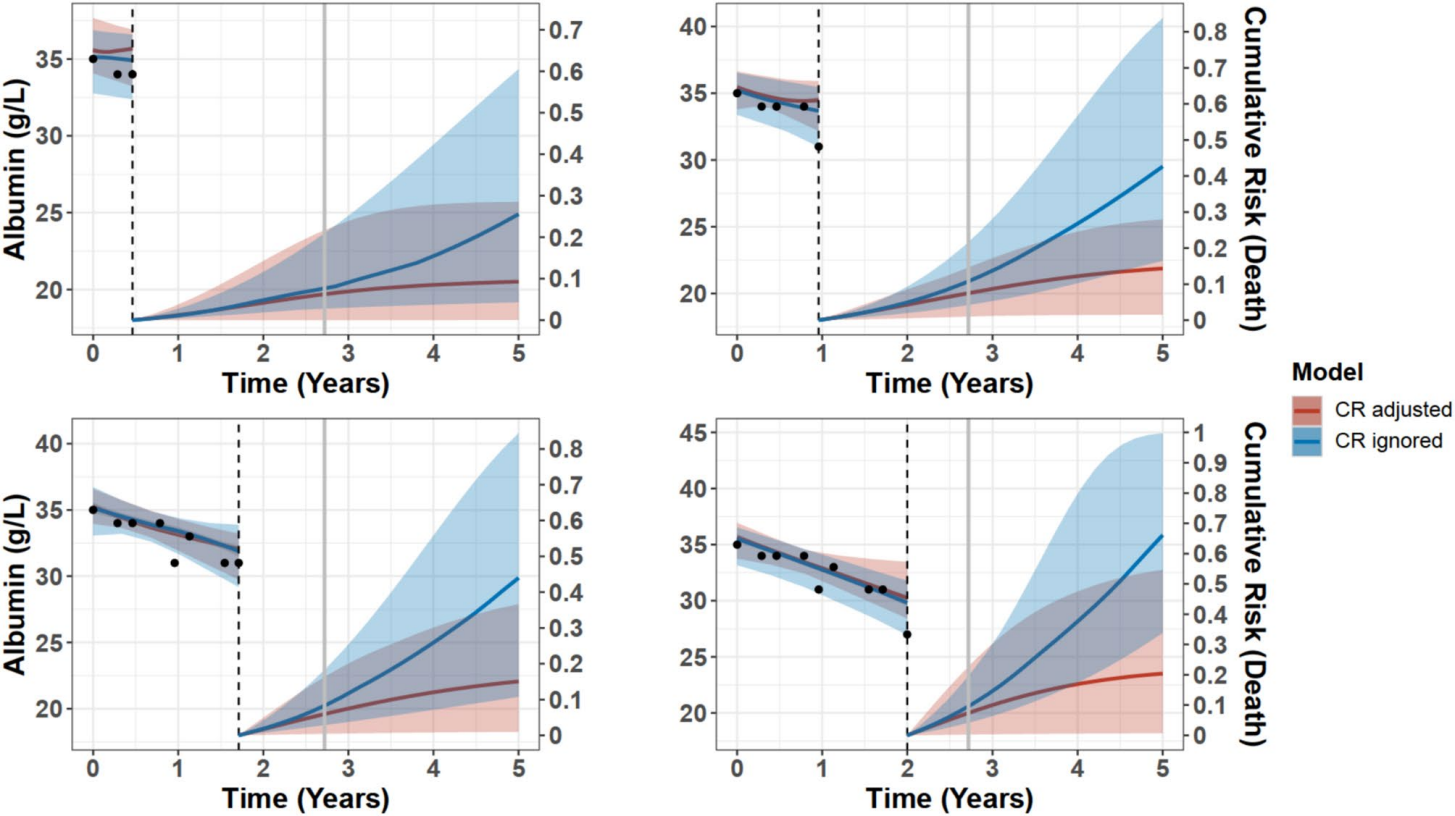
Dynamic predictions for a patient selected at random from the IDEAL trial PD testing dataset calculated with the two models with best fit. Results from best-fitting model without competing risks ‘t-t-mod3’ are in blue and results from the best-fitting model with competing risks, ‘t-t-mod3-cr’, are in red. The x-axis represents years after PD start, and the vertical dotted line indicates the time point of the latest measurement. The y-axis of the left side represents the albumin measurements that are available up to the latest visit. In particular, the black circles represent the observed values and the solid line the fitted longitudinal trajectory. The y-axis on the right side represents the mean estimator of the predicted cumulative risk of death and the shaded area illustrates the corresponding 95% pointwise Bayesian intervals. The grey vertical line indicates the occurrence of event to the patient, who was transferred to HD at time = 2.72 years after start of PD.

In terms of calibration, Brier scores were similar across the 12 JM (Fig. S11), with a moderate advantage for JM adjusted for competing risks. Calibration plots demonstrated deciles of predicted risk closer to observed death rates for competing events adjusted JM (Fig. S13 – S18). The rationale behind improved survival prognosis when accounting for competing risks is illustrated in Fig. 5. For a patient who switched to HD, ignoring competing events led to an overestimation of death probability across all forecast periods. Moreover, Fig. S12 shows a PD patient who eventually died; their death prediction was slightly higher (and thus more accurate) than the one obtained from JM ignoring competing risks. Similar conclusions about the benefits of adjusting for competing risks were validated in a simulation study, which nearly replicated the dynamic prediction findings from the IDEAL PD testing dataset, especially in the 1-year forecast window (Supp. 4.3).

### JM application in the HD population

Among baseline characteristics of the IDEAL trial HD training dataset (Table S14), variable selection identified age, cardiovascular disease, smoking status and statin use as baseline covariates in the survival sub-model (Suppl. 3.9). With only 14 patients transferred to PD (6% event rate), KTx was considered the single competing event to death, with baseline risk factors of age, ethnicity, and diabetes (Suppl. 3.9). As in PD, the ‘current value’ link function best modelled the association between longitudinal albumin and all-cause mortality (Table S16). The Q-Q plot of the standardized conditional residuals showed departures from the normal distribution assumptions (Fig. S20). Again, the best-fitting models were ‘*t-t-mod3*’ and ‘*t-t-mod3-cr*’, while the standard JM was ranked last (Table 3), indicating that the presence of both *b*- and *e*-outliers in albumin trajectories in HD patients, too. The albumin HR for death was consistent across models, showing a strong negative association between longitudinal albumin and risk of death in HD patients, similar to the PD cohort (posterior mean HR of 1.19 to 1.20 per 1 g/L albumin decline, Fig. 3B and Tables S17, S18).

Prediction accuracy for all-cause mortality in the HD testing dataset mirrored patterns observed with JM in PD. Prediction discrimination increased with longer follow-up, indicated by higher AUC values at later landmark times. Adjustment for the competing risk of KTx improved both discrimination and calibration of predictions (lower BS values) across all landmark and prediction horizons for each albumin outlier structure (Tables 5 and S18). All 12 JM outperformed the two Cox models across all landmark times and forecast horizons up to 5 years on dialysis, in terms of both discrimination and calibration of prediction accuracy (Tables 5 and S20). While the datasets were divided in a 3-to-1 training-versus-testing ratio for both PD and HD analyses, a proportion commonly used in JM research^30^, repeating the analyses with a 4-to-1 split produced virtually identical results.

**Table 5:** Area under the curve (AUC) of dynamic predictions for three landmark times and various prediction horizons with IDEAL HD testing dataset.

Landmark time (Year)	Forecast Time (Year)	Cox-1	Cox-2	nor	nor-cr	t-t-mod1	t-t-mod1-cr	t-t-mod2	t-t-mod2-cr	nor-t-mod2	nor-t-mod2-cr	t-t-mod3	t-t-mod3-cr	nor-t-mod3	nor-t-mod3-cr
1	1.5	0.85	0.84	0.84	0.87	0.85	0.86	0.83	0.86	0.87	0.86	0.85	0.86	0.86	0.87
	2	0.76	0.76	0.79	0.80	0.79	0.79	0.78	0.79	0.79	0.79	0.80	0.79	0.80	0.80
	2.5	0.75	0.77	0.79	0.82	0.79	0.81	0.80	0.81	0.80	0.80	0.79	0.81	0.80	0.81
	3	0.78	0.81	0.85	0.86	0.83	0.85	0.85	0.84	0.83	0.85	0.84	0.84	0.84	0.84
	3.5	0.78	0.82	0.84	0.86	0.82	0.85	0.83	0.85	0.82	0.85	0.83	0.84	0.82	0.84
	4	0.81	0.85	0.88	0.89	0.87	0.88	0.87	0.88	0.86	0.88	0.87	0.88	0.86	0.88
	4.5	0.80	0.88	0.91	0.92	0.89	0.91	0.90	0.91	0.88	0.90	0.89	0.90	0.88	0.90
	5	0.76	0.86	0.89	0.92	0.87	0.91	0.88	0.90	0.86	0.90	0.88	0.90	0.87	0.90
1.5	2	0.71	0.75	0.78	0.80	0.79	0.79	0.79	0.78	0.78	0.79	0.79	0.79	0.79	0.77
	2.5	0.71	0.75	0.78	0.81	0.79	0.80	0.79	0.78	0.79	0.79	0.79	0.79	0.78	0.79
	3	0.76	0.80	0.84	0.86	0.85	0.86	0.84	0.84	0.83	0.85	0.83	0.84	0.83	0.84
	3.5	0.76	0.78	0.82	0.85	0.82	0.86	0.81	0.83	0.81	0.83	0.82	0.83	0.81	0.83
	4	0.80	0.77	0.85	0.87	0.84	0.88	0.84	0.86	0.84	0.86	0.84	0.86	0.84	0.86
	4.5	0.79	0.79	0.87	0.90	0.86	0.90	0.87	0.88	0.87	0.88	0.87	0.88	0.87	0.89
	5	0.75	0.76	0.86	0.89	0.84	0.89	0.85	0.88	0.85	0.87	0.85	0.88	0.85	0.88
2	2.5	0.76	0.66	0.76	0.80	0.74	0.77	0.78	0.77	0.78	0.77	0.79	0.79	0.78	0.79
	3	0.79	0.83	0.87	0.90	0.87	0.89	0.88	0.89	0.87	0.88	0.86	0.87	0.87	0.87
	3.5	0.80	0.81	0.83	0.88	0.85	0.87	0.85	0.86	0.84	0.86	0.84	0.85	0.83	0.85
	4	0.83	0.79	0.84	0.86	0.85	0.87	0.82	0.86	0.85	0.86	0.83	0.86	0.84	0.85
	4.5	0.81	0.84	0.87	0.89	0.89	0.90	0.85	0.90	0.88	0.90	0.88	0.89	0.88	0.89
	5	0.78	0.81	0.85	0.88	0.87	0.88	0.84	0.89	0.86	0.88	0.86	0.88	0.85	0.88

## Discussion

Our study pioneers in the use of advanced JM in dialysis patients, considering baseline risk profile, surpassing standard distribution assumptions and adjusting for competing risks. The JM generate individualized survival predictions using longitudinal trends in plasma albumin. They consistently achieved superior predictive accuracy over traditional Cox models in PD patients and proved to be valid in simulation studies and in the HD patient cohort. Previous applications of standard JM in dialysis^18,22,23,34–37^ ignored outliers and the two studies which considered dynamic predictions^34,38^ did not account for competing risks, nor compared findings to established methods, i.e. Cox models.

Serum albumin has emerged as a multi-faceted, potent predictor of all-cause mortality^5,36,37,39^ due to its association with important physiological and clinical factors. Albumin serves as a marker for malnutrition and protein-energy wasting in dialysis patients^40^. Inflammatory processes hamper hepatic albumin synthesis and elevate albumin degradation, and reduce serum albumin concentrations, which contributes to oedema and fluid overload^40,41^. Utilizing our innovative Bayesian software, we observed minimal sensitivity in albumin hazard ratio estimates across various outlier structures and the adjustment for competing risks. After accounting for identified baseline risks factors, which are well in line with previous studies^42,43^, a 1 g/L decrease in plasma albumin was consistently associated with a more than 20% increased risk of all-cause death in PD and HD patients. Our results indicate that sustained decreases in albumin monitored over longer time periods rather than single outliers are suggestive of worse patient survival even after accounting for competing risks. Validation in two simulation demonstrated close similarity with observed clinical data patterns. Our results provide further evidence on the role of longitudinal albumin in mortality risk in dialysis patients, as suggested in two large-scale clinical trials using the extended Cox model^14,16^ and in studies using the standard JM with normal distribution assumption in registry datasets^18,23,34,38,44^.

Our findings now show that albumin decline is not only statistically significant but clinically actionable. A sustained 4 g/L drop in albumin, an amount commonly seen in the year before death in dialysis patients, translates, under our JM hazard ratio of 1.22 per g/L, into about a 2.2-fold increase in the instantaneous risk of death (≈120% higher hazard). Practically, a typical 60-year-old PD patient whose albumin stays at 38 g/L has a 9% twelve-month mortality risk; if albumin drifts to 34 g/L that risk rises to about 19%, and at 30 g/L to around 37%. Our framework could turn calculated individual dynamic predictions at each visit into bedside prompts, flagging patients whose one-year mortality now surpasses a clinically relevant threshold (e.g. 25%) and triggering timely nutrition review, dialysis intensification, or transplant evaluation while an intervention window remains open.

Leveraging one of the largest randomized trials in dialysis, the IDEAL trial, we examined albumin trajectories and found two distinct types of outliers in both PD and HD populations. First, some patients sit persistently above or below the population curve. The *t*-distributed JM ‘*t-t-mod3*’ captures these as *b*-outliers^21^ through an inflated subject-level mixing term (Supp. 3.5). Second, brief downward spikes arise in otherwise stable profiles; these appear as *e*-outliers^21^ because only the observation-level weight is inflated (Supp. 3.5). Such transient dips are common during intercurrent infections or short-lived measurement artefacts. By relaxing the usual normality assumption, the heavy-tailed Student-*t* specification automatically down-weights these short episodes, preventing them from distorting the albumin trajectory estimate and thus the patient’s survival prediction, while still flagging sustained albumin decline that carries prognostic significance (Supp. 3.5). Similar benefits of robust joint modelling have been reported in kidney-graft survival and primary biliary cirrhosis^20,21^, underscoring the general value of this approach.

The subsequent development of JM additionally adjusting for prevailing competing events, substantially improved prediction in both PD and HD patients. In PD and more so in the HD cohort, predictive accuracy was largely within the ‘excellent’ category^3^, indicated by an AUC range of 0.80–0.89 and, depending on the landmark time and prediction horizon, even reached the ‘outstanding’^3^ category with an AUC of 0.90 or higher. These predictions substantially exceeded the two standard Cox models for both PD and HD patients, a comparison which has been ignored in previous studies in dialysis patients and largely ignored in other disease settings^30–32,34,38^. Cox models, the benchmark for both etiological as well prognostic modelling, are subject to inherent limitations. Cox models with baseline risk factors are static as they do not incorporate dynamic follow-up information. As part of this study with PD and HD patients and the respective simulation study, albumin taken at baseline, along with other baseline risk factors, does not provide a reliable prediction modelling framework. The extended Cox model which incorporates time-varying albumin offers an improvement, however, it implicitly assumes the last available biomarker value as constant due to a ‘*Last Value Carried Forward*’ imputation and that the biomarkers are measured perfectly and free from biological variability^19^. Our advanced JM resolves these shortcomings. Another strength of this study is the creation of software for both JM estimation and dynamic predictions, simultaneously adjusting for both *b*- and *e*-outliers in longitudinal biomarkers and competing risks. The proposed software solution results in markedly shorter estimation times and greater sampling efficiency than the recent **robjm**^20,21^ package. As larger clinical datasets could render existing software packages computationally impractical, our advanced computational solution fosters the wider application of JM and can universally be applied to dialysis and other therapeutic areas. The extensive supplementary material comprehensively informs on JM building, selection and diagnostics (Suppl. Section 2).

Our study faces several limitations. Although IDEAL provides one of the largest randomized dialysis cohorts with > 600 patients and > 6800 albumin measurements, we acknowledge the need for external validation. To gauge robustness beyond this sample we ran three extensive simulation experiments (Supplement Sect. 4) that generated a total of 200 synthetic cohorts and 140 independent test patients. In every scenario the joint models recovered true parameters with negligible bias and maintained superior prediction accuracy over Cox comparators. Building on these encouraging results, we are now negotiating access to validate our framework externally in the multinational PDOPPS^45^ registry (> 1000 patients with serial laboratory data). Furthermore, the IDEAL trial data used were collected more than a decade ago, thus not incorporating the latest clinical practice recommendations. We aim to address this concern with the PDOPPS dataset as a more contemporary study that captures practice patterns from centers around the world, allowing confirmation of our findings under currently implemented dialysis protocols. This step-by-step strategy, moving from the trial dataset to in-silico expansion and then to upcoming real-world replication, offers a practical path from developing the method to putting it to work in the clinic. A future step to support widespread use is packaging the validated models in a lightweight, browser-based calculator. The web tool will take albumin and baseline data, run the model on a secure server, and return an updated survival curve plus simple “traffic-light” alerts (e.g., red if 1-year mortality > 25%). Finally, greater prediction accuracy may be achieved by adding further longitudinal biomarkers to the JM structure such as residual kidney function^15,16^, and systemic inflammation markers (C-reactive protein and interleukin −6)^46^.

In conclusion, our advanced JM, combining established baseline risk factors with individual albumin trajectories, and multidimensional consideration of outliers and competing risks, substantially improves accuracy of mortality prediction in both PD and HD patients^47–49^. Together with the speed- and efficiency-optimized software we provide essential prerequisites for superior large-scale prediction in clinical data sets. It is a major step forward to an individualized and dynamic dialysis patient outcome, well-aligned with the personalized approaches for the monitoring and management of chronic diseases^38^.

## Supplementary Information

Supplementary Information.

## Data Availability

The clinical trial data underlying this article were provided by the University of Sydney to the University Hospital Heidelberg under the terms of a Material Transfer Agreement. The data underlying the simulation studies as part of the Supplementary file in this article will be shared on reasonable request to the corresponding author.
