# Supplementary material for: Dynamic mortality predictions from serum albumin in dialysis patients using robust joint models with competing risks
Source: Sci Rep. 2025 Oct 8;15:35046. doi: 10.1038/s41598-025-21626-x (PMC12508142; doi:10.1038/s41598-025-21626-x)
Supplement: Supplementary file 1 — Supplementary Information. [file 41598_2025_21626_MOESM1_ESM.pdf]

DYNAMIC MORTALITY PREDICTIONS FROM SERUM ALBUMIN IN DIALYSIS PATIENTS USING ROBUST JOINT MODELS WITH COMPETING RISKS

Ivan Damgov[1,2], Meinhard Kieser[2], Peter Rutherford[3], Simon J. Davies[4], Muh Geot Wong[5], Carol Pollock[6], David W Johnson[7,8], Claus Peter Schmitt[1]

[1]Center for Pediatric and Adolescent Medicine, University of Heidelberg, Heidelberg, Germany.

[2]Institute of Medical Biometry, University of Heidelberg, Heidelberg, Germany.

[3]Baxter Healthcare Corporation, Zurich, Switzerland.

[4]Faculty of Medicine and Health Sciences, Keele University, Stoke-on-Trent, UK.

[5]Department of Renal Medicine, Concord Repatriation General Hospital, University of Sydney, Concord, Australia.

[6]Kolling Institute, Sydney Medical School, University of Sydney, Royal North Shore Hospital, St Leonards, New South Wales, Australia.

[7]Australasian Kidney Trials Network, University of Queensland, Brisbane, Australia,

[8]Department of Kidney and Transplant Services, Princess Alexandra Hospital, Brisbane, Australia.

Address for correspondence:

Claus Peter Schmitt, MD, PhD

Division of Pediatric Nephrology

Center for Pediatric and Adolescent Medicine

Im Neuenheimer Feld 430

69120 Heidelberg, Germany

Phone +49-6221-56-39313

Fax: +49-6221-56-4203

E-mail: claus.peter.schmitt@med.uni-heidelberg.de

Contents

1. Methods Supplement 1

1.1. Study Flowcharts 1

1.2. Covariate selection 3

1.3. Robust Joint Models of Longitudinal and Time-to-event Outcomes 5

1.3.1. Notation and Statistical Framework of the Standard Joint Model 5

1.3.2. Robust Joint Models 7

1.3.3. Bayesian Inference 9

1.3.4. Extending Robust Joint Models with Competing risks 10

1.4. Model Diagnostics and Selection 12

1.5. Dynamic Individual Predictions from Joint Models 12

1.5.1. Dynamic Predictions from Robust Joint Models with a Single Time-to-Event Outcome 12

1.5.2. Dynamic Predictions from Robust Joint Models under Competing Risks 14

2. Innovative Computational Solution 16

3. Results Supplement 18

3.1. Baseline Characteristics of PD Training Dataset 18

3.2. Selecting Association Structure for JM 21

3.3. Checking the Appropriateness of Weibull PH Parametric Model for All-Cause Mortality Baseline Hazard 23

3.4. Parameter Estimates and Estimation Properties of JM Fitted to the PD Training Dataset 25

3.5. Outlier Detection and Identification with Robust Joint Models 31

3.6. Results from Cox PH Models for All-cause Mortality 36

3.7. Additional Results from Dynamic Predictions for Various Prediction Horizons With IDEAL PD Testing Dataset. 37

3.8. Calibration Plots for Dynamic Predictions in IDEAL PD Testing Dataset 41

3.9. Summary of Results from the Application of Novel JM to the HD Population of the IDEAL Trial 47

4. Simulation Studies ... 61

4.1. Simulation Study to Assess Robust JM without Competing Risks ... 61

4.2. Simulation Study to Assess Robust JM with Competing Risks ... 66

4.3. Simulation Study to Assess Accuracy of Dynamic Predictions ... 70

References ... 74

1. Methods Supplement

1.1. Study Flowcharts

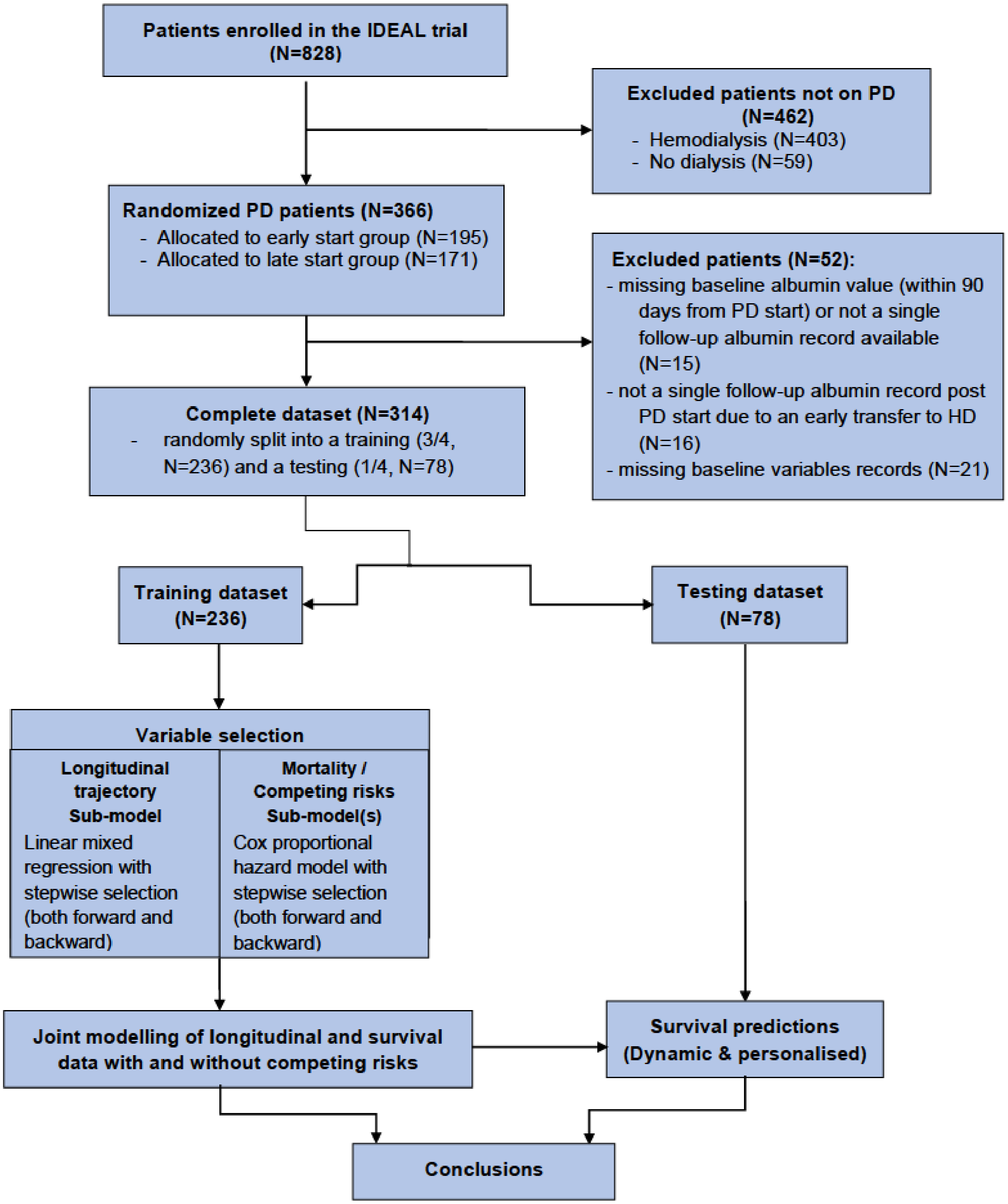

Figure S1. Detailed PD study flow diagram. N: number of patients; HD: hemodialysis; PD: peritoneal dialysis.

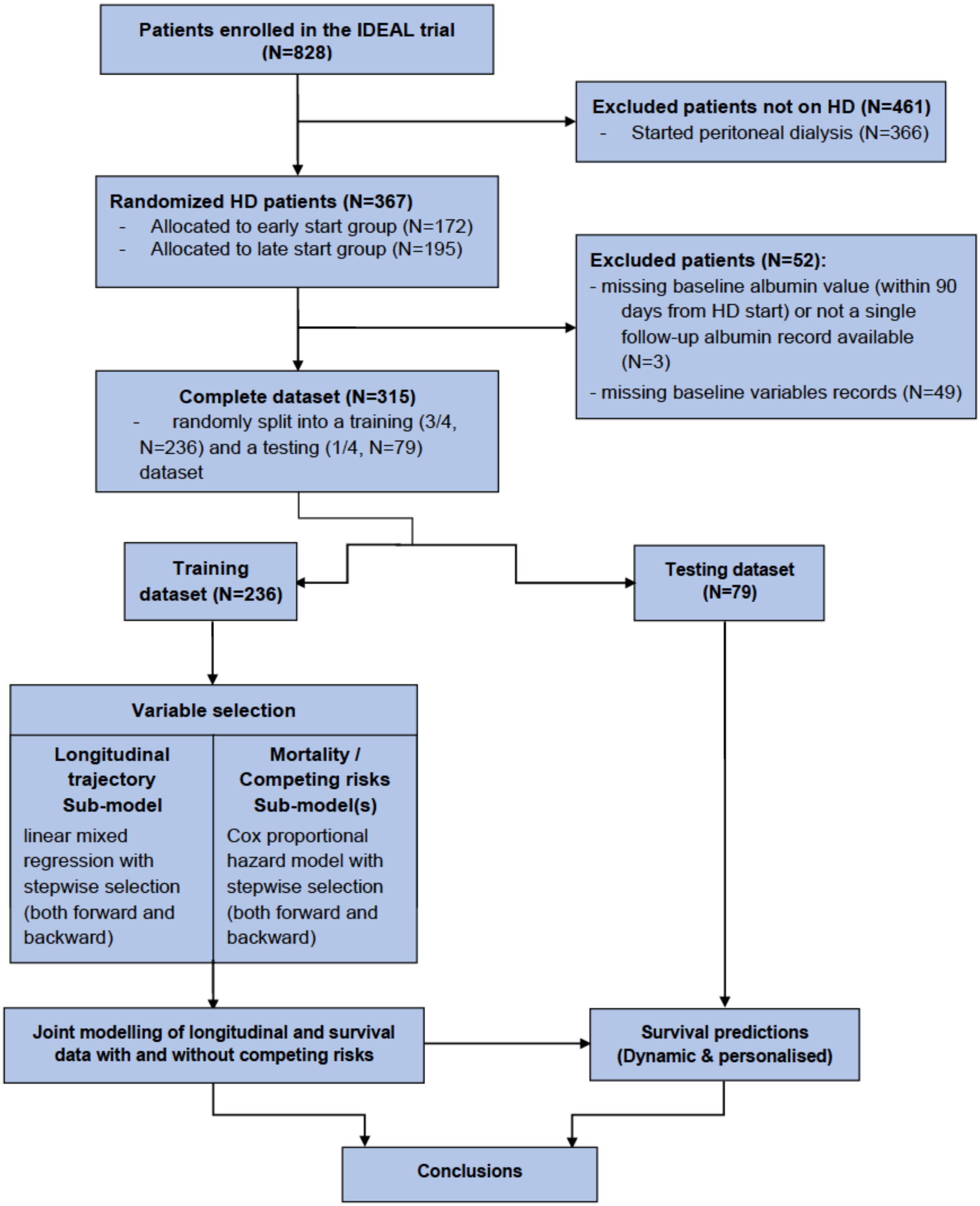

Figure S2. Detailed HD study flow diagram. N: number of patients; HD: hemodialysis; PD: peritoneal dialysis.

1.2. Covariate selection

The longitudinal sub-model for albumin is a component of the joint model (JM), delineating the temporal trajectory of the parameter observed at each follow-up. A mixed-effects model was employed to capture both patient-specific responses over time and the average evolution for the population. Various specifications for the time trend were explored, encompassing linear and quadratic terms, as well as flexible spline specifications, in both fixed and random (subject effects) of time. The final specification opted for a linear mixed-effects model with both a random intercept and a random slope. The event sub-models (survival, time to transfer to hemodialysis (HD), and kidney transplantation (KTx)) were initially constructed under the classical Cox proportional hazards framework, ensuring the proportionality of hazards assumption for baseline risk factors across all models using the *cox.zph()* function in **R**. The final specifications of the longitudinal and time-to-event sub-models involved considering various covariates, including demographic, comorbidity, biochemical, therapy-related, and medication variables at baseline. The complete variable list is presented in Figure S1 below. In accordance with recent guidelines for constructing joint models[1], the selection of baseline covariates was carried out separately for each sub-model as an initial step. For the albumin longitudinal sub-model, covariate selection occurred subsequent to specifying the time trend as a linear trajectory, using both forward and backward selection with a threshold P-value of 0.05 through the *stepAIC* function from the **MASS** package in **R**[2]. In the survival sub-model, as well as the time-to-event sub-models for transfer to HD and KTx, baseline risk factors were chosen using the **My.stepwis**e package in **R** (https://cran.r-project.org/web/packages/My.stepwise) assuming a Cox proportional hazards model, whereby this selection involved both forward and backward procedures with a threshold P-value of 0.05. The final covariates chosen for each sub-model are detailed in the right panels of Figure S3.

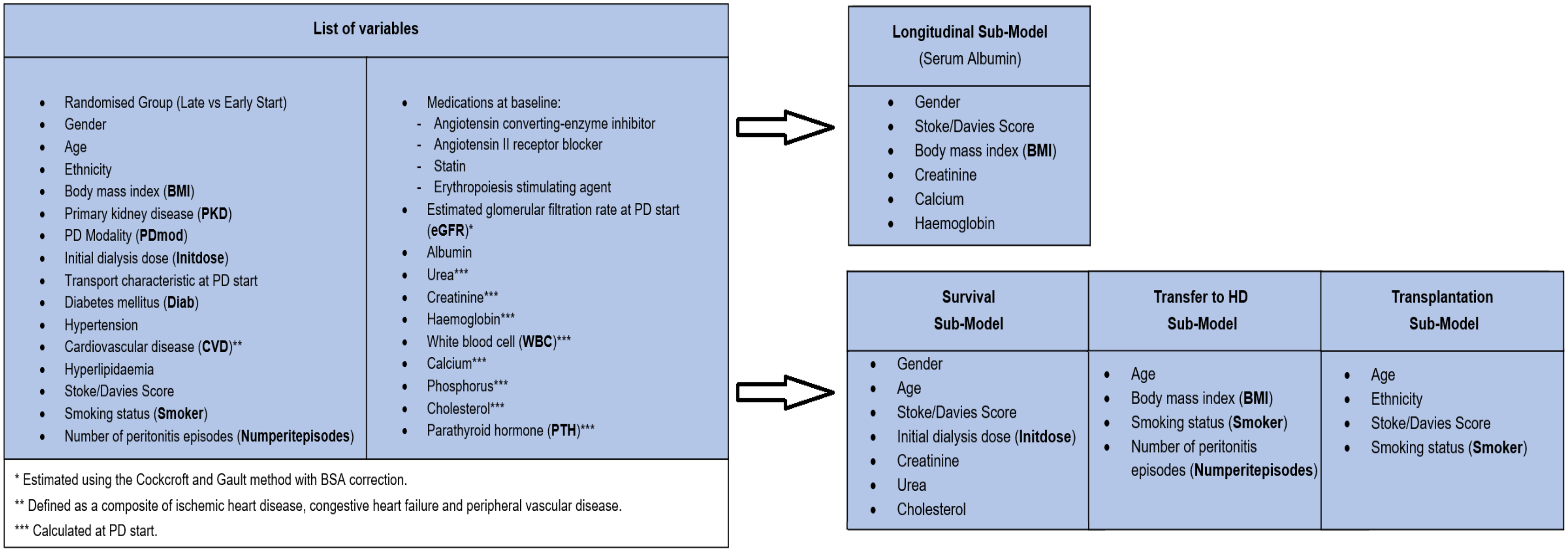

Figure S3. Variable selection to obtain baseline explanatory variables for each sub-model as part of the JM applied to the IDEAL trial PD training dataset.

1.3. Robust Joint Models of Longitudinal and Time-to-event Outcomes

1.3.1. Notation and Statistical Framework of the Standard Joint Model

We consider a sample of n independent and identically distributed subjects, each with available data $\{T_i, E_i, Y_i, t_i, a_i;\ i = 1, \dots, n\}$. In this context, T_i represents the time from the origin to the occurrence of the event of interest or right censoring, that is, $T_i = \min(T_i^*, C_i)$, whereby the true time to event (e.g. survival time) for subject i is denoted by T_i^* and C_i is the censoring time, which is defined as $C_i = \min(C, D_i)$, with study end time denoted by C and D_i the drop-out time. The event indicator, E_i, is expressed as $1\{T_i^* \leq C_i\}$ where $1\{\cdot\}$ is the indicator function, equal to 1 in the case of event occurrence and 0 otherwise, providing information on the subject's event status. The set of longitudinal marker measurements, $Y_i = \{Y_{ij};\ j = 1, \dots, m_i\}$, is collected at corresponding timings, $t_i = \{t_{ij};\ j = 1, \dots, m_i\}$ and is accompanied by baseline explanatory variables $a_i = \{a_{ik};\ k = 1, \dots, l\}$. Throughout this study, we focus on the shared-parameter version of the joint model, as initially introduced in earlier literature[3,4] and further popularised by a seminal book on the subject[5], encompassing the following components:

$$Y_{ij} = Y_i^*(t_{ij}) + Z_{ij} = \boldsymbol{x}_{ij}'\boldsymbol{\alpha} + \boldsymbol{d}_{ij}'\boldsymbol{b}_i + Z_{ij} = \boldsymbol{x}_{ij}'\boldsymbol{\alpha} + b_{0i} + b_{1i}t_{ij} + Z_{ij} \tag{1}$$

$$h_i(t) = h_0(t)\exp\left(\boldsymbol{c}_i'\boldsymbol{\omega} + g(Y_i^*(t); \boldsymbol{\eta})\right). \tag{2}$$

The longitudinal sub-model, represented by equation (1), encompasses a linear mixed-effects model that allows biomarker values to be decomposed into an underlying continuous-time signal at time t, $Y_i^*(t_{ij})$, and measurement error, Z_{ij} (within-subject or intra-individual heterogeneity). The 'true' signal', $Y_i^*(t_{ij})$, itself consists of fixed effects, $\boldsymbol{x}_{ij}'\boldsymbol{\alpha}$ (with $\boldsymbol{x}_{ij} = [\boldsymbol{x}_{ij1}, \dots, \boldsymbol{x}_{ijp}]$' being the design matrix with elements from t_i and a_i), and random effects, $\boldsymbol{d}_{ij}'\boldsymbol{b}_i$ (with $\boldsymbol{d}_{ij} = [\boldsymbol{d}_{ij1}, \dots, \boldsymbol{d}_{ijq}]'$ another design matrix that is typically a subset of elements of $\boldsymbol{x}_{ij}$), whereby $\boldsymbol{\alpha} = [\alpha_1, \dots, \alpha_p]$' are the population-averaged effects and $\boldsymbol{b}_i = [b_{i1}, \dots, b_{iq}]'$ are the subject-specific random effects (i.e. latent variables) that account for the heterogeneity between subjects (i.e. inter-individual heterogeneity). A linear time trend is assumed with random intercept (b_{0i}) and random slope (b_{1i}), though more complex specifications like polynomials or splines are possible. Of note, the standard assumptions for the random effects and error terms are that they follow Gaussian distributions with mean zero[6], where $\boldsymbol{\Sigma}$ is some unstructured variance-covariance matrix and let N and MVN denote the normal and multivariate normal distributions, respectively:

$$\boldsymbol{b}_i \sim MVN(\boldsymbol{0}, \boldsymbol{\Sigma}_{\mathbf{qxq}}),\ \ Z_{ij} \sim N(0, \sigma^2),\ \ \boldsymbol{b}_i \perp Z_{ij},\ Z_{ij} \perp Z_{ij'}\ for\ j \neq j'. \tag{3}$$

The second 'building block' of the JM methodology is the time-to-event outcome, outlined in equation (2). It follows the usual practice in JM literature, modelling the hazard of the event using a proportional hazard (PH) specification (also known as relative risk or relative hazard models). The hazard of the event for an individual i at time t, $h_i(t)$, consists of $h_0(t)$, as the baseline hazard at time t (the hazard for an individual when all covariates are equal to 0) and $\boldsymbol{c}_i'$, a vector of risk factors with multiplicative effects on the hazard of an event, represented by population-level (fixed effects) parameters $\boldsymbol{\omega}$. The elements of $\boldsymbol{c}_i'$ may or may not coincide with $\boldsymbol{x}_{ij}'$ from the longitudinal sub-model. Equation (2) also includes the term $g(Y_i^*(t); \boldsymbol{\eta})$ representing the link or association structure between the underlying signal up to and including time t, $Y_i^*(t)$, using a pre-defined link function $g(\cdot)$ and parameters $\boldsymbol{\eta}$.

The selection of $h_0(t)$ has been extensively discussed in previous literature. In contrast to standard survival analysis employing the Cox Proportional Hazards (PH) model, where $h_0(t)$ remains undefined to avoid

potential misspecification of survival times, this approach has been considered unfavourable in early JM development due to the underestimation of standard errors of parameter estimates[5,7]. Therefore, JM literature has opted for an explicit definition of $h_0(t)$ tailored to the patterns of time-to-event data under analysis. This study adopts a parametric model for $h_0(t)$ using the Weibull distribution, represented as:

$$h_0(t) \ = \nu\lambda t^{\lambda-1}, \tag{4}$$

where ν and λ are the scale and shape parameters, respectively. Following the relative risk specification in equation (2), the time-to-event sub-model is specified as $h_0(t) = \lambda t^{\lambda-1}$, and the logarithm of ν enters the design matrix $\boldsymbol{c}_i'$ for the baseline covariates as the intercept term. This is known as the Weibull Proportional Hazards (PH) specification found in survival literature. Notably, the Weibull model has the unique property of being expressible under both the relative risk and accelerated failure time (AFT) formulations. However, it has been highlighted in previous literature that this property is lost as soon as time-dependent covariates are included in the model[5]. Therefore, a Weibull PH specification is followed in this work, which does not imply identity with the Weibull AFT model due to the presence of a time-dependent biomarker as a covariate. Consequently, $\exp(\boldsymbol{\omega})$ represents the ratios of hazards for a one-unit change in baseline risk factors $\boldsymbol{c}_i'$, which are also known as hazard ratios in classical survival analysis. This allows for a direct comparison of the effect of a covariate on the risk of an event estimated from the developed joint models with traditional Cox models.

Moreover, the association structure $g(Y_i^*(t);\boldsymbol{\eta})$ has been a subject of interest in previous literature, defining the dependence between the longitudinal and event sub-models. The most popular choice is the 'current value' parameterization:

$$g(Y_i^*(t);\boldsymbol{\eta}) = \eta Y_i^*(t)\,. \tag{5}$$

This structure posits a link between the log hazard of the event and the expected value of the biomarker, as opposed to the directly observed biomarker value. In simpler terms, $\exp(\eta)$ is the hazard ratio for a unit increase in the expected value $Y_i^*(t)$ of the biomarker, making it directly interpretable. This choice is significant for two primary reasons[8]: it aligns with the notion that the biomarker unfolds continuously over time, despite intermittent discrete observations, and it eliminates the influence of measurement errors inherent in observed biomarker data from the association between the biomarker and the event. This is crucial as earlier works have indicated the potential for measurement errors to introduce bias favouring null findings when estimating the log hazard ratio within a Cox proportional hazards model[9]. As a result, given an appropriate specification of the longitudinal sub-model, the joint model gives a meaningful link between the event's hazard and the intrinsic and error-free individual-specific value of the biomarker.

For a comprehensive understanding, other association structures have been proposed in the literature, detailed in Table S1, exploring additional features of the biomarker trajectory in the context of repeated measurements throughout patient follow-ups. These structures allow the investigation of hypotheses regarding the type of association between the biomarker and the event. The 'instantaneous slope' of the parameter trajectory may be of interest in some applications, denoted by $\eta\frac{\partial Y_i^*(t)}{\partial t}$, indicating a decreasing, increasing, or stable trajectory[10]. A combination of 'current value' and 'instantaneous slope,' represented as $\eta_1 Y_i^*(t) + \eta_2\frac{\partial Y_i^*(t)}{\partial t}$, enables the exploration of the additional predictive value of the direction in a patient's trajectory for the risk of an event, beyond the current value of the biomarker. This could be illustrated for any two patients having the same value of a biomarker but a different direction of change in the trajectory at time t. In contrast with the above association structures, which relate the hazard function to features of

the longitudinal sub-model at a given time point, the cumulative effects parametrization has received interest in prior literature[11]. It allows the investigation of the association between the entire history of the longitudinal process up to and including time t, and the event as the integral $\int_o^t Y_i^*(t)dt$ is included in the hazard model. The 'shared random effects' specification, discussed as a heritage from early works on joint models, allows for a simpler time-independent association between the biomarker trajectory and the hazard of an event, adding the random effects from the longitudinal sub-model as frailty terms in the hazard sub-model. Table S1 presents two such models in the case of the longitudinal model following a linear time trend with random intercept (b_0) and random slope (b_1). In the current implementation of JM in dialysis, a comparison in model fit between the above-mentioned association structures was performed as a first step in the process of JM selection/building. The current value association as per equation (5) is the one of highest relevance for this work as described in Section 3.1.

Table S1. Association structure forms for the shared-parameter joint model.

Association structure	Specification for $g(Y_i^*(t);\boldsymbol{\eta})$	Studies
Current value	$\eta Y_i^*(t)$	(Asar et al. 2021; Battes et al. 2015; Chen et al. 2014; Ghosh et al. 2011; Ibrahim et al. 2004; Köhler et al. 2018; Lin et al. 2002; McFetridge et al. 2021; Musoro et al. 2015; Rizopoulos and Ghosh 2011; Rizopoulos et al. 2014; van Boven et al. 2018)
Current slope	$\eta \frac{\partial Y_i^*(t)}{\partial t}$	(Andrinopoulou et al. 2017; Rizopoulos and Ghosh 2011; van Boven et al. 2018)
Current value + Current slope	$\eta_1 Y_i^*(t) + \eta_2 \frac{\partial Y_i^*(t)}{\partial t}$	(Asar et al. 2021; van Boven et al. 2018)
Cumulative effects	$\int_o^t Y_i^*(t)dt$	(Andrinopoulou et al. 2017; van Boven et al. 2018)
Shared random effects (b_0)	ηb_0	(Campbell et al. 2019)
Shared random effects (b_0, b_1)	$\eta_1 b_0 + \eta_2 b_1$	(Baghfalaki and Ganjali 2015; Campbell et al. 2019; Campbell et al. 2021; Hennessey et al. 2018)

1.3.2. Robust Joint Models

Equation (3) in the previous section outlined the standard assumptions in JM literature for the random effects and within-subject error in the longitudinal sub-model following zero-mean Normal distributions. That choice is certainly very popular in joint modelling literature for practicality reasons as it simplifies computations significantly. Yet, several authors have pointed that this assumption might be unrealistic when applied to actual clinical trial data with outliers at the patient level (b-outliers), whereby patients within the population do not conform to population trends, as well as outlying observations within an individual's set of measurements that do not follow the patient-specific trend over time (e-outliers)[6,12,13]. Parameter estimates could in fact be very sensitive to the presence of such outliers, especially when it comes to the association between the biomarker trajectory and the risk of event[6,12,13]. These recent JM publications have suggested the usage of heavy-tailed distributions for robust inferences, namely various parameterisations

of the Student's *t*-distribution to protect against *b*- and *e*-outliers and alleviate bias in parameter estimates and their associated standard errors from violations of normal distribution assumptions. In the absence of outliers, these models would still yield unbiased estimates as they would manage to approximate normality. The rest of this section provides three approaches to robust JM as outlined in recent literature[6] as the core methodological framework.

A key component for the robustness of the joint model is the tail behaviour of the distributions of $\boldsymbol{b}_i$ and Z_{ij}. The tail of $\boldsymbol{b}_i$ distribution governs robustness to *b*-outliers and Z_{ij} tail behaviour affects robustness to *e*-outliers. In this work symmetric robust distributions will be considered for both random-effects and measurement error terms represented as normal-variance mixtures as follows:

$$\boldsymbol{b}_i = \boldsymbol{\Sigma}^{1/2}\sqrt{V_i}\boldsymbol{b}_i^*, \qquad Z_{ij} = \sigma\sqrt{W_{ij}}Z_{ij}^*, \tag{6}$$

where $\boldsymbol{b}_i^* \sim MVN(\mathbf{0}, \mathbf{I}_{qxq})$, $Z_{ij}^* \sim N(0,1)$ and $\boldsymbol{b}_i^* \perp Z_{ij}^*$. Thus, special attention is placed on V_i and W_{ij} for random-effects and measurement error components, respectively. As noted in prior literature, the specification in (6) is flexible as it allows for widely used distributions as special cases[6,14]. For example, $V_i = W_{ij} = 1$ recovers the standard assumptions for the random effects and error terms following Gaussian distributions with mean zero as presented in Equation (3) in previous section, denoted by '*nor-nor*' in the joint model nomenclature throughout this work. Hereby, an inverse Gamma distribution (IG) with equal shape and scale parameters is assumed for V_i and W_{ij}. A well-known result, explored extensively in robust mixture model literature, is that in this case $\boldsymbol{b}_i$ and Z_{ij} then follow the heavy-tailed *t*-distribution. Yet, various options in the way to model *t*-distribution were presented in joint model literature so far, constructed under more or less restrictive assumptions.

More formally, the first approach (in an order of ascending complexity) was presented in the work of McCrink (2014)[15] with components:

$$W_{ij} = V_i, \qquad V_i \sim IG(\gamma/2\,,\gamma/2)\,. \tag{7}$$

In this simplified approach, $\boldsymbol{b}_i$ and $\boldsymbol{Z}_i$ are jointly multivariate *t* with single degrees-of-freedom parameter γ and the independence properties from Eq. (3), $\boldsymbol{b}_i \perp Z_{ij}$ and $Z_{ij} \perp Z_{ij'}, for\ j \neq j'$, are not satisfied. Conditioning on V_i then allows for independence between $\boldsymbol{b}_i$ and Z_{ij} as well as between Z_{ij} and $Z_{ij'}$ since $\boldsymbol{b}_i^* \perp Z_{ij}^*$ and $Z_{ij}^* \perp Z_{ij'}^*$. That joint model specification will be referred to as '*t-t-mod1*'.

Another approach comes from the work of Baghfalaki et al. (2013)[12], whereby the longitudinal sub-model specification of the joint model assumes:

$$V_i \sim IG(\phi/2\,,\phi/2) \text{ and } W_{ij} = W_i \sim IG(\delta/2\,,\delta/2). \tag{8}$$

Thus, the dependence $\boldsymbol{b}_i \perp Z_{ij}$ now holds unconditionally as $\boldsymbol{b}_i$ and $\boldsymbol{Z}_i$ follow separate with multivariate *t*-distributions (MVt) with degrees-of-freedom parameters ϕ and δ, respectively. Similarly to '*t-t-mod1*' above, Z_{ij} and $Z_{ij'}$ share a common W_i, hence they are only conditionally independent given W_i. That joint model specification will be referred to as '*t-t-mod2*'.

The final specification considered here was introduced in joint model literature in a recent work[16] and further applied in an association publication[6], which postulates:

$$V_i \sim IG(\phi/2\,,\phi/2) \text{ and } W_{ij} \sim IG(\delta/2\,,\delta/2). \tag{9}$$

This approach is the most flexible as it further removes the dependence between Z_{ij} and $Z_{ij'}$ so that both $\boldsymbol{b}_i \perp Z_{ij}$ and $Z_{ij} \perp Z_{ij'}\, for\ j \neq j'$ hold unconditionally and as in '*t-t-mod2*', $\boldsymbol{b}_i|\boldsymbol{\Sigma}, \phi \sim MVt\ (\mathbf{0}, \boldsymbol{\Sigma}, \phi)$ and $Z_{ij}|\sigma, \delta\ \sim t(0, \sigma^2, \delta)$. That joint model specification will be referred to as '*t-t-mod3*'. Hence, '*t-t-mod3*' approach allows for full flexibility in modelling the within-subject error structure by decoupling of error terms at different measurement times through the use of independent mixing variates. These properties of '*t-t-mod3*' can play a crucial role in the identification of e-outliers.

In addition, '*t-t-mod2*' and '*t-t-mod3*' approaches lend themselves to a modification, provided that one is not concerned with the occurrence of b-outliers. Instead of the multivariate t-distribution for the random-effects, one can then use a hybrid approach and combine multivariate normal random-effects (i.e. $\boldsymbol{b}_i \sim MVN(\mathbf{0}, \boldsymbol{\Sigma}_{\mathbf{qxq}})$) with either $Z_i|\sigma, \delta\ \sim t(0, \sigma^2, \delta)$ (as per model '*t-t-mod2*') or $Z_{ij}|\sigma, \delta\ \sim t(0, \sigma^2, \delta)$ (as per model '*t-t-mod3*'), resulting into models '*nor-t-mod2*' and '*nor-t-mod3*', respectively. A summary of specifications for these models are presented in the first six rows of Table 1 in main text. To conclude this section, it is important to outline that the mechanism through which presented robust JM account for the occurrence of outliers is through placing a weight on the individual random effect $(1/\sqrt{V_i})$ or within-subject error $(1/\sqrt{W_{ij}})$, which is lower than 1 (under the normal distribution), helping to alleviate the impact of outlying observations.

1.3.3. Bayesian Inference

We focus on Bayesian methods for estimation in this work as follows from recent literature on innovative and extended JM[16-18]. Of note, the standard joint model ('*nor-nor*') or specifications with normal random effects and t-distributed Z_{ij} terms (i.e. '*nor-t-mod2*' and '*nor-t-mod3*') or Z_{ij} terms being t-distributed Z_{ij} with $W_{ij} = W_i$ (i.e. '*t-t-mod2*' and '*nor-t-mod2*') can all be regarded as special cases of the most general '*t-t-mod3*' model. Therefore, notation below assumes a '*t-t-mod3*' specification.

Let $\mathbf{Y} = [Y_1', \dots, Y_n']'$ with $\mathbf{Y_i} = [Y_{i1}, \dots, Y_{im_i}]'$; $\mathbf{T} = [T_1, \dots, T_n]'$; $\mathbf{E} = [E_1, \dots, E_n]'$; $\mathbf{T} = [T_1', \dots, T_n']'$; $\mathbf{x} = [x_1', \dots, x_n']'$ with $\mathbf{x_i} = [x_{i1}, \dots, x_{im_i}]'$; $\mathbf{d} = [\mathbf{d}_1', \dots, \mathbf{d}_n']'$with $\mathbf{d_i} = [\mathbf{d}_{i1}, \dots, \mathbf{d}_{im_i}]'$; $\mathbf{b} = [\mathbf{b}_1', \dots, \mathbf{b}_n']'$; $\mathbf{V} = [V_1, \dots, V_n]'$ and $\mathbf{W} = [\mathbf{W}_1', \dots, \mathbf{W}_n']'$ with $\boldsymbol{W_i} = [W_{i1}, \dots, W_{im_i}]'$; $\boldsymbol{\zeta}$ the parameters of $h_0(t)$, which consist of λ in the case of the Weibull PH baseline hazards; $\mathbf{c} = [\mathbf{c}_1', \dots, \mathbf{c}_n']'$. the joint posterior density of parameters and individual-specific (i.e. latent) variables as follows a recent publication[16]:

$$p(\boldsymbol{\alpha}, \boldsymbol{\Sigma}, \sigma, \phi, \delta, \boldsymbol{\zeta}, \boldsymbol{\omega}, \boldsymbol{\eta}, \mathbf{b}, \mathbf{W}, \mathbf{x}, \mathbf{d}, \mathbf{c}|\mathbf{Y}, \mathbf{T}, \mathbf{E}) \propto p(\mathbf{Y}, \mathbf{T}, \mathbf{E}|\boldsymbol{\alpha}, \sigma, \boldsymbol{\Sigma}, \boldsymbol{\zeta}, \boldsymbol{\omega}, \boldsymbol{\eta}, \mathbf{b}, \mathbf{W}, \mathbf{x}, \mathbf{d}, \mathbf{c})$$

$$\times p(\mathbf{b}|\mathbf{V}, \boldsymbol{\Sigma}) p(\mathbf{V}|\phi) p(\mathbf{W}|\delta) \times p(\boldsymbol{\alpha}, \boldsymbol{\Sigma}, \sigma, \phi, \delta, \boldsymbol{\zeta}, \boldsymbol{\omega}, \boldsymbol{\eta}) \qquad (10)$$

whereby the decomposition of $p(\mathbf{Y}, \mathbf{T}, \mathbf{E}|\boldsymbol{\alpha}, \boldsymbol{\Sigma}, \sigma, \boldsymbol{\zeta}, \boldsymbol{\omega}, \boldsymbol{\eta}, \mathbf{b}, \mathbf{W}, \mathbf{x}, \mathbf{d}, \mathbf{c})$ into longitudinal $(p(\mathbf{Y}|\boldsymbol{\alpha}, \boldsymbol{\Sigma}, \sigma, \mathbf{b}, \mathbf{W}, \mathbf{x}, \mathbf{d}))$ and event sub-model $(p(\mathbf{T}, \mathbf{E}|\boldsymbol{\zeta}, \boldsymbol{\omega}, \boldsymbol{\eta}, \boldsymbol{\alpha}, \boldsymbol{\Sigma}, \sigma, \mathbf{b}, \mathbf{x}, \mathbf{d}, \mathbf{c}))$ contributions to the likelihood is enabled by the assumption of independence between longitudinal and event data provided the random-effects, parameters and explanatory variables. The conditional density of the longitudinal sub-model is then:

$$Y_i \,\big|\, \boldsymbol{\alpha}, \boldsymbol{\Sigma}, \sigma, \phi, \delta, \mathbf{b_i}, \mathbf{x}_{ij}, \mathbf{d}_{ij} \sim\ N\big(\mathbf{x}_{ij}'\boldsymbol{\alpha} + \mathbf{d}_{ij}\mathbf{b}_i, W_{ij}\sigma^2\big). \qquad (11)$$

The remaining components of Eq. (10) follow from Section 1.2.2, with $p(\mathbf{b}|\mathbf{V}, \boldsymbol{\Sigma}) \sim MVN(\mathbf{0}, \mathbf{V_i}\boldsymbol{\Sigma})$ and $p(\mathbf{V}|\phi)$ and $p(\mathbf{W}|\delta)$ are constructed from inverse-Gamma distributions, $IG(\phi/2\,, \phi/2)$ and $IG(\delta/2\,, \delta/2)$,

respectively. Assuming independent priors for the unknown variables, the joint prior of the parameters, $p(\boldsymbol{\alpha}, \boldsymbol{\Sigma}, \sigma, \phi, \delta, \boldsymbol{\zeta}, \boldsymbol{\omega}, \boldsymbol{\eta})$ is thus:

$$p(\boldsymbol{\alpha}, \boldsymbol{\Sigma}, \sigma, \phi, \delta, \boldsymbol{\zeta}, \boldsymbol{\omega}, \boldsymbol{\eta}) = p(\boldsymbol{\alpha})p(\boldsymbol{\Sigma})p(\sigma)p(\phi)p(\delta)p(\boldsymbol{\zeta})p(\boldsymbol{\omega})p(\boldsymbol{\eta}). \tag{12}$$

Finally, an important part of any Bayesian estimation approach is the definition of priors. Hereby, the choice of priors is entirely determined by recent application of robust JM with the **robjm** package[6,16] which considers weakly informative prior distributions for the parameters. All population effects in the longitudinal sub-model, $\boldsymbol{\alpha}$, are given zero-mean Cauchy with scale of 5, $\boldsymbol{\alpha} \sim C(0, 5)$ with the exception of the intercept term, α_0, which is assigned a scale of 20, $\alpha_0 \sim C(0, 20)$. $\boldsymbol{\Sigma}$ is decomposed as follows $\boldsymbol{\Sigma} = \mathbf{R\Omega R}$, where $\mathbf{R}$ is a diagonal matrix of scale elements of $\mathbf{b}$ and $\boldsymbol{\Omega}$ is a correlation matrix. The elements of $\mathbf{R}$ are given a half-Cauchy prior $C_{+}(0, 5)$ and the elements of $\boldsymbol{\Omega}$ are assigned Lewandowski-Kurowicka-Joe (LKJ) distribution with shape parameter equal to 2, $\boldsymbol{\Omega} \sim LKJ(2)$. Similarly to $\mathbf{R}$, the scale parameter for the residual error is assigned a half-Cauchy prior $C_{+}(0, 5)$, whereby in both cases the half-Cauchy is re-parameterised using a transformation of a uniform variable and the *tan()* function for a better sampling performance of the Stan algorithm. The degrees of freedom parameters ϕ and δ are given uniform priors between 2 and 100. Elements of $\boldsymbol{\zeta}, \boldsymbol{\omega}$ and $\boldsymbol{\eta}$ are similarly assigned the $C(0, 5)$ prior. In addition, to check sensitivity of estimates due to the choice of priors, alternative estimation was performed with weakly informative normal $N(0, 100)$ prior for elements $\boldsymbol{\alpha}, \boldsymbol{\zeta}, \boldsymbol{\omega}$ and $\boldsymbol{\eta}$ instead of the Cauchy prior and the unform prior for the degrees of freedom parameters ϕ and δ was replaced with a gamma prior proposed and analysed for Student's *t*-distribution in Bayesian literature[19] (results not shown).

1.3.4. Extending Robust Joint Models with Competing risks

When addressing competing events, it is possible to model two distinct risks: the cause-specific hazard (CSH) and the sub-distribution hazard (SDH). Although these two approaches fundamentally represent different parameterizations of the same stochastic process, some researchers contend that CSH parameters are better suited for etiological investigations, while SDH parameters are more appropriate for risk prediction[20-22]. Below, the cause-specific hazard is used to extend the formulation of the robust joint models, as the main methodological framework of choice in joint model literature with competing risks[5,23,24].

Continuing from Section 1.2.1, the joint model definition can be updated by replacing Eq. (2) with:

$$h_{ik}(t) = h_{0k}(t) \exp\left(\boldsymbol{c}'_{ik}\boldsymbol{\omega_k} + \eta_k Y_i^*(t)\right), \tag{13}$$

where $h_{ik}(t)$ denotes the cause-specific hazard for individual i at time t for an event type k $(1, \dots, K)$, $h_{0k}(t)$ is the cause-specific baseline hazard at time t for an event type k, $\boldsymbol{c}_{ik}$ is the vector of baseline risk factors assumed to affect event type k through a set of population-level parameters (i.e. log-hazard ratios) $\boldsymbol{\omega}_k$. As before, $Y_i^*(t)$ is the linear predictor of the longitudinal biomarker and η_k indicates the cause-specific association between the biomarker and the log hazard of each event type under a 'current value' association structure. Therefore, η_k is interpreted similarly to the model without competing risks: for an individual i who remains alive at time t, η_k gauges the relationship between a one-unit rise in the linear predictor for the biomarker and the logarithm of the cause-specific hazard for event type k (assuming all other covariates in the model remain unchanged). Building upon the parametric PH model for baseline hazards in Eq. (4) with a single time-to-event outcome, a Weibull PH model for the cause-specific hazard is assumed as follows:

$$h_{0k}(t) = \lambda_k t^{\lambda_k - 1}, \tag{14}$$

where λ_k is the cause-specific shape parameter and the logarithm of the cause-specific scale parameter ν_k is entered as the intercept term as part of the design matrix $\boldsymbol{c}'_{ik}$ for the baseline covariates. The inclusion of competing risks is then reflected in the conditional the density of the event sub-model as follows:

$$p(\mathbf{T}, \mathbf{E}|\boldsymbol{\zeta_k}, \boldsymbol{\omega_k}, \boldsymbol{\eta_k}, \boldsymbol{\alpha}, \boldsymbol{\Sigma}, \sigma, \mathbf{b}, \mathbf{x}, \mathbf{d}, \mathbf{c_k}) = \prod_{k=1}^{K} h_{ik}(s|\boldsymbol{b_i}, \boldsymbol{\Theta})^{E_{ik}} \exp\left(-\int_0^{t_{ik}} h_{ik}(s|\boldsymbol{b_i}, \boldsymbol{\Theta})\, ds\right), \quad (15)$$

where E_{ik} is the event indicator for an individual i and event type k, $\boldsymbol{\Theta}$ contains the unknown variables $\boldsymbol{\zeta_k}, \boldsymbol{\omega_k}, \boldsymbol{\eta_k}, \boldsymbol{\alpha}, \boldsymbol{\Sigma}, \sigma, \mathbf{x}, \mathbf{d}, \mathbf{c}$ with $\boldsymbol{\zeta_k}$ now containing λ_k parameters. Accounting for competing risks specification in the definition of the robust joint modes investigated in this work necessitates the addition of corresponding updated model names and definitions as shown in final six rows of Table 1 from the main text, which is a direct continuation of the JM counterparts without competing risks.

Estimation of the proposed JM was performed with Bayesian approaches using purpose-built programs with the R software[25]. Markov Chain Monte Carlo (MCMC) samples of the joint posterior are drawn using Hamiltonian Monte Carlo (HMC)[26], specifically employing the No-U-Turn Sampler (NUTS) algorithm[27], which is an adaptive version of HMC. The procedural framework is realized through the **R** package **cmdstanr** employing the HMC engine 'Stan'[28]. The proposed models with survival sub-model which ignore competing risks can be alternatively estimated by an existing package in **R**, the **robjm** package (https://github.com/ozgurasarstat/robjm), which also uses Bayesian approaches with the HMC engine 'Stan', however, with the help of the **rstan** package[29].

1.4. Model Diagnostics and Selection

In building the joint models, careful attention is required for constructing both longitudinal and time-to-event sub-models. The mixed-effects model used for longitudinal albumin estimates allows both patient-specific response over time as well as an average evolution in time for the population to be modelled. Various specifications of the time trend were explored in both fixed and random (subject effects) of time, including linear and quadratic terms, as well as flexible spline specifications. Linear mixed effects model, with both random intercept and random slope was chosen in the final specification. To perform diagnostics of the longitudinal sub-model, we present Residuals vs Fitted plot along with Q-Q plots of the standardised conditional residuals. The suitability of the Weibull PH model for the baseline hazard for death was assessed by a plot of estimated cumulative hazard vs Cox-Snell residuals as follows from recent guidance in JM literature[30].

The widely applicable information criterion (WAIC)[31] was used to assess best fit to the data and compare among robust JM without competing risks and JM adjusting for competing risk as used in recent JM literature[32]. Being based on pointwise predictive density, WAIC is explicitly linked to cross-validation techniques. The best model should have the lowest values of WAIC, whereby differences of at least 5 points will be considered of importance as follows from recent literature[17,18].

1.5. Dynamic Individual Predictions from Joint Models

1.5.1. Dynamic Predictions from Robust Joint Models with a Single Time-to-Event Outcome

Let $\pi_l(s+u,s)$ denote the subject-specific conditional probability of death (or any other clinical outcome of interest) for a new patient l between landmark time s and future time $s+u$, given that the patient did not experience the event until time point s (so that $T_l^* > s$) and provided patient-specific clinical data collected up to and including time s ($\mathbf{Y}_l = [Y_{l1}, \dots, Y_{lm_l}]', \mathbf{x}_l = [x_{l1}, \dots, x_{lm_l}]', \mathbf{d}_l = [d_{l1}, \dots, d_{lm_l}]', \boldsymbol{c}_l = [c_{l1}, \dots, c_{lr}]'$) and $\boldsymbol{D}_n = \{T_i, E_i, Y_i, t_i;\ i = 1, \dots, n\}$ denotes the sample on which the joint model was fitted (i.e. training dataset). Then,

$$\pi_l(s+u,s) = Pr(s < T_l^* \leq s+u | T_l^* > s, \mathbf{Y}_l, \mathbf{x}_l, \mathbf{d}_l, \mathbf{c}_l, \boldsymbol{D}_n),$$
$$= 1 - Pr(T_l^* > s+u | T_l^* > s, \mathbf{Y}_l, \mathbf{x}_l, \mathbf{d}_l, \mathbf{c}_l, \boldsymbol{D}_n). \quad (16)$$

Recent advancements in dynamic predictions through JM predominantly rely on Bayesian methodologies[16,30,33]. This approach enables the incorporation of parameter uncertainty into the predictions and facilitates the computation of credible intervals. The estimation of $\pi_l(s+u,u)$ is based on the respective posterior predictive distributions, as follows:

$$\pi_l(s+u,s) = \int Pr(T_l^* > s+u | T_l^* > s, \mathbf{Y}_l, \mathbf{x}_l, \mathbf{d}_l, \mathbf{c}_l; \boldsymbol{\Theta}) p\,(\boldsymbol{\Theta} | \boldsymbol{D}_n) d\boldsymbol{\Theta}, \quad (17)$$

where $\boldsymbol{\Theta}$ contains all unknown parameters as delineated in Section 1.2.2. The term $p(\boldsymbol{\Theta}|\boldsymbol{D}_n)$ is the posterior distribution of the parameters given the observed data, which is readily available from the Bayesian inference step after fitting the joint model to the testing dataset. However, the first term in the integrand is more involved and is derived as follows:

$$Pr(T_l^* > s+u | T_l^* > s, \mathbf{Y}_l, \mathbf{x}_l, \mathbf{d}_l, \mathbf{c}_l; \mathbf{\Theta}) =$$

$$= \int Pr(T_l^* > s+u | T_l^* > s, \mathbf{Y}_l, \mathbf{x}_l, \mathbf{d}_l, \mathbf{c}_l, \mathbf{b}_l, V_l, \mathbf{W}_l; \mathbf{\Theta}) p(\mathbf{b}_l, V_l, \mathbf{W}_l | T_l^* > s, \mathbf{Y}_l, \mathbf{x}_l, \mathbf{d}_l, \mathbf{c}_l; \mathbf{\Theta})\, d\mathbf{b}_l dV_l d\mathbf{W}_l$$

$$= \int Pr(T_l^* > s+u | T_l^* > s, \mathbf{c}_l, \mathbf{b}_l, V_l, \mathbf{W}_l; \mathbf{\Theta}) p(\mathbf{b}_l, V_l, \mathbf{W}_l | T_l^* > s, \mathbf{Y}_l, \mathbf{x}_l, \mathbf{d}_l, \mathbf{c}_l; \mathbf{\Theta})\, d\mathbf{b}_l dV_l d\mathbf{W}_l$$

$$= \int \frac{Pr(T_l^* > s+u, | \mathbf{c}_l, \mathbf{b}_l, V_l, \mathbf{W}_l; \mathbf{\Theta})}{Pr(T_l^* > s, | \mathbf{c}_l, \mathbf{b}_l, V_l, \mathbf{W}_l; \mathbf{\Theta})} p(\mathbf{b}_l, V_l, \mathbf{W}_l | T_l^* > s, \mathbf{Y}_l, \mathbf{x}_l, \mathbf{d}_l, \mathbf{c}_l; \mathbf{\Theta})\, d\mathbf{b}_l dV_l d\mathbf{W}_l$$

$$= \int \frac{S(s+u | \mathbf{c}_l, \mathbf{b}_l, V_l, \mathbf{W}_l; \mathbf{\Theta})}{S(s | \mathbf{c}_l, \mathbf{b}_l, V_l, \mathbf{W}_l; \mathbf{\Theta})} p(\mathbf{b}_l, V_l, \mathbf{W}_l | T_l^* > s, \mathbf{Y}_l, \mathbf{x}_l, \mathbf{d}_l, \mathbf{c}_l; \mathbf{\Theta})\, d\mathbf{b}_l dV_l d\mathbf{W}_l\,, \quad (18)$$

where $S(\cdot | \mathbf{c}_l, \mathbf{b}_l, V_l, \mathbf{W}_l; \mathbf{\Theta})$ denotes the conditional survival function for patient l. The transition between lines 2 and 3 above is possible under the assumption of independence between longitudinal and time-to-event outcomes, conditional on the random effects. Yet, another layer of complexity comes from the fact that, for a new patient l, one does not have readily available samples of $p(\mathbf{b}_l, V_l, \mathbf{W}_l | T_l^* > s, \mathbf{Y}_l, \mathbf{x}_l, \mathbf{d}_l, \mathbf{c}_l; \mathbf{\Theta})$. Historically, Rizopoulos (2012)[5] first proposed an MCMC sampling approach in the context of the classical joint model, i.e. $V_l, \mathbf{W}_l$ were not considered, to sample the individual random effects $\mathbf{b}_l$ from a Metropolis-Hastings algorithm with independent proposals from a centred and scaled multivariate t-distribution. That, to this date, remains an integral part of the **JM** package by the same author for generating dynamic individualised predictions[34]. Instead, a newer strategy is adopted here as discussed in recent literature[16,30] and implemented in the **robjm** package . In essence, one can draw a random sample of the individual-specific random variables $\mathbf{b}_l, V_l, \mathbf{W}_l$ from the conditional density $p(\mathbf{b}_l, V_l, \mathbf{W}_l | T_l^* > s, \mathbf{Y}_l, \mathbf{x}_l, \mathbf{d}_l, \mathbf{c}_l; \mathbf{\Theta})$ using:

$$p(\mathbf{b}_l, V_l, \mathbf{W}_l | T_l^* > s, \mathbf{Y}_l, \mathbf{x}_l, \mathbf{d}_l, \mathbf{c}_l; \mathbf{\Theta}) \propto p(\mathbf{Y}_l | \mathbf{b}_l, \mathbf{W}_l, \mathbf{x}_l, \mathbf{d}_l; \mathbf{\Theta}) S(s | \mathbf{b}_l, \mathbf{x}_l, \mathbf{d}_l, \mathbf{c}_l; \mathbf{\Theta}) p(\mathbf{b}_l | V_l; \mathbf{\Theta}) p(V_l | \mathbf{\Theta}) p(\mathbf{W}_l | \mathbf{\Theta})\,, \quad (19)$$

where the last three terms are the priors for $\mathbf{b}_l, V_l, \mathbf{W}_l$. In practice, one uses the MCMC samples for the unknown parameters calculated in the inference step to find $p(\mathbf{\Theta} | \boldsymbol{D}_n)$ after fitting the joint model to the testing dataset to calculate the first two terms in Eq. (19). Essentially, this approach is equivalent to drawing a random sample of $\mathbf{b}_l, V_l, \mathbf{W}_l$ from their joint posterior distribution given the observed data for the new individual and $\mathbf{\Theta}$, which could be directly performed using a purpose-built Stan program corresponding to Eq. (17). Indeed, after sufficient number of warm-up and sampling iterations, one can draw a single sample from the posterior distribution for $\mathbf{b}_l, V_l, \mathbf{W}_l$. The benefits of such an MCMC strategy are two-fold: not only does it allow for a fully Bayesian approach with joint models, for both estimation and prediction steps, but also allows one to leverage the great potential of Stan software in both of these steps. Combining ideas from Eq. (17), (18) and (19), the complete algorithm for calculating $\pi_l(s+u, s)$ is:

(a) draw $\mathbf{\Theta}^{(m)}$ from the MCMC sample of the posterior $p(\mathbf{\Theta} | \boldsymbol{D}_n)$,
(b) draw $\mathbf{b}_l^{(\mathrm{m})}$, $V_l^{(\mathrm{m})}$, $\mathbf{W}_l^{(\mathrm{m})}$ using a purpose-built Stan program corresponding to Eq. (19) as discussed above,
(c) compute $\pi_l^{(m)}\left(s+u, s, \mathbf{b}_l^{(\mathrm{m})}, V_l^{(\mathrm{m})}, \mathbf{W}_l^{(\mathrm{m})}; \mathbf{\Theta}^{(m)}\right) = \frac{S(s+u | \mathbf{c}_l, \mathbf{b}_l^{(\mathrm{m})}, V_l^{(\mathrm{m})}, \mathbf{W}_l^{(\mathrm{m})}; \mathbf{\Theta}^{(m)})}{S(s | \mathbf{c}_l, \mathbf{b}_l^{(\mathrm{m})}, V_l^{(\mathrm{m})}, \mathbf{W}_l^{(\mathrm{m})}; \mathbf{\Theta}^{(m)})}$,

where $m = (1, \dots, M)$ indicates the MCMC sample, selected at random. that is used for each iteration. One repeats this procedure a total of M times and obtains the estimate of $\pi_l(s+u, s)$ as:

$$\hat{\pi}_l(s+u,s) = \frac{1}{M}\sum_{m=1}^{M} \pi_l^{(m)}\left(s+u,s,\mathbf{b}_l^{(\mathrm{m})},V_l^{(\mathrm{m})},\mathbf{W}_l^{(\mathrm{m})};\mathbf{\Theta}^{(m)}\right). \tag{20}$$

As follows from the dynamic nature of the predictions, $\hat{\pi}_l(s+u,s)$ is updated on a future landmark point s' ($s' > s$), to $\hat{\pi}_l(s'+u,s')$, if the patient is still in the risk set at time s' (i.e. $T_l^* > s'$) by taking into account any new data for the longitudinal biomarker from the specific patient recorded in the time interval $(s,s']$. For example, the landmark times s and s' could be subsequent follow-up visits or pre-specified time points of relevance to the patient prognosis.

We developed custom Stan code to sample from $p(\mathbf{b}_l,V_l,\mathbf{W}_l|T_l^* > s,\mathbf{Y}_l,\mathbf{x}_l,\mathbf{d}_l,\mathbf{c}_l;\mathbf{\Theta})$ corresponding to Eq. (19) as well as associated **R** programs to synchronise and combine all sub-steps of the above sampling algorithm for calculating $\hat{\pi}_l(s+u,s)$ for the purposes of this study. That includes another function which acts as a wrapper to the main algorithm to allow for parallelised computation of the dynamic predictions across various combinations of landmark times s and prediction windows u, which helped us minimise computation time for predictions with the testing dataset of the IDEAL trial. Results are obtained with $M = 100$ and presented in the main text.

1.5.2. Dynamic Predictions from Robust Joint Models under Competing Risks

The extension of dynamic predictions for the main clinical outcome of interest in the presence of competing events is more intricate than performing dynamic predictions with a single time-to-event outcome. Hereby, an algorithm to perform dynamic predictions with robust JM under competing risks from Table 1 from main text is presented, to the best of our knowledge, for the first time in literature. It combines elements from prior studies with JM with competing risks, published recently[23,35], and extends them to JM with both competing risk event sub-model structure and robust longitudinal sub-model specification accounting for both *e*- and *b*-outliers.

As before, patient-specific clinical data for a new patient l collected up to and including time s is $\mathbf{Y}_l = [Y_{l1},\dots,Y_{\boldsymbol{l}m_l}]', \mathbf{x}_l = [x_{l1},\dots,x_{\boldsymbol{l}m_l}]', \mathbf{d}_l = [d_{l1},\dots,d_{lm_l}]', \boldsymbol{c}_l = [c_{l1},\dots,c_{lr}]'$) and $\boldsymbol{D}_n = \{T_{ik},E_{ik},Y_i,t_i;\ i = 1,\dots,n\}$ denotes the sample on which the joint model was fitted. As there have been no events observed until time point s (so that $T_{lk}^* > s$) it becomes more pertinent to focus on $\pi_{l,k}(s+u,s)$ as the cumulative incidence being the probability of having an event of type k between landmark time s and future time $s + u$, which allows to account for the competing risks as follows:

$$\pi_{l,k}(s+u,s) = Pr(T_{lk}^* < s+u|\bigcup_{k=1}^{K} T_{lk}^* > s,\mathbf{Y}_l,\mathbf{x}_l,\mathbf{d}_l,\mathbf{c}_l,\boldsymbol{D}_n), \tag{21}$$

which, as follows from the Bayesian estimation approach in Eq. (17) from the previous section, can be expanded based on the corresponding posterior predictive distributions:

$$\pi_{l,k}(s+u,s) = \int Pr(T_{lk}^* < s+u|\bigcup_{k=1}^{K} T_{lk}^* > s,\mathbf{Y}_l,\mathbf{x}_l,\mathbf{d}_l,\mathbf{c}_l;\mathbf{\Theta})p\,(\mathbf{\Theta}|\boldsymbol{D}_n)d\mathbf{\Theta}, \tag{22}$$

where $\mathbf{\Theta}$ is expanded to contain all additional cause-specific parameters from the competing risks sub-model as mentioned in Section 1.3.4 and the term $p(\mathbf{\Theta}|\boldsymbol{D}_n)$ again denotes the posterior distribution of the parameters given the observed data. The first term in the integrand in Eq. (22) can be expanded as:

$$Pr(T_{lk}^* < s + u | \bigcup_{k=1}^{K} T_{lk}^* > s, \mathbf{Y}_l, \mathbf{x}_l, \mathbf{d}_l, \mathbf{c}_{lk}; \mathbf{\Theta}) =$$

$$= \int Pr(T_{lk}^* < s + u | \bigcup_{k=1}^{K} T_{lk}^* > s, \mathbf{Y}_l, \mathbf{x}_l, \mathbf{d}_l, \mathbf{c}_{lk}, \mathbf{b}_l, V_l, \mathbf{W}_l; \mathbf{\Theta}) p(\mathbf{b}_l, V_l, \mathbf{W}_l | T_l^* > s, \mathbf{Y}_l, \mathbf{x}_l, \mathbf{d}_l, \mathbf{c}_{lk}; \mathbf{\Theta})\, d\mathbf{b}_l dV_l d\mathbf{W}_l$$

$$= \int Pr(T_{lk}^* < s + u | \bigcup_{k=1}^{K} T_{lk}^* > s, \mathbf{c}_{lk}, \mathbf{b}_l, V_l, \mathbf{W}_l; \mathbf{\Theta}) p(\mathbf{b}_l, V_l, \mathbf{W}_l | T_l^* > s, \mathbf{Y}_l, \mathbf{x}_l, \mathbf{d}_l, \mathbf{c}_{lk}; \mathbf{\Theta})\, d\mathbf{b}_l dV_l d\mathbf{W}_l$$

$$= \int \frac{Pr\left(T_{lk}^* < s + u, \bigcup_{k=1}^{K} T_{lk}^* > s \middle| \mathbf{c}_{lk}, \mathbf{b}_l, V_l, \mathbf{W}_l; \mathbf{\Theta}\right)}{\Pr(\bigcup_{k=1}^{K} T_{lk}^* > s\,, | \mathbf{c}_{lk}, \mathbf{b}_l, V_l, \mathbf{W}_l; \mathbf{\Theta})} p(\mathbf{b}_l, V_l, \mathbf{W}_l | T_l^* > s, \mathbf{Y}_l, \mathbf{x}_l, \mathbf{d}_l, \mathbf{c}_{lk}; \mathbf{\Theta})\, d\mathbf{b}_l dV_l d\mathbf{W}_l$$

$$= \int \frac{CIF(s + u | \mathbf{c}_{lk}, \mathbf{b}_l, V_l, \mathbf{W}_l; \mathbf{\Theta})}{S(s | \mathbf{c}_{lk}, \mathbf{b}_l, V_l, \mathbf{W}_l; \mathbf{\Theta})} p(\mathbf{b}_l, V_l, \mathbf{W}_l | T_l^* > s, \mathbf{Y}_l, \mathbf{x}_l, \mathbf{d}_l, \mathbf{c}_{lk}; \mathbf{\Theta})\, d\mathbf{b}_l dV_l d\mathbf{W}_l\,, \qquad (23)$$

where $S(\cdot)$ denotes the overall survival function and the cumulative incidence function is $CIF(s, s + u, k) = \int_s^{s+u} h_{lk}(v) S(v) dv$. The transition between lines 2 and 3 above is possible under the assumption of independence between longitudinal and time-to-event outcomes, conditional on the random effects. As in Section 1.4.1, random samples of the individual-specific random variables $\mathbf{b}_l, V_l, \mathbf{W}_l$ from the conditional density $p(\mathbf{b}_l, V_l, \mathbf{W}_l | T_l^* > s, \mathbf{Y}_l, \mathbf{x}_l, \mathbf{d}_l, \mathbf{c}_{lk}; \mathbf{\Theta})$ can be obtained using a purpose-built Stan program to draw from the posterior distribution as suggested from Eq. (19) which is updated to include $S(\cdot)$ as the overall survival function. Thus, the complete algorithm for computing $\pi_{l,k}(s + u, s)$ is:

(a) draw $\mathbf{\Theta}^{(m)}$ from the MCMC sample of the posterior $p(\mathbf{\Theta}|\boldsymbol{D}_n)$,
(b) draw $\mathbf{b}_l^{(\mathrm{m})}$, $V_l^{(\mathrm{m})}$, $\mathbf{W}_l^{(\mathrm{m})}$ using a purpose-built Stan program corresponding to Eq. (19) updated to include $S(\cdot)$ as the overall survival function as discussed above,
(c) compute $\pi_{l,k}^{(m)}\left(s + u, s, \mathbf{b}_l^{(\mathrm{m})}, V_l^{(\mathrm{m})}, \mathbf{W}_l^{(\mathrm{m})}; \mathbf{\Theta}^{(m)}\right) = \frac{CIF(s+u|\mathbf{c}_l, \mathbf{b}_l^{(\mathrm{m})}, V_l^{(\mathrm{m})}, \mathbf{W}_l^{(\mathrm{m})}; \mathbf{\Theta}^{(m)})}{S(s|\mathbf{c}_l, \mathbf{b}_l^{(\mathrm{m})}, V_l^{(\mathrm{m})}, \mathbf{W}_l^{(\mathrm{m})}; \mathbf{\Theta}^{(m)})}$,

where $m = (1, \ldots, M)$ indicates the MCMC sample, selected at random. that is used for each iteration. One repeats this procedure a total of M times and obtains the estimate of $\pi_l(s + u, s)$ as:

$$\hat{\pi}_{l,k}(s + u, s) = \frac{1}{M} \sum_{m=1}^{M} \pi_{l,k}^{(m)}\left(s + u, s, \mathbf{b}_l^{(\mathrm{m})}, V_l^{(\mathrm{m})}, \mathbf{W}_l^{(\mathrm{m})}; \mathbf{\Theta}^{(m)}\right). \qquad (24)$$

Custom Stan program to sample from $p(\mathbf{b}_l, V_l, \mathbf{W}_l | T_l^* > s, \mathbf{Y}_l, \mathbf{x}_l, \mathbf{d}_l, \mathbf{c}_l; \mathbf{\Theta})$ corresponding to Eq. (19) in the case of competing risks as well as associated **R** programs to fully implement the sampling algorithm for calculating $\hat{\pi}_{l,k}(s + u, s)$ were developed for the purposes of this study. That includes an auxiliary function which 'wraps' the main algorithm and facilitates parallelised computation of the dynamic predictions across various combinations of landmark times s and prediction windows u. That substantially decreased computation time for predictions with the testing dataset of the IDEAL trial, whereby results are obtained with $M = 100, K = 3$ as presented in the main text.

2. Innovative Computational Solution

One challenge to the application of joint models is their inherent computational complexity. Therefore, an important consideration for the wider applicability of proposed approaches to large clinical trial datasets is the comparison of computation efficiency with existing software, where available. Currently, there is only one statistical package, the **robjm** package (https://github.com/ozgurasarstat/robjm) in **R**, that allows estimation of the 6 JM specifications with a single time-to-event sub-model (first six models in Table 1 in main text). However, the package does not allow the inclusion of competing risks. Therefore, only models with a survival sub-model ignoring competing risks can be compared. A summary of the comparison is provided in Table S2 and Figure S4 below generated by fitting each model specification 20 times with 6000 iterations (split equally into warm-up and sampling) to the training dataset from the IDEAL trial.

As can be seen in Table S2, the proposed estimation techniques provided similar parameter estimates to the existing **robjm** package (median relative difference in posterior parameter estimates was kept below 8% across all models and parameters, using **robjm** results as reference), however, with superior efficiency. Median estimation times were between 3 and 6 times shorter depending on the model. Looking at coefficients of efficiency, defined as the estimated number of effective samples drawn per unit time for the slowest mixing parameter, reveals an even greater advantage for the proposed estimation procedures, with efficiency between 4.1 and 12.9 times higher than the current state-of-the-art. For completeness, we show a comparison of the sampler geometry, obtained from the median gradient evaluations per iteration across the 20 model fits for each model specification demonstrated in Figure S4 below. The lower the number of gradient evaluations per iteration required, the better the geometry of the sampler, as fewer "leapfrog" steps are taken resulting in faster estimation. We can see that while our computational approach required only 31 to 63 gradient evaluations per iteration, the available package necessitated between 255 and 511. While we used the same priors as in the **robjm** package, the greater efficiency is a result of the combined effect between: i) streamlined Stan model code according to the latest recommendations and ii) use of the newer and faster **cmdstanr** package for sampling, rather than the **rstan** package (internally called within the **robjm** package). These tangible computational gains are promising as to the wider application of the methods considered in various and potentially larger clinical trial datasets, which might render existing JM software simply impractical.

Table S2. Comparison of the computational efficiency between proposed approaches and the **robjm** package. Each model was estimated 20 times with the IDEAL PD training dataset.

Model	Median Relative Parameter Estimates Difference (Range)	Median Time in Minutes (Range)		Median Efficiency (ESS/time)		Median Efficiency Ratio (Range)
		Alternative	robjm	Alternative	robjm	
nor-nor	0.08 (0.006-0.144)	29 (6-31)	122 (109-257)	68.6	11.2	7.2 (5.4-9.8)
t-t-mod1	0.038 (0-0.124)	38 (7-43)	175 (72-565)	50	4.7	9.7 (5.7-34)
t-t-mod2	0.007 (0-0.078)	47 (13-170)	129 (55-419)	3.8	0.8	6.2 (3.2-34.2)
nor-t-mod2	0.054 (0.001-0.11)	22 (9-39)	133 (55-276)	70.2	5.5	12.2 (9.1-38.8)
t-t-mod3	0.012 (0-0.115)	47 (15-87)	137 (65-426)	3.6	0.8	4.1 (2-19.4)
nor-t-mod3	0.069 (0-0.201)	20 (9-43)	113 (70-243)	37.3	2.7	12.9 (8.9-20.9)

ESS: effective sample size; min: minutes.

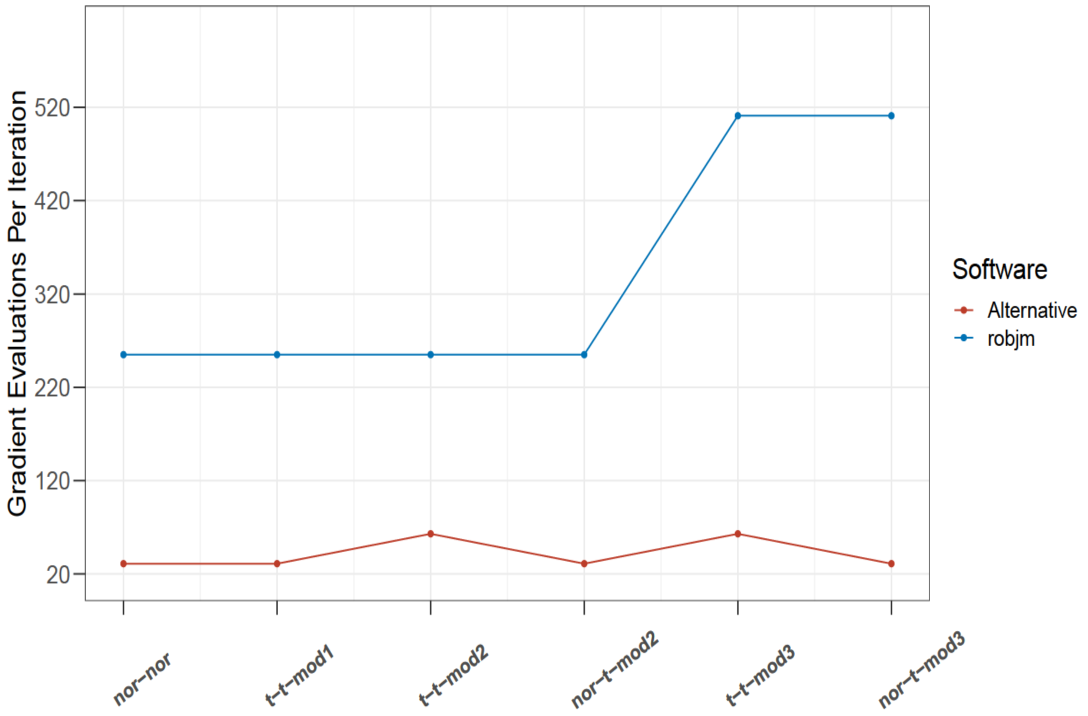

Figure S4. Comparison of median gradient evaluations per iteration required for model estimation between proposed approaches and the **robjm** package. Each model was estimated 20 times with the IDEAL PD training dataset.

3. Results Supplement

3.1. Baseline Characteristics of PD Training and Testing Datasets

Table S3. Baseline patient characteristics of IDEAL trial PD training dataset (N=236) according to event type.

**Characteristic***	**Total (N=236)**	**According to Event Type**				
		**Dead (N=82)**	**Transfer to HD (N=58)**	**KTx (N=35)**	**Censored (N=61)**	***P*-value**
**Age (years)**	61.8 [53.0, 69.6]	67.9 [61.2,72.0]	59.2 [51.7,65.5]	47.8 [41.1,59.1]	61.4 [53.6,69.8]	<.001
**Gender**						0.363
Female	107 (45.3%)	36 (43.9%)	31 (53.4%)	17 (48.6%)	23 (37.7%)	
Male	129 (54.7%)	46 (56.1%)	27 (46.6%)	18 (51.4%)	38 (62.3%)	
**Ethnicity**						0.311
Caucasian	64 (27.1%)	23 (28%)	18 (31%)	5 (14.3%)	18 (29.5%)	
Non-Caucasian	172 (72.9%)	59 (72%)	40 (69%)	30 (85.7%)	43 (70.5%)	
**BMI (kg/m^2)**	27.0 [23.9, 30.7]	26.9 [23.1,30.6]	30.1 [26.4,33.6]	26.9 [23.9,27.8]	25.4 [23.6,28.2]	0.001
**Primary kidney disease**						0.004
Diabetic nephropathy	74 (31.4%)	34 (41.5%)	22 (37.9%)	3 (8.6%)	15 (24.6%)	
Hypertension / Renovascular disease	35 (14.8%)	11 (13.4%)	9 (15.5%)	3 (8.6%)	12 (19.7%)	
Glomerulonephritis	32 (13.6%)	5 (6.1%)	9 (15.5%)	9 (25.7%)	9 (14.8%)	
Polycystic kidney disease	33 (14.0%)	8 (9.8%)	4 (6.9%)	9 (25.7%)	12 (19.7%)	
Other	62 (26.3%)	24 (29.3%)	14 (24.1%)	11 (31.4%)	13 (21.3%)	
**Peritoneal transport status****						0.032
Low	11 (4.7%)	3 (3.7%)	3 (5.2%)	1 (2.9%)	4 (6.6%)	
Low average	75 (4.7%)	16 (19.5%)	25 (43.1%)	15 (42.9%)	19 (31.1%)	
High average	115 (48.7%)	52 (63.4%)	23 (39.7%)	16 (45.7%)	24 (39.3%)	
High	35 (14.8%)	11 (13.4%)	7 (12.1%)	3 (8.6%)	14 (23%)	
**Modality**						0.267
APD	25 (10.6%)	5 (6.1%)	6 (10.3%)	4 (11.4%)	10 (16.4%)	
CAPD	211 (89.4%)	77 (93.9%)	52 (89.7%)	31 (88.6%)	51 (83.6%)	
**Smoking status**						0.088
Never	88 (37.3%)	28 (34.1%)	15 (25.9%)	16 (45.7%)	29 (47.5%)	
Current	22 (9.3%)	10 (12.2%)	3 (5.2%)	4 (11.4%)	5 (8.2%)	
Former	126 (53.4%)	44 (53.7%)	40 (69%)	15 (42.9%)	27 (44.3%)	
**Start of dialysis**						0.604
Early Start	129 (54.7%)	47 (57.3%)	34 (58.6%)	16 (45.7%)	32 (52.5%)	
Late Start	107 (45.3%)	35 (42.7%)	24 (41.4%)	19 (54.3%)	29 (47.5%)	
**Initial dialysis dose**						0.299
Full	205 (86.9%)	69 (84.1%)	52 (89.7%)	28 (80%)	56 (91.8%)	
Incremental	31 (13.1%)	13 (15.9%)	6 (10.3%)	7 (20%)	5 (8.2%)	
**Peritonitis rate (episodes/year)**	0.3 [0.0, 0.9]	0.4 [0.0,1.1]	0.7 [0.2,1.2]	0.0 [0.0,0.4]	0.2 [0.0,0.6]	<.001
**Comorbidities**						
Diabetes mellitus	94 (39.8%)	47 (57.3%)	24 (41.4%)	3 (8.6%)	20 (32.8%)	<.001
Hypertension	225 (95.3%)	78 (95.1%)	54 (93.1%)	33 (94.3%)	60 (98.4%)	0.572
CVD	78 (33.1%)	43 (52.4%)	17 (29.3%)	4 (11.4%)	14 (23%)	<.001
Hyperlipidaemia	143 (60.6%)	51 (62.2%)	39 (67.2%)	17 (48.6%)	36 (59%)	0.342

Table continued on next page

Table S3 (continued). Baseline patient characteristics of IDEAL trial PD training dataset (N=236) according to event type.

Characteristic*	Total (N=236)	According to Event Type				
		Dead (N=82)	Transfer to HD (N=58)	KTx (N=35)	Censored (N=61)	*P*-value
Stoke/Davies Score	1.0 [0.0, 1.0]	1.0 [1.0,2.0]	1.0 [0.0,1.0]	0.0 [0.0,0.0]	1.0 [0.0,1.0]	<.001
**Laboratory parameters**						
Albumin (g/L)	38.0 [34.8, 41.0]	35.0 [30.0,39.0]	38.0 [35.0,42.0]	41.0 [38.5,42.0]	39.0 [37.0,41.0]	<.001
Creatinine (mmol/L)	9.9 [8.2, 11.7]	9.2 [7.5,10.6]	10.3 [9.0,11.9]	10.6 [8.9,12.7]	10.3 [8.1,12.8]	<.001
Urea (mmol/L)	5.3 [4.4, 6.2]	5.5 [4.4,6.2]	5.3 [4.4,6.0]	5.2 [4.2,5.9]	5.4 [4.4,6.4]	0.844
eGFR (mL/min/1.73m^2)	11.6 [10.1, 13.2]	11.9 [10.3,14.2]	11.5 [10.1,12.9]	12.2 [10.4,13.4]	10.9 [9.6,12.6]	0.093
Haemoglobin (g/L)	112.0 [102.0, 121.0]	111.0 [98.0,120.0]	110.5 [102.0,121.0]	115.0 [111.5,122.0]	110.0 [104.0,123.0]	0.068
White blood cell (x10^^{9}/L)	7.3 [6.2, 8.6]	7.5 [6.2,8.8]	7.8 [6.5,8.6]	7.2 [5.8,8.1]	7.1 [5.8,8.7]	0.488
Calcium (mmol/L)	2.3 [2.2, 2.4]	2.3 [2.2,2.4]	2.3 [2.2,2.4]	2.3 [2.2,2.4]	2.4 [2.2,2.5]	0.037
Phosphorus (mmol/L)	1.8 [1.5, 2.1]	1.8 [1.5,2.1]	1.8 [1.6,2.2]	1.7 [1.5,1.9]	1.7 [1.6,2.1]	0.765
Cholesterol (mmol/L)	4.4 [3.8, 5.3]	4.3 [3.5,5.5]	4.5 [3.9,5.5]	4.5 [3.8,5.2]	4.4 [3.9,5.1]	0.687
Triglycerides (mmol/L)	1.9 [1.3, 2.9]	1.7 [1.2,2.6]	2.2 [1.4,3.2]	1.9 [1.2,2.5]	1.7 [1.3,2.4]	0.335
Intact parathyroid hormone (pmol/L)***	27.5 [14.4, 60.1]	25.0 [15.2,45.1]	37.9 [19.7,76.0]	20.3 [8.6,56.3]	33.8 [11.1,62.0]	0.104
**Medications**						
Angiotensin converting-enzyme inhibitor	90 (38.1%)	30 (36.6%)	25 (43.1%)	9 (25.7%)	25 (41%)	0.364
Angiotensin II receptor blocker	39 (16.5%)	9 (11%)	11 (19%)	6 (17.1%)	13 (21.3%)	0.376
Statin	131 (55.5%)	49 (59.8%)	35 (60.3%)	14 (40%)	33 (54.1%)	0.203
Erythropoiesis stimulating agent	61 (25.8%)	32 (39%)	10 (17.2%)	5 (14.3%)	14 (23%)	0.006

APD: automated peritoneal dialysis; BMI: body mass index; CAPD: continuous ambulatory peritoneal dialysis; CVD: cardiovascular disease (defined as a composite of ischemic heart disease, congestive heart failure and peripheral vascular disease); eGFR: estimated glomerular filtration rate (using the Cockcroft-Gault equation); HD: hemodialysis; KTx: kidney transplantation

* Continuous non-normally distributed variables (according to Shapiro-Wilk test) were expressed as median (interquartile range) and categorical variables were presented as frequencies (percentages). Comparison of continuous variables according to event type was performed using Kruskal-Wallis rank sum test (or Mann-Whitney test for two-group comparison) and comparison of categorical variables was accomplished using the chi-squared test.

** Calculated after imputation of baseline data for 80 patients (33.9%) using the nearest K neighbours method with the **simputation** R package.

*** Calculated after imputation of baseline data for 29 patients (12.3%) using the nearest K neighbours method with the **simputation** R package.

Table S4. Baseline patient characteristics of IDEAL trial PD testing dataset (N=78) according to event type.

Characteristic*	Total (N=78)	According to Event Type				*P*-value
		Dead (N=32)	Transfer to HD (N=17)	KTx (N=10)	Censored (N=19)	
**Age (years)**	63.6 [55.2, 73.6]	62.7 [56.9,71.2]	44.0 [38.4,55.4]	63.5 [56.3,75.7]	68.3 [56.6,74.1]	.002
**Gender**						0.846
Female	23 (29.5%)	9 (28.1%)	4 (23.5%)	3 (30%)	7 (36.8%)	
Male	55 (70.5%)	23 (71.9%)	13 (76.5%)	7 (70%)	12 (63.2%)	
**Ethnicity**						0.057
Caucasian	54 (69.2%)	19 (59.4%)	15 (88.2%)	9 (90%)	11 (57.9%)	
Non-Caucasian	24 (30.8%)	13 (40.6%)	2 (11.8%)	1 (10%)	8 (42.1%)	
**BMI (kg/m^2)**	27.4 [25.3, 29.9]	27.5 [25.6,30.1]	27.7 [26.7,31.0]	27.2 [24.7,29.4]	27.3 [25.2,29.3]	0.830
**Primary kidney disease**						0.098
Diabetic nephropathy	28 (35.9%)	5 (17.9%)	17 (60.7%)	5 (17.9%)	1 (3.6%)	
Hypertension / Renovascular disease	11 (14.1%)	3 (27.3%)	5 (45.5%)	3 (27.3%)	0 (0.0%)	
Glomerulonephritis	13 (16.7%)	3 (23.1%)	4 (30.8%)	4 (30.8%)	2 (15.4%)	
Polycystic kidney disease	6 (7.7%)	2 (33.3%)	0 (0.0%)	1 (16.7%)	3 (50.0%)	
Other	20 (25.6%)	6 (30.0%)	6 (30.0%)	4 (20.0%)	4 (20.0%)	
**Peritoneal transport status****						0.679
Low	1 (1.3%)	1 (3.1%)	0 (0%)	0 (0%)	0 (0%)	
Low average	24 (30.8%))	10 (31.2%)	5 (29.4%)	2 (20%)	7 (36.8%)	
High average	42 (53.8%))	19 (59.4%)	8 (47.1%)	7 (70%)	8 (42.1%)	
High	11 (16.1%)	2 (6.2%)	4 (23.5%)	1 (10%)	4 (21.1%)	
**Modality**						0.995
APD	9 (11.5%)	4 (12.5%)	2 (11.8%)	1 (10%)	2 (10.5%)	
CAPD	69 (88.5%)	28 (87.5%)	15 (88.2%)	9 (90%)	17 (89.5%)	
**Smoking status**						0.857
Never	38 (48.7%)	13 (40.6%)	9 (52.9%)	5 (50%)	11 (57.9%)	
Current	6 (7.7%)	2 (6.2%)	1 (5.9%)	1 (10%)	2 (10.5%)	
Former	34 (43.6%)	17 (53.1%)	7 (41.2%)	4 (40%)	6 (31.6%)	
**Start of dialysis**						0.923
Early Start	38 (48.7%)	16 (50%)	9 (52.9%)	5 (50%)	8 (42.1%)	
Late Start	40 (51.3%)	16 (50%)	8 (47.1%)	5 (50%)	11 (57.9%)	
**Initial dialysis dose**						0.534
Full	71 (91.0%)	30 (93.8%)	14 (82.4%)	9 (90%)	18 (94.7%)	
Incremental	7 (9.0%)	2 (6.2%)	3 (17.6%)	1 (10%)	1 (5.3%)	
**Peritonitis rate (episodes/year)**	0.2 [0.0, 0.9]	0.6 [0.0,1.2]	0.0 [0.0,0.0]	0.0 [0.0,0.4]	0.5 [0.1,1.1]	0.005
**Comorbidities**						
Diabetes mellitus	33 (42.3%)	21 (65.6%)	5 (29.4%)	1 (10%)	6 (31.6%)	0.004
Hypertension	73 (93.6%)	29 (90.6%)	17 (100%)	8 (80%)	19 (100%)	0.111
CVD	26 (33.3%)	13 (40.6%)	6 (35.3%)	1 (10%)	6 (31.6%)	0.352
Hyperlipidaemia	45 (57.7%)	19 (59.4%)	11 (64.7%)	2 (20%)	13 (68.4%)	0.069

Table continued on next page

Table S4 (continued). Baseline patient characteristics of IDEAL trial PD testing dataset (N=78) according to event type.

Characteristic*	Total (N=236)	According to Event Type				
		Dead (N=82)	Transfer to HD (N=58)	KTx (N=35)	Censored (N=61)	*P*-value
Stoke/Davies Score	1.0 [0.0, 1.0]	1.0 [0.0,1.0]	0.0 [0.0,0.0]	0.0 [0.0,1.5]	1.0 [1.0,2.0]	0.017
**Laboratory parameters**						
Albumin (g/L)	36.0 [32.0, 39.0]	34.0 [30.0,36.0]	38.5 [37.0,40.0]	36.0 [32.5,42.0]	36.0 [31.0,39.0]	0.048
Creatinine (mmol/L)	9.7 [8.1, 11.0]	10.1 [8.6,12.6]	10.5 [9.0,10.8]	8.5 [7.6,10.3]	10.0 [8.3,11.2]	.455
Urea (mmol/L)	5.2 [4.1, 6.5]	4.7 [4.1,5.9]	5.3 [4.8,5.8]	4.7 [3.7,6.3]	5.9 [4.7,7.0]	.178
eGFR (mL/min/1.73m^2)	11.7 [10.2, 13.6]	11.2 [9.9,13.2]	13.8 [11.9,18.4]	12.3 [10.5,15.1]	11.3 [10.3,12.6]	.069
Haemoglobin (g/L)	112.0 [102.0, 119.8]	103.0 [100.0,119.0]	113.0 [104.0,116.0]	112.0 [106.5,121.0]	112.0 [101.5,119.5]	.841
White blood cell (x10^^{9}/L)	6.8 [6.0, 8.4]	6.8 [6.0,8.4]	6.8 [6.5,7.5]	6.7 [5.9,8.6]	7.1 [6.1,8.6]	.985
Calcium (mmol/L)	2.3 [2.2, 2.4]	2.3 [2.1,2.5]	2.3 [2.1,2.3]	2.4 [2.2,2.5]	2.2 [2.2,2.4]	.616
Phosphorus (mmol/L)	1.7 [1.5, 2.0]	1.6 [1.5,2.0]	1.8 [1.6,2.0]	1.7 [1.4,2.0]	1.7 [1.4,2.0]	.851
Cholesterol (mmol/L)	4.3 [3.5, 5.3]	4.6 [4.0,5.2]	4.6 [4.1,5.5]	4.7 [3.5,5.4]	3.8 [3.4,4.5]	.194
Triglycerides (mmol/L)	1.6 [1.1, 2.3]	1.5 [1.2,2.4]	1.9 [1.5,3.9]	1.3 [1.0,1.6]	1.7 [0.9,2.4]	.287
Intact parathyroid hormone (pmol/L)***	26.0 [7.1, 38.0]	18.3 [2.5,26.7]	41.5 [28.0,47.8]	26.0 [15.3,36.9]	25.0 [7.4,35.6]	.048
**Medications**						
Angiotensin converting-enzyme inhibitor	31 (39.7%)	11 (34.4%)	7 (41.2%)	4 (40%)	9 (47.4%)	0.835
Angiotensin II receptor blocker	15 (19.2%)	6 (18.8%)	4 (23.5%)	2 (20%)	3 (15.8%)	0.949
Statin	45 (57.7%)	16 (50%)	10 (58.8%)	5 (50%)	14 (73.7%)	0.389
Erythropoiesis stimulating agent	14 (17.9%)	8 (25%)	4 (23.5%)	0 (0%)	2 (10.5%)	0.227

APD: automated peritoneal dialysis; BMI: body mass index; CAPD: continuous ambulatory peritoneal dialysis; CVD: cardiovascular disease (defined as a composite of ischemic heart disease, congestive heart failure and peripheral vascular disease); eGFR: estimated glomerular filtration rate (using the Cockcroft-Gault equation); HD: hemodialysis; KTx: kidney transplantation

* Continuous non-normally distributed variables (according to Shapiro-Wilk test) were expressed as median (interquartile range) and categorical variables were presented as frequencies (percentages). Comparison of continuous variables according to event type was performed using Kruskal-Wallis rank sum test (or Mann-Whitney test for two-group comparison) and comparison of categorical variables was accomplished using the chi-squared test.

** Calculated after imputation of baseline data for 24 patients (30.8%) using the nearest K neighbours method with the **simputation** R package.

*** Calculated after imputation of baseline data for 10 patients (12.8%) using the nearest K neighbours method with the **simputation** R package.

3.2. Selecting Association Structure for JM

An essential step in constructing JM involves the selection of the association between albumin trajectory and the survival sub-model. An initial JM consisted of the linear mixed effects model for albumin and a survival sub-model under a parametric baseline hazard under the Weibull PH specification as introduced in Section 1.3.1. The longitudinal and survival sub-models included all of the baseline covariates obtained from covariate selection procedure (right panels of Figure S3). Altogether, six versions of this initial JM were estimated with the IDEAL trial PD training dataset to help select the association structure between the two sub-models, whereby association structures were provided in Table S1. The model selection results in

terms of the WAIC values and rank according to WAIC are presented in Table S5 below. A difference of at least 5 points in WAIC value is considered of importance. One can observe that the current value link function (also referred to as '*nor-nor*' model) has the best fit to the IDEAL dataset, which is the basis for robust JM with and without competing risks for the rest of this study. Of note, the current value link outperforms any other alternative association structure, including the 'Current value + Current slope' link, which suggests that the addition of temporaneous rate of change in addition to current value of albumin does not have much contribution to explain the risk of death in PD patients as found the IDEAL PD training dataset. All JM considered in this work, will thus assume a 'current value' link between longitudinal albumin and the occurrence of all-cause death, transfer to HD or KTx.

We note that in the selected 'Current value' JM model as the basic framework we built upom there is no term for the earlier (baseline) albumin level or for the slope of decline. The coefficient is therefore log-linear: every 1 g/L lower current albumin is associated with the same proportional increase in instantaneous mortality risk, whether the drop is from 41 to 40 g/L or from 30 to 29 g/L. To verify this assumption, we fitted an interaction between current and baseline albumin; which did not improve model fit (ΔWAIC < 2).

Table S5. Comparison of JM fits with various alternative link structures between albumin and survival sub-models.

Model	WAIC	Rank	Estimation time (min)
Current value	12518.14	1	5.8
Current slope*	12582.96	4	10.5
Current value + Current slope*	12522.36	2	9.2
Cumulative effects (area under the curve of the linear predictor)*	12591.87	5	59.2
Shared random effects (b_0)**	13737.49	6	4.2
Shared random effects (b_0, b_1)**	12542.25	3	2.3

* Estimated with '*stan_jm*' function as part of **rstanarm** package in **R**.

** Estimated with a purpose-built program for the purposes of this sutdy which extends the '*stan_jm*' function of the **rstanarm** package in **R**.

3.3. Checking the Appropriateness of Weibull PH Parametric Model for All-Cause Mortality Baseline Hazard

The survival sub-model is diagnosed in light of the suitability of Weibull PH parametric model for all-cause mortality in incident PD patients. A plot of the scaled total-time-on-test (TTT) transform of survival times in panel A of Figure S5 below suggests a monotonically increasing hazard function for all-cause mortality of patients from the IDEAL PD training dataset. Thus, the Weibull distribution is a good candidate as it is widely used parametric model for monotonically increasing or decreasing survival baseline hazard. Furthermore, panel B of Figure S5 displays a plot the Kaplan-Meier estimate of the residuals from a Weibull proportional hazards model (without any covariates) fitted to the training dataset in red colour superimposed over the non-parametric Kaplan-Meier estimate of survival (in black). The closeness of the red and black curves visually demonstrates that the Weibull PH baseline hazard is a good match for the data. As the final specification of the survival sub-model of the JM includes various baseline risk factors (as summarised in Figure S3 above), a test for proportionality of hazards is performed using the *cox.zph*() function in **R** with corresponding results for each covariate to be included in the survival model as well as a global test are presented in panel C of Figure S5. No deviation of the proportionality of hazards assumption can be diagnosed for the selected baseline risk factors at any reasonable level of significance as follows from the high p-values. As a final check, panel D of Figure S5 displays the estimated cumulative hazard vs Cox-Snell residuals calculated from the survival sub-model of the '*nor-nor*' model. The plot is generated by drawing a random sample of size 200 from the posterior simulations for the fitted joint model, and the estimated cumulative hazard and Cox-Snell residuals are computed for each sample. The plot should be roughly a straight line with unit slope when the model is adequate. The MCMC sample means of the residuals (black dots) show little deviation from a straight line with unit slope, suggesting that the Weibull PH model with both longitudinal albumin and remaining baseline risk factors is a good fit for the survival data from the IDEAL PD training dataset. Cause of death for patients in the IDEAL trial PD training and testing datasets is given in Table S6 below.

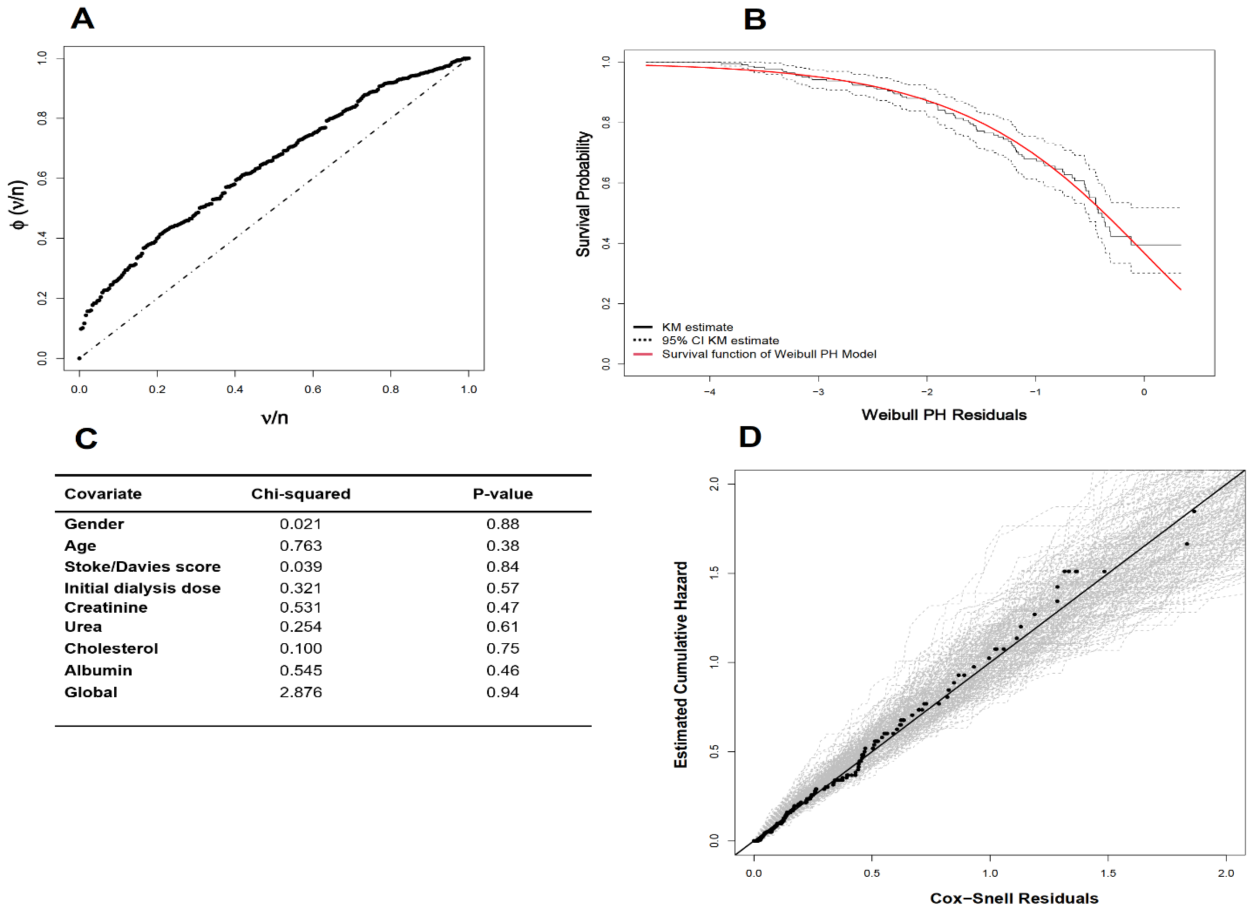

Figure S5 Visual inspection of suitability of Weibull PH parametric model for survival times in the IDEAL PD training dataset. A: Plot of the scaled TTT for detection of the shape of the hazard function. B: Plot of the Kaplan-Meier estimate of the residuals from a fitted Weibull PH model without covariates to survival data from IDEAL PD training dataset (red line) superimposed over the Kaplan-Meier estimate of the survival function. C: Results from proportionality of hazards test for each covariate as well as a global test for the survival model as a whole with the *cox.zph* function in **R**. D: Plot of the estimated cumulative hazard versus Cox-Snell residuals from the initial JM '*nor-nor*'.

Table S6. Cause of death for PD patients from IDEAL trial PD training and testing datasets.

**Cause**	**IDEAL Training Dataset (N = 236, 82 deaths)**	**IDEAL Testing Dataset (N = 78, 32 deaths)**
Cardiovascular diseases	41 (50.0%)	15 (46.9%)
Cerebrovascular disease	2 (2.4%)	0
Digestive tract disease	0	1 (3.1%)
Other causes	12 (14.6%)	4 (12.5%)
Other infectious diseases	14 (17.1%)	4 (12.5%)
Peritonitis	6 (7.3%)	7 (21.9%)
Tumour	6 (7.3%)	1 (3.1%)
Unknown	1 (1.2%)	0

3.4. Parameter Estimates and Estimation Properties of JM Fitted to the PD Training Dataset

Table S7. Posterior summaries of the joint model parameters fitted to the IDEAL trial PD training dataset ignoring competing risks.

Parameter	nor-nor<br>Mean (95% CI) | P-value*	t-t-mod1<br>Mean (95% CI) | P-value*	t-t-mod2<br>Mean (95% CI) | P-value*	nor-t-mod2<br>Mean (95% CI) | P-value*	t-t-mod3<br>Mean (95% CI) | P-value*	nor-t-mod3<br>Mean (95% CI) | P-value*
**Albumin sub-model**						
Intercept	21.20, (14.42, 27.96) | <0.001	24.47, (17.90, 30.95) | <0.001	24.81, (17.96, 31.83) | <0.001	21.36, (14.51, 28.33) | <0.001	24.59, (17.66, 31.40) | <0.001	20.53, (13.75, 27.40) | <0.001
Time on PD (years)	-0.96, (-1.30, -0.64) | <0.001	-0.67, (-0.97, -0.39) | <0.001	-0.78, (-1.08, -0.48) | <0.001	-0.85, (-1.15, -0.55) | <0.001	-0.68, (-0.96, -0.40) | <0.001	-0.76, (-1.05, -0.47) | <0.001
Gender (Male)	1.18, (0.11, 2.30) | 0.033	0.95, (-0.07, 1.98) | 0.065	0.97, (-0.06, 2.01) | 0.065	1.08, (0.00, 2.14) | 0.050	0.91, (-0.08, 1.89) | 0.074	0.98, (-0.14, 2.06) | 0.084
BMI (kg/m^2)**	0.11, (0.00, 0.21) | 0.047	0.08, (-0.02, 0.18) | 0.121	0.08, (-0.02, 0.19) | 0.106	0.10, (-0.01, 0.21) | 0.066	0.08, (-0.01, 0.18) | 0.089	0.10, (-0.00, 0.21) | 0.060
Stoke/Davies Score**	-1.33, (-1.90, -0.76) | <0.001	-1.44, (-1.94, -0.93) | <0.001	-1.39, (-1.95, -0.82) | <0.001	-1.30, (-1.88, -0.74) | <0.001	-1.46, (-1.99, -0.90) | <0.001	-1.33, (-1.92, -0.74) | <0.001
Calcium (mmol/L)**	2.25, (0.06, 4.39) | 0.042	1.93, (-0.13, 4.01) | 0.067	1.47, (-0.49, 3.51) | 0.148	2.26, (0.03, 4.43) | 0.046	1.70, (-0.39, 3.80) | 0.113	2.53, (0.27, 4.67) | 0.027
Creatinine (mg/dL)**	0.24, (0.05, 0.43) | 0.009	0.18, (0.01, 0.35) | 0.037	0.20, (0.03, 0.37) | 0.023	0.25, (0.07, 0.43) | 0.007	0.20, (0.02, 0.38) | 0.030	0.26, (0.08, 0.45) | 0.007
Hemoglobin (g/L)**	0.05, (0.01, 0.08) | 0.005	0.04, (0.01, 0.07) | 0.008	0.04, (0.01, 0.07) | 0.006	0.05, (0.01, 0.08) | 0.004	0.04, (0.01, 0.07) | 0.002	0.05, (0.01, 0.08) | 0.004
$\Sigma(1,1)$	13.81, (10.80, 17.35) | <0.001	10.62, (8.07, 13.68) | <0.001	8.81, (5.80, 12.39) | <0.001	13.13, (10.16, 16.85) | <0.001	8.53, (5.75, 12.19) | <0.001	13.92, (10.94, 17.50) | <0.001
$\Sigma(1,2)$	-1.10, (-2.51, 0.19) | 0.098	-0.65, (-1.77, 0.39) | 0.226	-0.53, (-1.65, 0.42) | 0.282	-0.85, (-2.28, 0.40) | 0.216	-0.25, (-1.22, 0.58) | 0.599	-0.49, (-1.86, 0.73) | 0.480
$\Sigma(2,2)$	2.86, (1.92, 4.01) | <0.001	2.19, (1.46, 3.13) | <0.001	1.96, (1.19, 3.00) | <0.001	2.40, (1.53, 3.50) | <0.001	1.74, (1.06, 2.63) | <0.001	2.20, (1.41, 3.18) | <0.001
γ***	—	6.13, (4.64, 8.00) | <0.001	—	—	—	—
ϕ***	—	—	5.83, (2.82, 13.31) | <0.001	—	4.91, (2.49, 10.83) | <0.001	—
σ	3.53, (3.42, 3.66) | <0.001	2.94, (2.77, 3.11) | <0.001	2.87, (2.69, 3.05) | <0.001	2.88, (2.71, 3.06) | <0.001	2.46, (2.30, 2.62) | <0.001	2.48, (2.32, 2.64) | <0.001
δ***	—	—	5.54, (4.16, 7.28) | <0.001	5.36, (4.04, 7.04) | <0.001	3.64, (3.01, 4.41) | <0.001	3.61, (3.02, 4.37) | <0.001
**Survival sub-model hazard ratios**						
Gender (Male)	1.91, (1.09, 3.35) | 0.023	1.87, (1.08, 3.34) | 0.026	1.78, (1.05, 3.17) | 0.029	1.83, (1.06, 3.19) | 0.028	1.58, (0.95, 2.74) | 0.077	1.64, (0.97, 2.86) | 0.067
Age**	1.03, (1.00, 1.05) | 0.036	1.03, (1.00, 1.06) | 0.042	1.03, (1.00, 1.06) | 0.047	1.03, (1.00, 1.06) | 0.043	1.03, (1.00, 1.06) | 0.019	1.03, (1.00, 1.06) | 0.020
Stoke/Davies Score **	1.15, (0.90, 1.47) | 0.257	1.17, (0.92, 1.51) | 0.209	1.18, (0.93, 1.52) | 0.177	1.16, (0.91, 1.47) | 0.237	1.19, (0.94, 1.53) | 0.154	1.18, (0.92, 1.50) | 0.188
Initial dialysis dose** (Incremental vs Full)	3.86, (1.89, 7.46) | <0.001	3.89, (1.92, 7.76) | <0.001	3.52, (1.66, 7.07) | <0.001	3.93, (1.97, 7.55) | <0.001	3.49, (1.77, 6.75) | <0.001	3.88, (1.90, 7.49) | <0.001
Creatinine (mg/dL)**	0.84, (0.74, 0.94) | 0.003	0.84, (0.74, 0.94) | 0.003	0.84, (0.74, 0.94) | 0.005	0.84, (0.75, 0.94) | 0.001	0.85, (0.75, 0.94) | 0.003	0.84, (0.75, 0.95) | 0.004
Urea (g/L)**	1.30, (1.10, 1.53) | 0.003	1.31, (1.11, 1.55) | 0.003	1.29, (1.09, 1.52) | 0.002	1.32, (1.12, 1.57) | 0.001	1.29, (1.10, 1.53) | 0.005	1.33, (1.12, 1.57) | 0.001
Cholesterol(mmol/L)**	1.30, (1.07, 1.53) | 0.008	1.30, (1.07, 1.54) | 0.007	1.29, (1.08, 1.52) | 0.009	1.28, (1.06, 1.52) | 0.011	1.28, (1.07, 1.51) | 0.007	1.28, (1.07, 1.52) | 0.009
Albumin (g/L)****	1.27, (1.20, 1.36) | <0.001	1.27, (1.20, 1.35) | <0.001	1.25, (1.19, 1.34) | <0.001	1.28, (1.21, 1.37) | <0.001	1.22, (1.16, 1.29) | <0.001	1.24, (1.17, 1.33) | <0.001
Weibull shape	1.63, (1.34, 1.94) | <0.001	1.65, (1.36, 1.96) | <0.001	1.66, (1.37, 1.98) | <0.001	1.65, (1.36, 1.95) | <0.001	1.66, (1.37, 1.97) | <0.001	1.67, (1.38, 1.98) | <0.001

BMI: Body mass index; CI: credible interval; PD: peritoneal dialysis; Σ variance-covariance parameters of random effects.

* Posterior means are given along with 95% credible intervals. P-values were calculated based on the posterior distribution tail probabilities for containing the zero value.

** Values taken at baseline.

*** γ denotes the degrees of freedom parameter from *t*-distribution for both random and residual error applicable to '*t-t-mod1*' only; ϕ and δ denote the degrees of freedom parameter for *t*-distributed random effects for random effects and residual error, respectively.

**** Hazard ratio per 1 g/L decrease in albumin is showed.

Table S8. Posterior summaries of the joint model parameters fitted to the IDEAL PD training dataset accounting for outliers and competing risks.

Parameter	nor-nor-cr	t-t-mod1-cr	t-t_mod2-cr	nor-t-mod2-cr	t-t-mod3-cr	nor-t-mod3-cr
	Mean (95% CI) | P-value*	Mean (95% CI) | P-value*	Mean (95% CI) | P-value*	Mean (95% CI) | P-value*	Mean (95% CI) | P-value*	Mean (95% CI) | P-value*
**Albumin sub-model**						
Intercept	21.16, (14.38, 28.03) | <0.001	24.45, (17.76, 30.94) | <0.001	24.78, (17.51, 31.59) | <0.001	21.39, (14.42, 28.16) | <0.001	24.82, (17.51, 31.80) | <0.001	20.68, (13.84, 27.58) | <0.001
Time on PD	-0.96, (-1.28, -0.64) | <0.001	-0.66, (-0.95, -0.38) | <0.001	-0.78, (-1.08, -0.49) | <0.001	-0.84, (-1.15, -0.55) | <0.001	-0.67, (-0.99, -0.39) | <0.001	-0.75, (-1.04, -0.46) | <0.001
Gender (Male)	1.15, (0.07, 2.23) | 0.038	0.97, (-0.05, 1.98) | 0.062	0.96, (-0.05, 1.99) | 0.061	1.09, (0.02, 2.15) | 0.046	0.88, (-0.10, 1.94) | 0.082	0.98, (-0.13, 2.12) | 0.082
BMI (kg/m^2)**	0.11, (-0.00, 0.21) | 0.057	0.08, (-0.02, 0.18) | 0.125	0.08, (-0.02, 0.19) | 0.126	0.10, (-0.00, 0.20) | 0.056	0.08, (-0.02, 0.19) | 0.134	0.10, (-0.00, 0.21) | 0.061
Stoke/Davies Score**	-1.34, (-1.92, -0.76) | <0.001	-1.44, (-1.96, -0.92) | <0.001	-1.39, (-1.97, -0.81) | <0.001	-1.30, (-1.88, -0.74) | <0.001	-1.46, (-2.00, -0.91) | <0.001	-1.34, (-1.90, -0.78) | <0.001
Calcium (mmol/L)**	2.33, (0.18, 4.49) | 0.034	1.94, (-0.11, 4.08) | 0.062	1.51, (-0.51, 3.53) | 0.136	2.32, (0.15, 4.60) | 0.037	1.60, (-0.33, 3.66) | 0.095	2.57, (0.42, 4.78) | 0.020
Creatinine (mg/dL)**	0.24, (0.06, 0.42) | 0.012	0.18, (0.01, 0.35) | 0.038	0.20, (0.02, 0.38) | 0.029	0.24, (0.06, 0.43) | 0.008	0.20, (0.03, 0.40) | 0.023	0.26, (0.07, 0.44) | 0.008
Hemoglobin (g/L)**	0.05, (0.01, 0.08) | 0.004	0.04, (0.01, 0.07) | 0.004	0.04, (0.01, 0.07) | 0.004	0.05, (0.01, 0.08) | 0.009	0.04, (0.01, 0.07) | 0.025	0.05, (0.01, 0.08) | 0.004
Σ(1,1)	13.66, (10.67, 17.21) | <0.001	10.57, (8.05, 13.55) | <0.001	8.71, (5.77, 12.25) | <0.001	13.03, (10.12, 16.52) | <0.001	8.51, (5.30, 12.25) | <0.001	13.79, (10.86, 17.27) | <0.001
Σ(1,2)	-0.99, (-2.48, 0.40) | 0.156	-0.62, (-1.82, 0.43) | 0.273	-0.47, (-1.57, 0.44) | 0.344	-0.74, (-2.26, 0.51) | 0.282	-0.22, (-1.20, 0.60) | 0.708	-0.41, (-1.74, 0.81) | 0.538
Σ(2,2)	2.86, (1.91, 4.05) | <0.001	2.19, (1.45, 3.20) | <0.001	1.95, (1.19, 2.95) | <0.001	2.37, (1.50, 3.55) | <0.001	1.71, (1.03, 2.66) | <0.001	2.16, (1.38, 3.18) | <0.001
γ***	—	6.16, (4.63, 8.04) | <0.001	—	—	—	—
ϕ***	—	—	5.83, (2.86, 13.99) | <0.001	—	5.13, (2.25, 11.17) | <0.001	—
Σ	3.54, (3.42, 3.66) | <0.001	2.95, (2.78, 3.11) | <0.001	2.86, (2.69, 3.03) | <0.001	2.88, (2.71, 3.05) | <0.001	2.46, (2.31, 2.62) | <0.001	2.47, (2.32, 2.64) | <0.001
δ***	—	—	5.48, (4.11, 7.20) | <0.001	5.37, (4.06, 7.05) | <0.001	3.63, (3.04, 4.41) | <0.001	3.59, (3.03, 4.35) | <0.001
**Survival sub-model hazard ratios**						
Gender (Male)	1.91, (1.09, 3.36) | 0.021	1.86, (1.05, 3.33) | 0.034	1.79, (1.05, 3.10) | 0.032	1.82, (1.06, 3.22) | 0.026	1.60, (0.95, 2.74) | 0.075	1.63, (0.95, 2.83) | 0.076
Age**	1.03, (1.00, 1.06) | 0.035	1.03, (1.00, 1.06) | 0.040	1.03, (1.00, 1.06) | 0.041	1.03, (1.00, 1.06) | 0.036	1.03, (1.00, 1.06) | 0.020	1.03, (1.00, 1.06) | 0.018
Stoke/Davies Score**	1.15, (0.90, 1.48) | 0.258	1.17, (0.92, 1.49) | 0.194	1.18, (0.92, 1.50) | 0.178	1.16, (0.89, 1.46) | 0.248	1.20, (0.93, 1.52) | 0.142	1.18, (0.92, 1.51) | 0.189
Initial dialysis dose** (Incremental vs Full)	3.85, (1.89, 7.57) | <0.001	3.85, (1.82, 7.65) | <0.001	3.51, (1.71, 6.96) | 0.001	3.92, (1.92, 7.51) | 0.001	3.52, (1.75, 6.77) | 0.001	3.85, (1.90, 7.46) | <0.001
Creatinine (mg/dL)**	0.84, (0.74, 0.94) | 0.002	0.84, (0.74, 0.94) | 0.001	0.84, (0.75, 0.94) | 0.003	0.84, (0.74, 0.94) | 0.002	0.85, (0.75, 0.94) | 0.003	0.85, (0.75, 0.95) | 0.004
Urea (g/L)**	1.30, (1.09, 1.52) | 0.002	1.31, (1.11, 1.54) | 0.001	1.30, (1.10, 1.54) | 0.002	1.32, (1.11, 1.57) | <0.001	1.30, (1.10, 1.53) | 0.004	1.33, (1.12, 1.57) | <0.001
Cholesterol (mmol/L)**	1.30, (1.08, 1.54) | 0.007	1.29, (1.07, 1.54) | 0.007	1.29, (1.07, 1.53) | 0.007	1.28, (1.06, 1.52) | 0.013	1.29, (1.08, 1.51) | 0.005	1.28, (1.06, 1.51) | 0.011
Albumin (g/L)*****	1.28, (1.20, 1.37) | <0.001	1.27, (1.19, 1.36) | <0.001	1.26, (1.18, 1.34) | <0.001	1.28, (1.20, 1.38) | <0.001	1.22, (1.16, 1.29) | <0.001	1.24, (1.17, 1.32) | <0.001
Weibull shape	5.10, (3.86, 6.89) | <0.001	5.20, (3.90, 7.09) | <0.001	5.25, (3.94, 7.14) | <0.001	5.18, (3.88, 7.11) | <0.001	5.30, (4.02, 7.10) | <0.001	5.29, (3.97, 7.25) | <0.001

Table continued on next page

Table S8 (Continued). Posterior summaries of the joint model parameters fitted to the IDEAL PD training dataset accounting for competing risks.

Parameter	nor-nor-cr<br>Mean (95% CI) | P-value*	t-t-mod1-cr<br>Mean (95% CI) | P-value*	t-t_mod2-cr<br>Mean (95% CI) | P-value*	nor-t-mod2-cr<br>Mean (95% CI) | P-value*	t-t-mod3-cr<br>Mean (95% CI) | P-value*	nor-t-mod3-cr<br>Mean (95% CI) | P-value*
**Transfer to HD sub-model hazard ratios**						
Age**	0.97, (0.95, 1.00) | 0.031	0.97, (0.95, 1.00) | 0.034	0.97, (0.95, 1.00) | 0.034	0.97, (0.95, 1.00) | 0.032	0.97, (0.95, 1.00) | 0.032	0.97, (0.95, 1.00) | 0.029
BMI** (kg/m^2)	1.10, (1.05, 1.16) | <0.001	1.10, (1.05, 1.16) | <0.001	1.10, (1.05, 1.16) | <0.001	1.10, (1.05, 1.16) | <0.001	1.10, (1.05, 1.16) | <0.001	1.10, (1.05, 1.16) | <0.001
Smoking status** (Former vs Never)	2.11, (1.17, 3.93) | 0.011	2.09, (1.16, 3.98) | 0.013	2.10, (1.17, 3.94) | 0.013	2.11, (1.18, 4.07) | 0.010	2.13, (1.20, 3.96) | 0.010	2.11, (1.18, 3.97) | 0.011
Smoking status** (Current vs Never)	0.59, (0.14, 1.96) | 0.431	0.58, (0.15, 1.85) | 0.419	0.58, (0.14, 1.89) | 0.419	0.58, (0.14, 1.90) | 0.427	0.61, (0.15, 1.89) | 0.460	0.58, (0.14, 1.94) | 0.438
# of peritonitis episodes****	1.16, (1.00, 1.33) | 0.048	1.16, (1.00, 1.33) | 0.045	1.16, (1.00, 1.34) | 0.057	1.16, (1.00, 1.33) | 0.054	1.15, (0.99, 1.33) | 0.063	1.16, (1.00, 1.33) | 0.049
Albumin******	0.96, (0.91, 1.03) | 0.234	0.97, (0.91, 1.03) | 0.330	0.96, (0.91, 1.03) | 0.253	0.96, (0.90, 1.03) | 0.272	0.96, (0.91, 1.02) | 0.240	0.96, (0.91, 1.02) | 0.218
Weibull shape	3.52, (2.62, 4.83) | <0.001	3.53, (2.66, 4.81) | <0.001	3.53, (2.66, 4.83) | <0.001	3.55, (2.67, 4.82) | <0.001	3.58, (2.67, 4.82) | <0.001	3.53, (2.67, 4.79) | <0.001
**Transplantation sub-model hazard ratios**						
Age**	0.92, (0.89, 0.95) | <0.001	0.92, (0.89, 0.95) | <0.001	0.92, (0.89, 0.95) | <0.001	0.92, (0.89, 0.95) | <0.001	0.92, (0.89, 0.95) | <0.001	0.92, (0.89, 0.95) | <0.001
Ethnicity (Caucasian vs Non-Caucasian)	7.17, (2.75, 21.49) | <0.001	7.03, (2.59, 21.64) | <0.001	7.06, (2.74, 21.64) | <0.001	7.06, (2.77, 21.43) | <0.001	7.22, (2.83, 21.61) | <0.001	7.09, (2.66, 22.39) | <0.001
Stoke/Davies Score**	0.43, (0.20, 0.79) | 0.005	0.44, (0.21, 0.80) | 0.006	0.44, (0.20, 0.81) | 0.007	0.44, (0.21, 0.83) | 0.007	0.44, (0.22, 0.82) | 0.003	0.44, (0.21, 0.81) | 0.005
Smoking status** (Former vs Never)	0.70, (0.34, 1.43) | 0.312	0.70, (0.35, 1.44) | 0.330	0.71, (0.34, 1.44) | 0.350	0.70, (0.34, 1.45) | 0.341	0.71, (0.34, 1.44) | 0.371	0.71, (0.34, 1.48) | 0.335
Smoking status** (Current vs Never)	0.20, (0.06, 0.61) | 0.003	0.20, (0.06, 0.59) | 0.003	0.20, (0.05, 0.61) | 0.003	0.21, (0.06, 0.61) | 0.003	0.20, (0.05, 0.56) | 0.001	0.20, (0.06, 0.61) | 0.002
Albumin******	1.07, (0.97, 1.20) | 0.183	1.09, (0.98, 1.22) | 0.124	1.09, (0.99, 1.23) | 0.093	1.09, (0.98, 1.22) | 0.114	1.08, (0.98, 1.20) | 0.130	1.08, (0.98, 1.21) | 0.137
Weibull shape	4.79, (3.25, 7.41) | <0.001	4.77, (3.27, 7.30) | <0.001	4.77, (3.25, 7.34) | <0.001	4.77, (3.25, 7.34) | <0.001	4.81, (3.23, 7.29) | <0.001	4.72, (3.21, 7.17) | <0.001

BMI: Body mass index; CI: credible interval; HD: hemodialysis; PD: peritoneal dialysis.

* Posterior means are given along with 95% credible intervals. P-values were calculated based on the posterior distribution tail probabilities for containing the zero value.

** Values taken at baseline.

*** γ denotes the degrees of freedom parameter from *t*-distribution for both random and residual error applicable to '*t-t-mod1-cr*' only; ϕ and δ denote the degrees of freedom parameter for *t*-distributed random effects for random effects and residual error, respectively.

**** Number of peritonitis episodes is taken as the cumulative number of episodes at each follow-up and entered as a time-dependent variable in the model.

***** Hazard ratio per 1 g/L decrease in albumin is showed.

****** Hazard ratio per 1 g/L increase in albumin is showed.

Parameters from all JM applied to the IDEAL trial PD training dataset demonstrated excellent estimation properties, i.e. convergence as assessed by the R-hat metric below the recommended threshold of 1.1 for Bayesian estimation[8] as shown in Tables S8 and S9 below.

Table S9. Convergence of model parameters for JM without competing risks.

Parameter	nor-nor R-hat*	t-t-mod1 R-hat*	t-t-mod2 R-hat*	nor-t-mod2 R-hat*	t-t-mod3 R-hat*	nor-t-mod3 R-hat*
**Albumin sub-model**						
Intercept	1.001	1.003	1.001	1.000	1.006	1.002
Time on PD	1.001	1.002	1.001	1.001	1.005	1.004
Gender (Male)	1.001	1.002	1.000	1.000	1.001	1.001
BMI (kg/m^2)	1.003	1.000	1.001	1.001	1.007	1.000
Stoke/Davies Score	1.001	1.001	1.001	1.001	1.005	1.000
Calcium (mmol/L)	1.001	1.002	1.000	1.000	1.008	1.001
Creatinine (mmol/L)	1.000	1.006	1.000	1.002	1.004	1.001
Haemoglobin (g/L)	1.000	1.000	1.000	1.000	1.004	1.001
$\Sigma(1,1)$	1.000	1.001	1.003	1.000	1.006	1.000
$\Sigma(1,2)$	1.000	1.005	1.006	1.001	1.006	1.001
$\Sigma(2,2)$	1.000	1.001	1.001	1.001	1.000	1.005
γ**	—	1.000	—	—	—	—
ϕ	—	—	1.012	—	1.005	—
σ	1.000	1.000	1.000	1.001	1.008	1.001
δ	—	—	1.000	1.001	1.004	1.003
**Survival sub-model**						
Gender (Male)	1.000	1.000	1.000	1.000	1.000	1.000
Age	1.000	1.000	1.000	1.000	1.000	1.000
Stoke/Davies Score	1.000	1.000	1.000	1.000	1.001	1.000
Initial dialysis dose (Incremental vs Full)	1.000	1.000	1.000	1.000	1.001	1.000
Creatinine	1.001	1.000	1.000	1.000	1.002	1.000
Urea	1.000	1.000	1.000	1.000	1.002	1.000
Cholesterol	1.000	1.000	1.000	1.000	1.003	1.000
Albumin	1.001	1.000	1.000	1.000	1.004	1.000
Weibull shape	1.000	1.001	1.000	1.001	1.001	1.000

BMI: Body mass index; PD: peritoneal dialysis.

* R-hat is reported directly from Stan software based on the posterior chains and calculated as the maximum of rank normalized split-R-hat and rank normalized folded-split-R-hat.

** Parameter applicable to '*t-t-mod1*' only.

Table S10. Convergence of model parameters for JM with competing risks.

Parameter	nor-nor-cr	t-t-mod1-cr	t-t_mod2-cr	nor-t-mod2-cr	t-t-mod3-cr	nor-t-mod3-cr
	R-hat*	R-hat*	R-hat*	R-hat*	R-hat*	R-hat*
**Albumin sub-model**						
Intercept	1.001	1.002	1.001	1.002	1.023	1.000
Time on PD	1.004	1.002	1.001	1.000	1.002	1.000
Gender (Male)	1.001	1.002	1.000	1.000	1.008	1.000
BMI (kg/m^2)	1.000	1.002	1.001	1.001	1.003	1.001
Stoke/Davies Score	1.002	1.001	1.001	1.000	1.001	1.001
Calcium (mmol/L)	1.001	1.001	1.001	1.004	1.008	1.000
Creatinine (mmol/L)	1.002	1.001	1.000	1.002	1.005	1.001
Haemoglobin (g/L)	1.000	1.000	1.002	1.003	1.011	1.001
$\Sigma(1,1)$	1.001	1.000	1.002	1.003	1.008	1.000
$\Sigma(1,2)$	1.001	1.001	1.001	1.002	1.000	1.001
$\Sigma(2,2)$	1.001	1.001	1.000	1.001	1.003	1.000
γ**	—	1.000	—	—	—	—
ϕ	—	—	1.003	—	1.003	—
σ	1.000	1.000	1.000	1.000	1.009	1.004
δ	—	—	1.001	1.000	1.005	1.005
**Survival sub-model**						
Gender (Male)	1.000	1.000	1.000	1.000	1.000	1.000
Age	1.000	1.000	1.000	1.000	1.002	1.000
Stoke/Davies Score	1.000	1.000	1.000	1.000	1.005	1.000
Initial dialysis dose (Incremental vs Full)	1.000	1.000	1.000	1.000	1.001	1.000
Creatinine	1.000	1.000	1.000	1.000	1.006	1.000
Urea	1.000	1.000	1.000	1.001	1.006	1.000
Cholesterol	1.000	1.000	1.000	1.000	1.000	1.000
Albumin	1.001	1.000	1.000	1.001	1.002	1.000
Weibull shape	1.000	1.000	1.000	1.001	1.003	1.000

Table continued on next page

Table S10 (Continued). Convergence of model parameters for JM with competing risks.

Parameter	nor-nor-cr	t-t-mod1-cr	t-t_mod2-cr	nor-t-mod2-cr	t-t-mod3-cr	nor-t-mod3-cr
	R-hat*	R-hat*	R-hat*	R-hat*	R-hat*	R-hat*
**Transfer to HD sub-model**						
Age	1.000	1.000	1.000	1.000	1.000	1.000
BMI (kg/m^2)	1.000	1.000	1.000	1.000	1.001	1.000
Smoking status (Former vs Never)	1.000	1.000	1.000	1.000	1.001	1.000
Smoking status (Current vs Never)	1.000	1.000	1.000	1.000	1.003	1.000
# of peritonitis episodes***	1.000	1.000	1.000	1.000	1.011	1.000
Albumin	1.000	1.000	1.000	1.000	1.005	1.000
Weibull shape	1.000	1.000	1.000	1.000	1.008	1.000
**Transplantation sub-model**						
Age	1.000	1.000	1.000	1.000	1.012	1.000
Ethnicity (Caucasian vs Non-Caucasian)	1.000	1.000	1.000	1.000	1.002	1.000
Stoke/Davies Score	1.000	1.000	1.000	1.000	1.001	1.000
Smoking status (Former vs Never)	1.000	1.000	1.000	1.000	1.004	1.000
Smoking status (Current vs Never)	1.000	1.000	1.000	1.000	1.001	1.000
Albumin	1.000	1.000	1.000	1.000	1.001	1.000
Weibull shape	1.000	1.000	1.000	1.000	1.006	1.000

BMI: Body mass index; HD: hemodialysis; PD: peritoneal dialysis.

* R-hat is reported directly from Stan software based on the posterior chains and calculated as the maximum of rank normalized split-R-hat and rank normalized folded-split-R-hat.

** Parameter applicable to '*t-t-mod1*' only.
*** Number of peritonitis episodes is taken as the cumulative number

3.5. Outlier Detection and Identification with Robust Joint Models

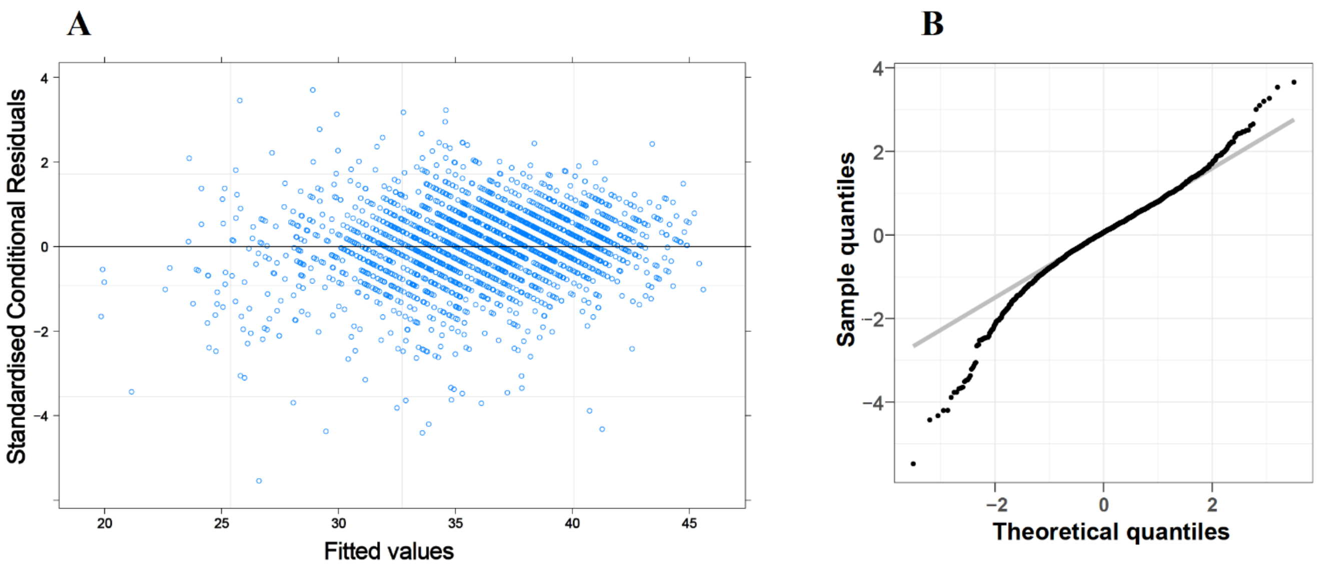

Figure S6. Visual inspection of the standardised conditional residuals obtained from the standard joint model with Gaussian assumptions (‘*nor-nor*’) fitted to the IDEAL PD training dataset. A: standardised conditional residuals vs fitted values; B: quantile–quantile (Q-Q) plot of standardised conditional residuals against standard Normal distribution.

As mentioned in the main text, the flexible specification of the ‘*t-t-mod3*’ could help quantify and detect both *b*- and *e*-outliers. With respect to outlying patients, concentration of the posterior distribution of V_i on values greater than 1 indicates that an individual's behaviour deviates significantly from the general population, so that the patient i can be considered a *b*-outlier as the random intercept and/or random slope fail to align with the trends observed in the population. Figure S7 below shows the individual model fit from ‘*nor-nor’* and ‘*t-t-mod3*’ models for patient 60002 from the IDEAL PD training dataset along with a summary for the posterior distribution for V_i for that patient. The 95% credible interval for V_i suggests that patient is a *b*-outlier and in fact, the patient has the lowest baseline value of albumin of the entire dataset (17 g/L compared with a mean baseline value of 37.23 g/L for the dataset). Concerning the baseline risk factors in the survival sub-model, the patient was female, with a Stoke/Davies score of 1, treated with incremental dialysis, at an older age than the average (62.5 years vs 60.6 for the training dataset) and lower values of serum creatine (5.94 mmol/L vs mean of 10.3 mmol/L for the sample), serum urea (4 mmol/L vs mean of 5.4 mmol/L for the sample) and cholesterol (2.78 mmol/L vs mean of 4.7 mmol/L for the sample). Visually, the plot in Figure S7 shows that the ‘*t-t-mod3*’ model (in blue) presents a better fit for this outlying patient than the standard ‘*nor-nor*’ (in red), whereby the population average trajectory estimated from ‘*t-t-mod3*’ (in green) is far above this individual as expected since the patient has the lowest baseline albumin and provides only 2 post-baseline records before death at 0.47 years following start of peritoneal dialysis.

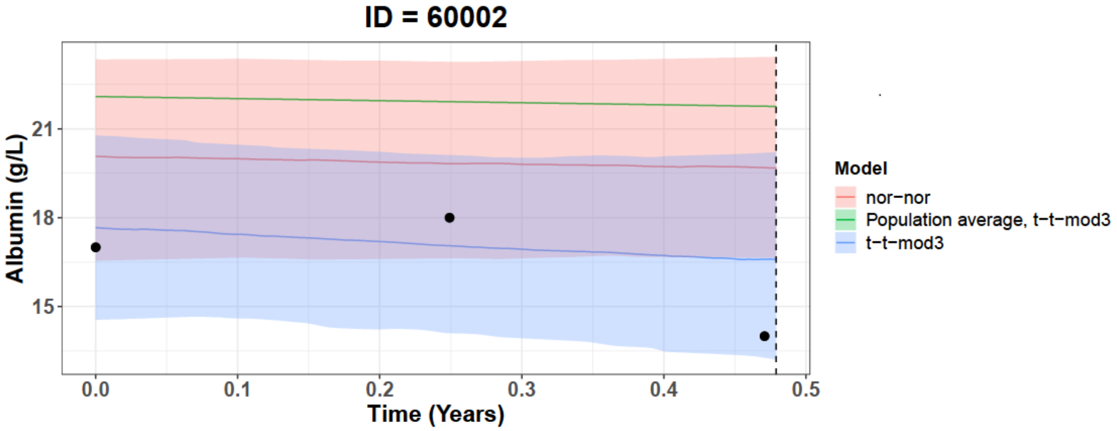

Posterior summary of V_i

Model	Quantile	V
t_t_mod3	2.5%	1.72
t_t_mod3	97.5%	50.15

Figure S7. Example of a *b*-outlier with robust joint models. Individual model fits from models ‘*nor-nor*’ and ‘*t-t-mod3*’ are shown along with posterior summaries of $\boldsymbol{V_i}$ from model ‘*t-t-mod3*’ for patient 6002 from IDEAL trial PD training dataset. Black dots indicate individual albumin values collected from the patient. Shaded areas illustrate 95% credible intervals for posterior predictive albumin values for the patient, population average trajectory estimated from model ‘*t-t-mod3*’ is displayed in green. $\boldsymbol{V_i}$ denotes the individual mixing variate for the distribution of random subject effects (Section 1.3.2).

Considering outlying observations within an individual’s trajectory, the posterior distributions of the W_{ij} from the ‘*t-t-mod3*’ fitted to the data could help indicate such *e*-outliers. Concentration of the posterior distribution of W_{ij} on values greater than 1 would suggest that biomarker observation j from patient i deviate sharply from the trend of the individual, hence it could be marked as an *e*-outlier. Figure S8 below demonstrates individual model fits for three patients from the IDEAL trial PD training dataset with both standard ‘*nor-nor*’ model as well as the best fitting robust ‘*t-t-mod3’* model, with summaries of the posterior

distribution for W_{ij} in tabular form for each patient. The individual albumin trajectory plot for patient 11064 (top left panel) suggests the presence of two outlying negative observations at times 2.2 and 2.4 years after start of peritoneal dialysis, which were confirmed as such from the '*t-t-mod3'* model with 95% CI for W_{ij} of the two points as (4.31, 73.08) and (2.06, 40.73), which do not include 1. Similarly, for patient 107002 (top right panel) the second last observation at time 3 years after PD start is detected as an *e*-outlier with the 95% CI for W_{ij} at (3.58, 77.07). Finally, for patient 8002 no points stand out within the longitudinal trajectory of the patient and the posterior summary for W_{ij} across all records indicate that 1 is within the credibility intervals, confirming the visual impression that no *e*-outliers occur for this patient. Figure S8 also provides a visual illustration of the utility of robust joint models to downweigh outlying observations. Namely, the discrepancy between individual fitted trajectories from '*nor-nor*' model (in red) and '*t-t-mod3*' model (in blue) for the first two patients with detected outlying records, suggest that normal distribution assumptions result into a far steeper trajectory of decline for patients where outlying low albumin values are present in contrast with a flatter and less susceptible to extreme observations trajectory estimated with the robust '*t-t-mod3*' model. Interestingly, for the third patient 8002 (lower left panel), for which no *e*-outliers were detected, the fitted trajectories from these two models, albeit very different in structural assumptions, almost completely coincide. This is well in line with literature on robust mixed models pointing out the flexibility of the *t*-distributional assumption to closely approximate results from normal distribution in the absence of outliers.

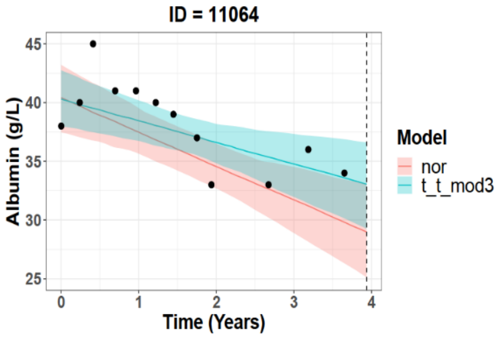

Posterior summary of Wij

Model	Quantile	t=0	t=0.2	t=0.4	t=0.7	t=1	t=1.2	t=1.4	t=1.7	t=1.9	t=2.2	t=2.4	t=2.7	t=3.2	t=3.6
t_t_mod3	2.5%	0.37	0.32	0.64	0.36	0.37	0.34	0.32	0.31	0.49	4.31	2.06	0.35	0.34	0.31
t_t_mod3	97.5%	6.73	5.52	12.07	5.99	6.95	5.86	6.19	5.14	8.45	73.08	40.73	6.80	5.74	5.49

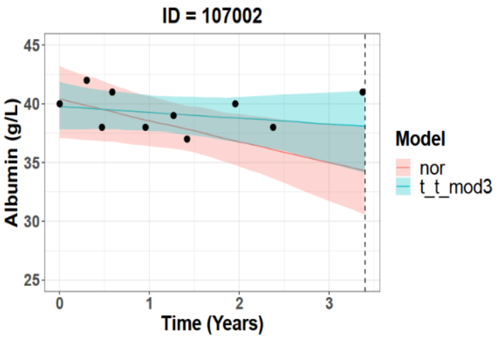

Posterior summary of Wij

Model	Quantile	t=0	t=0.3	t=0.5	t=0.6	t=1	t=1.3	t=1.4	t=2	t=2.4	t=3	t=3.4
t_t_mod3	2.5%	0.32	0.33	0.32	0.34	0.30	0.30	0.34	0.33	0.32	3.58	0.39
t_t_mod3	97.5%	5.25	6.48	5.83	5.84	5.46	26.57	6.01	5.48	5.43	77.07	7.82

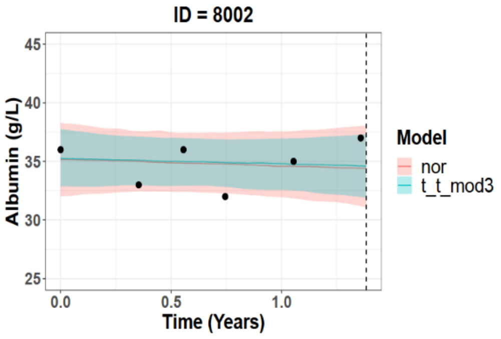

Posterior summary of Wij

Model	Quantile	t=0	t=0.4	t=0.6	t=0.7	t=1.1	t=1.4
t_t_mod3	2.5%	0.32	0.35	0.33	0.41	0.32	0.36
t_t_mod3	97.5%	5.78	6.63	5.97	7.55	5.58	6.87

Figure S8. Detection of *e*-outliers with robust JM results from individual patients. Individual model fits from models '*nor-nor*' and '*t-t-mod3*' are shown along with posterior summaries of $\boldsymbol{W_{ij}}$ from model '*t-t-mod3*' for three patients from IDEAL trial PD testing dataset. Shaded areas illustrate 95% credible intervals for posterior predictive albumin values for the patients. $\boldsymbol{W_{ij}}$ denotes the individual mixing variate for the distribution of residual error (Section 1.3.2).

In light of the above results, Mahalanobis distance (δ^2), as introduced by Pinheiro et al. (2001)[36], being a general metric for the detection and identification of outliers independent of the JM structure, is calculated for models '*nor-nor*' and '*t-t-mod3*'. It is plotted in Figures S9 and S10 below, where each dot represents a value of δ_b^2, δ_e^2 and δ^2 corresponding to a specific patient from the IDEAL trial PD training dataset.

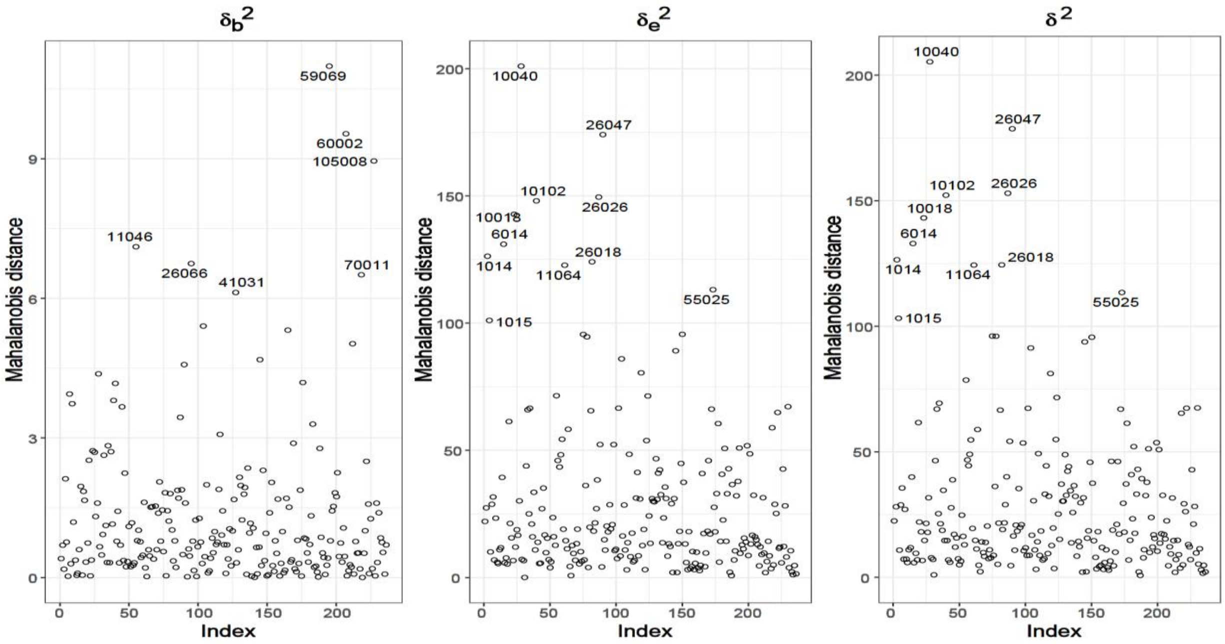

Figure S9. Mahalanobis distance decomposition plot estimated from the standard JM '*nor-nor'*. Mahalanobis distance ($\boldsymbol{\delta^2}$) in right panel is decomposed into the sum of $\boldsymbol{\delta_b^2}$ (for detection of *b*-outliers, left panel) and $\boldsymbol{\delta_e^2}$ (for detection of *e*-outliers, middle panel).

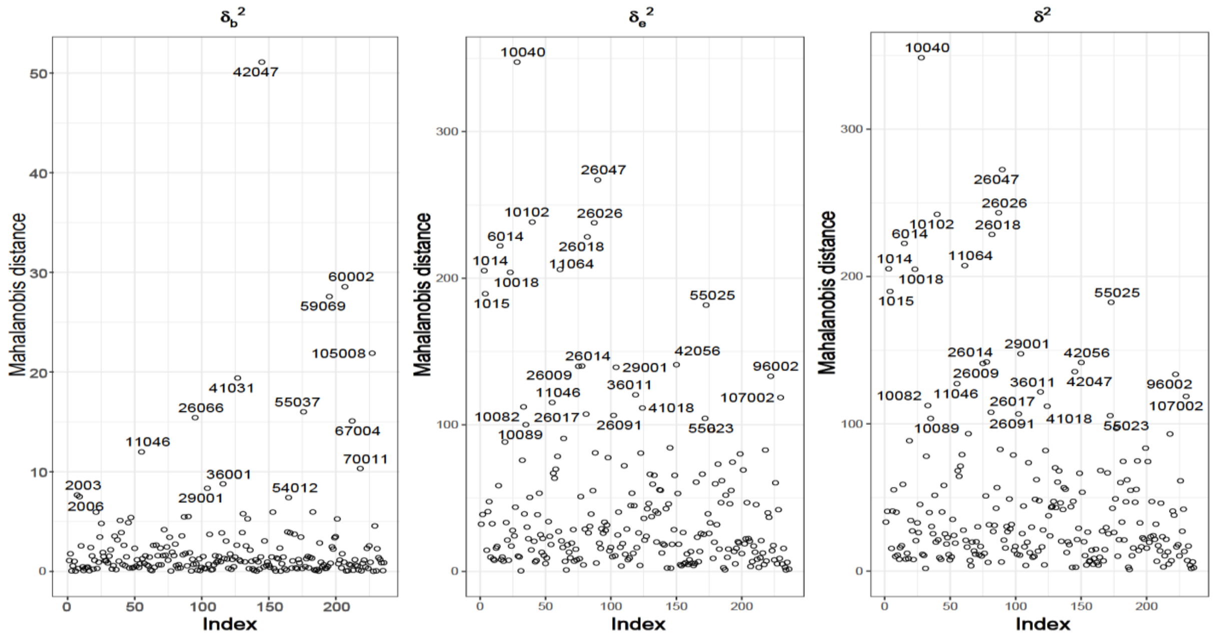

Figure S10. Mahalanobis distance decomposition plot estimated from the robust JM '*t-t-mod3'*. Mahalanobis distance ($\boldsymbol{\delta^2}$) in right panel is decomposed into the sum of $\boldsymbol{\delta_b^2}$ (for detection of *b*-outliers, left panel) and $\boldsymbol{\delta_e^2}$ (for detection of *e*-outliers, middle panel).

As can be observed from the figures, the application of the robust JM '*t-t-mod3*' leads to the identification of 15 patients as *b*-outliers in terms of their albumin trajectory (whose value for δ_b^2 exceeded the arbitrary threshold of 6) as opposed to just 7 patients from the '*nor-nor*' model. Similarly, 25 patients were identified as *e*-outliers (whose value for δ_e^2 exceeded the arbitrary threshold of 100) with the help of '*t-t-mod3*' model versus 11 from the '*nor-nor*' model. Of note, no patients were identified as both *b*- and *e*-outliers from '*nor-nor*' model as judged by the total Mahalanobis distance, whereas model '*t-t-mod3*' identified two patients as both a *b*- and an *e*-outlier (values of δ^2 exceeding the arbitrary threshold of 100). That suggests that for the IDEAL clinical trial dataset the sets of patients considered as outlying from the population and the patients with outlying albumin values within their individual trajectories tend to be disjoint. Mahalanobis distance can be used to check agreement with the examples for *b*- and *e*-outliers from Figures S7 and S8. Mahalanobis metric from both '*nor-nor*' and '*t-t-mod3*' models identified patient 60002 from Figure S7 as a serious outlier within the population of patients as demonstrated by the high values of estimated δ_b^2 in the left panels of Figures S9 and S10. Furthermore, patient 11064 from Figure S8 was detected as an *e*-outlier using Mahalanobis distance criterion from both models, whereas patient 107002 was detected an *e*-outlier by model '*t-t-mod3*' only. As patient 11064 had two outlying observations and patient 107002 had only one, the results from Mahalanobis distance suggest the greater sensitivity of model '*t-t-mod3*' for the detection of outliers.

3.6. Results from Cox PH Models for All-cause Mortality

As follows from the main text, two Cox models were fitted to the IDEAL PD training dataset for comparison with the survival sub-model results from the various joint models. The first one has baseline albumin (‘*Cox-1*’) and the second is an extended Cox model with albumin as a time-varying covariate (‘*Cox-2*’), which both share the same baseline risk factors as the survival sub-model. The two Cox models for survival were fitted with the *coxph*() function in **R** under frequentist estimation. Results are shown in Table S11 below.

Table S11. Survival analysis with Cox proportional hazards models ignoring competing risks.

Hazard Ratio	Cox-1<br>Mean (95% CI) | P-value	Cox-2<br>Mean (95% CI) | P-value
Gender (Male)	0.88, (0.54, 1.46) | 0.617	1.73, (1.01, 2.98) | 0.042
Age	1.03, (1.00, 1.06) | 0.047	1.03, (1.00, 1.05) | 0.034
Stoke/Davies Score	1.31, (1.02, 1.67) | 0.028	1.32, (1.04, 1.66) | 0.018
Initial dialysis dose (Incremental vs Full)	3.43, (1.77, 6.62) | <0.001	3.46, (1.81, 6.64) | <0.001
Creatinine	0.86, (0.77, 0.97) | 0.012	0.86, (0.76, 0.96) | 0.008
Urea	1.38, (1.18, 1.63) | <0.001	1.26, (1.07, 1.48) | 0.004
Cholesterol	1.18, (1.00, 1.40) | 0.049	1.34, (1.13, 1.58) | <0.001
Albumin*	1.17, (1.11, 1.22) | <0.00	1.19, (1.15, 1.23) | <0.001

CI: confidence interval

* Baseline value was used for '*Cox-1'*. whereas extended Cox model *'Cox-2*' includes albumin as a time-varying covariate.

3.7. Additional Results from Dynamic Predictions for Various Prediction Horizons With IDEAL PD Testing Dataset.

Table S12. Patients at risk at selected landmark times and forecast horizons in IDEAL trial PD testing dataset (N=78). The table shows number of patients (and percentages) who were at risk, died, were transferred to HD (TrHD), had kidney transplantation (KTx) or were censored at the respective prediction horizons and landmark times (LMT).

	**At risk**	**Death**	**TrHD**	**KTx**	**Censored**
**Forecast horizon 6 months**					
LMT 1 year	61	4 (6.6%)	2 (3.3%)	1 (1.6%)	1 (1.6%)
LMT 1.5 years	53	4 (7.5%)	4 (7.5%)	4 (7.5%)	0 (0%)
LMT 2 years	41	3 (7.3%)	0 (0%)	0 (0%)	2 (4.9%)
**Forecast horizon 1 year**					
LMT 1 year	61	8 (13.1%)	6 (9.8%)	5 (8.2%)	1 (1.6%)
LMT 1.5 years	53	7 (13.2%)	4 (7.5%)	4 (7.5%)	2 (3.8%)
LMT 2 years	41	5 (12.2%)	1 (2.4%)	0 (0%)	4 (9.8%)

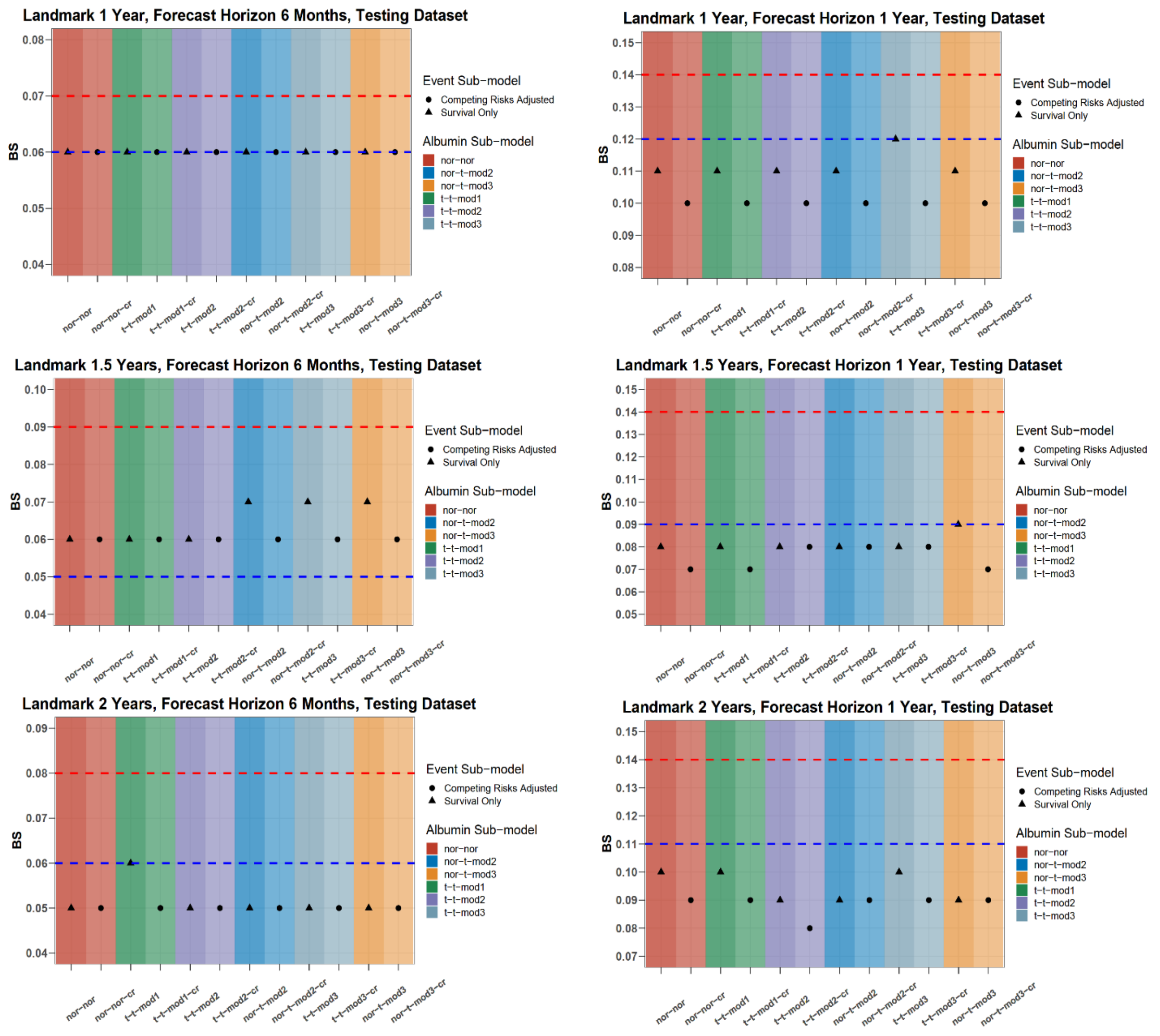

Figure S11. Brier score (BS) of dynamic predictions for all landmark times and two prediction horizons with IDEAL trial PD testing dataset. Lower BS values indicate better accuracy in terms of better calibration of the survival prognosis. Left column displays results for forecast horizons of 6 months, right column displays results for forecast horizons of 1 year. JM which ignore competing risks (black triangles) are placed right next to the JM extension which accounts for competing risks (black circles) grouped according to the longitudinal sub-model type (e.g., '*nor-nor-cr*' follows '*nor-nor*'). Red dashed horizontal line indicates result from Cox PH model with baseline albumin value ('Cox-1' in text) and blue dashed line indicates result from extended Cox PH model with albumin as a time-varying covariate ('Cox-2' in text).

Table S13. Brier score (BS) of dynamic predictions for three landmark times and various prediction horizons with IDEAL PD testing dataset.

Landmark time (Year)	Forecast Time (Year)	Cox-1	Cox-2	nor	nor-cr	t-t-mod1	t-t-mod1-cr	t-t-mod2	t-t-mod2-cr	nor-t-mod2	nor-t-mod2-cr	t-t-mod3	t-t-mod3-cr	nor-t-mod3	nor-t-mod3-cr
1	1.5	0.07	0.06	0.06	0.06	0.06	0.06	0.06	0.06	0.06	0.06	0.06	0.06	0.06	0.06
	2	0.14	0.12	0.11	0.1	0.11	0.1	0.11	0.1	0.11	0.1	0.12	0.1	0.11	0.1
	2.5	0.17	0.13	0.12	0.11	0.12	0.11	0.12	0.11	0.12	0.11	0.12	0.11	0.12	0.11
	3	0.21	0.17	0.14	0.12	0.14	0.13	0.14	0.13	0.14	0.13	0.14	0.13	0.14	0.13
	3.5	0.22	0.16	0.13	0.12	0.12	0.12	0.13	0.12	0.12	0.13	0.12	0.13	0.12	0.13
	4	0.29	0.25	0.19	0.17	0.19	0.17	0.19	0.17	0.19	0.18	0.19	0.18	0.19	0.18
	4.5	0.29	0.27	0.19	0.18	0.19	0.18	0.19	0.18	0.19	0.18	0.19	0.18	0.19	0.18
	5	0.33	0.26	0.16	0.19	0.16	0.19	0.17	0.19	0.16	0.19	0.17	0.19	0.16	0.19
1.5	2	0.09	0.05	0.06	0.06	0.06	0.06	0.06	0.06	0.07	0.06	0.07	0.06	0.07	0.06
	2.5	0.14	0.09	0.08	0.07	0.08	0.07	0.08	0.08	0.08	0.08	0.08	0.08	0.09	0.07
	3	0.18	0.13	0.11	0.1	0.11	0.1	0.1	0.1	0.11	0.1	0.11	0.11	0.12	0.1
	3.5	0.19	0.14	0.1	0.1	0.1	0.1	0.1	0.1	0.11	0.11	0.11	0.11	0.12	0.11
	4	0.28	0.23	0.18	0.16	0.18	0.17	0.18	0.17	0.19	0.17	0.19	0.17	0.2	0.17
	4.5	0.29	0.25	0.2	0.18	0.2	0.19	0.2	0.19	0.2	0.19	0.21	0.19	0.21	0.19
	5	0.34	0.27	0.18	0.2	0.19	0.21	0.19	0.21	0.2	0.21	0.2	0.21	0.2	0.21
2	2.5	0.08	0.06	0.05	0.05	0.06	0.05	0.05	0.05	0.05	0.05	0.05	0.05	0.05	0.05
	3	0.14	0.11	0.1	0.09	0.1	0.09	0.09	0.08	0.09	0.09	0.1	0.09	0.09	0.09
	3.5	0.15	0.11	0.1	0.1	0.1	0.09	0.09	0.1	0.09	0.1	0.1	0.1	0.1	0.1
	4	0.26	0.22	0.17	0.17	0.18	0.17	0.18	0.17	0.18	0.17	0.19	0.18	0.19	0.18
	4.5	0.29	0.23	0.19	0.19	0.21	0.19	0.21	0.2	0.2	0.19	0.22	0.2	0.21	0.2
	5	0.35	0.24	0.19	0.22	0.21	0.22	0.21	0.23	0.2	0.22	0.23	0.23	0.21	0.23

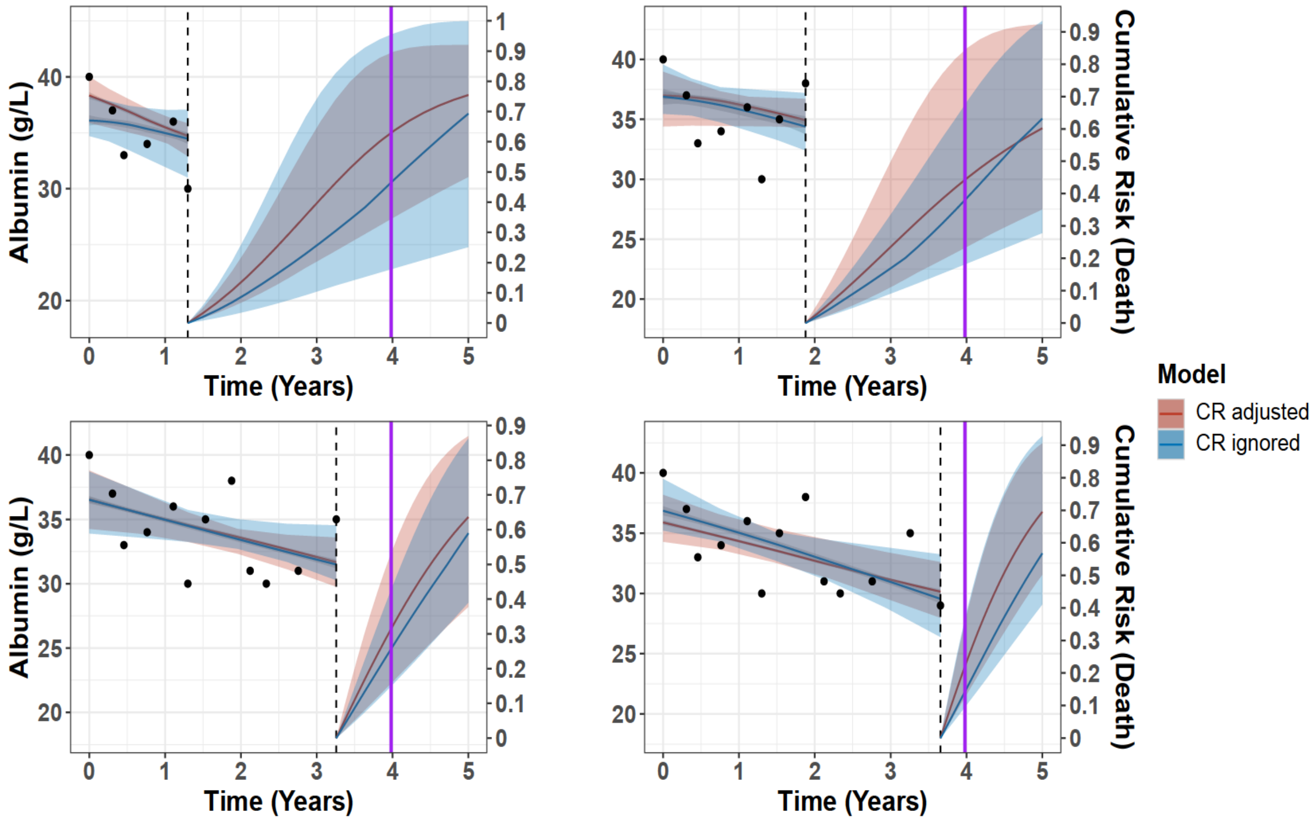

Figure S12. Dynamic predictions for a patient selected at random from the IDEAL trial PD testing data set calculated with the two models with best fit. Results from best-fitting model without competing risks '*t-t-mod3*' are in blue and results from the best-fitting model with competing risks, '*t-t-mod3-cr*', are in red. The x-axis represents years after PD start, and the vertical dotted line indicates the time point of the latest measurement. The y-axis of the left side represents the albumin measurements that are available up to the latest visit. In particular, the black circles represent the observed values and the solid line the fitted longitudinal trajectory. The y-axis on the right side represents the mean estimator of the predicted cumulative risk of death and the shaded area illustrates the corresponding 95% pointwise Bayesian intervals. The purple line indicates the occurrence of event to the patient, who died at time = 3.98 years after start of PD.

3.8. Calibration Plots for Dynamic Predictions in IDEAL PD Testing Dataset

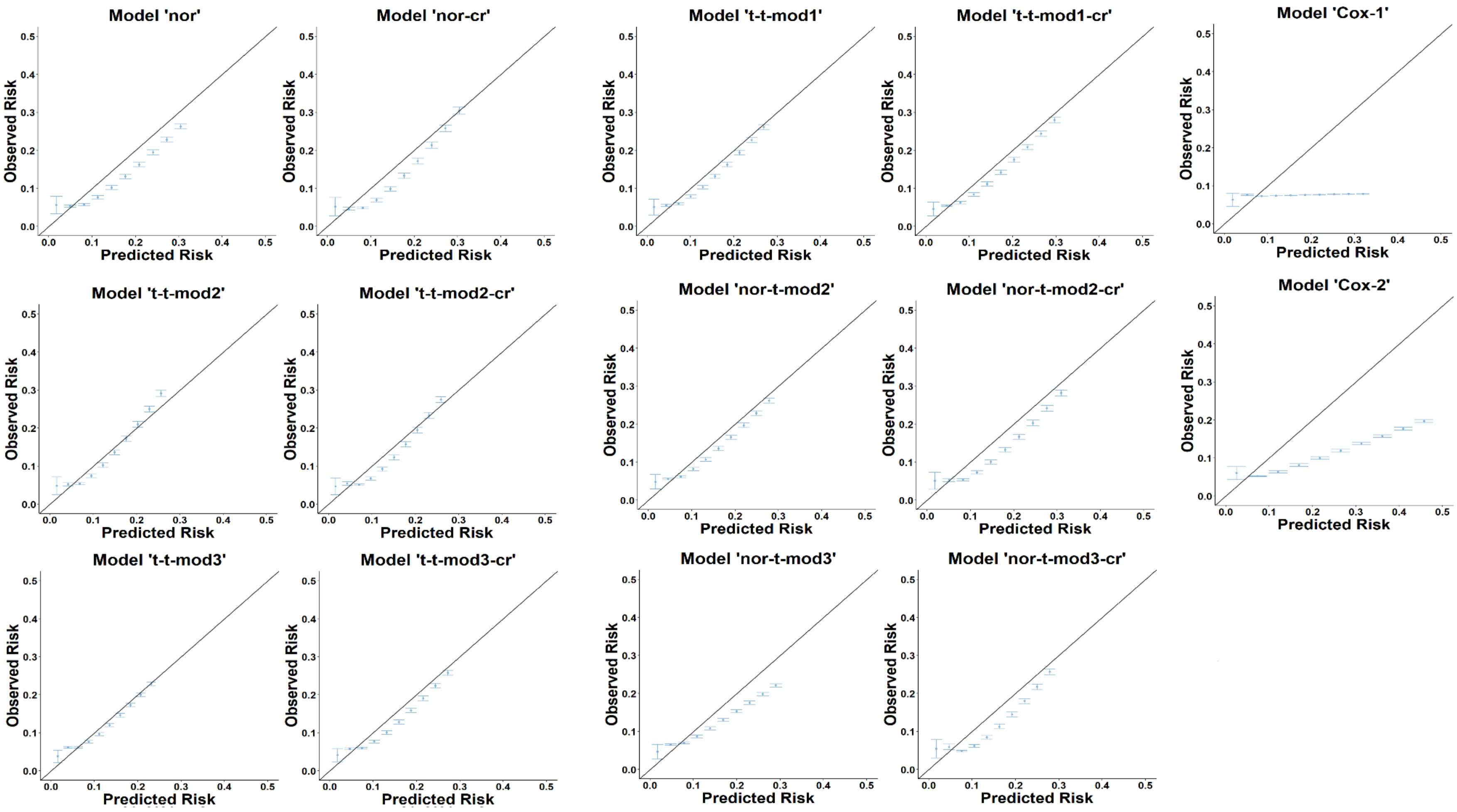

Figure S13. Calibration plots of all 12 joint models and the 2 Cox models in IDEAL trial PD testing dataset for landmark time 1 year, forecast horizon 6 months. Calibration plots compare predicted and observed mortality rates within deciles of risk score along with 95% confidence intervals. The number of patients at risk at selected landmark times and forecast horizons in IDEAL trial PD testing dataset, consisting of a total of 78 patients, are provided in main text.

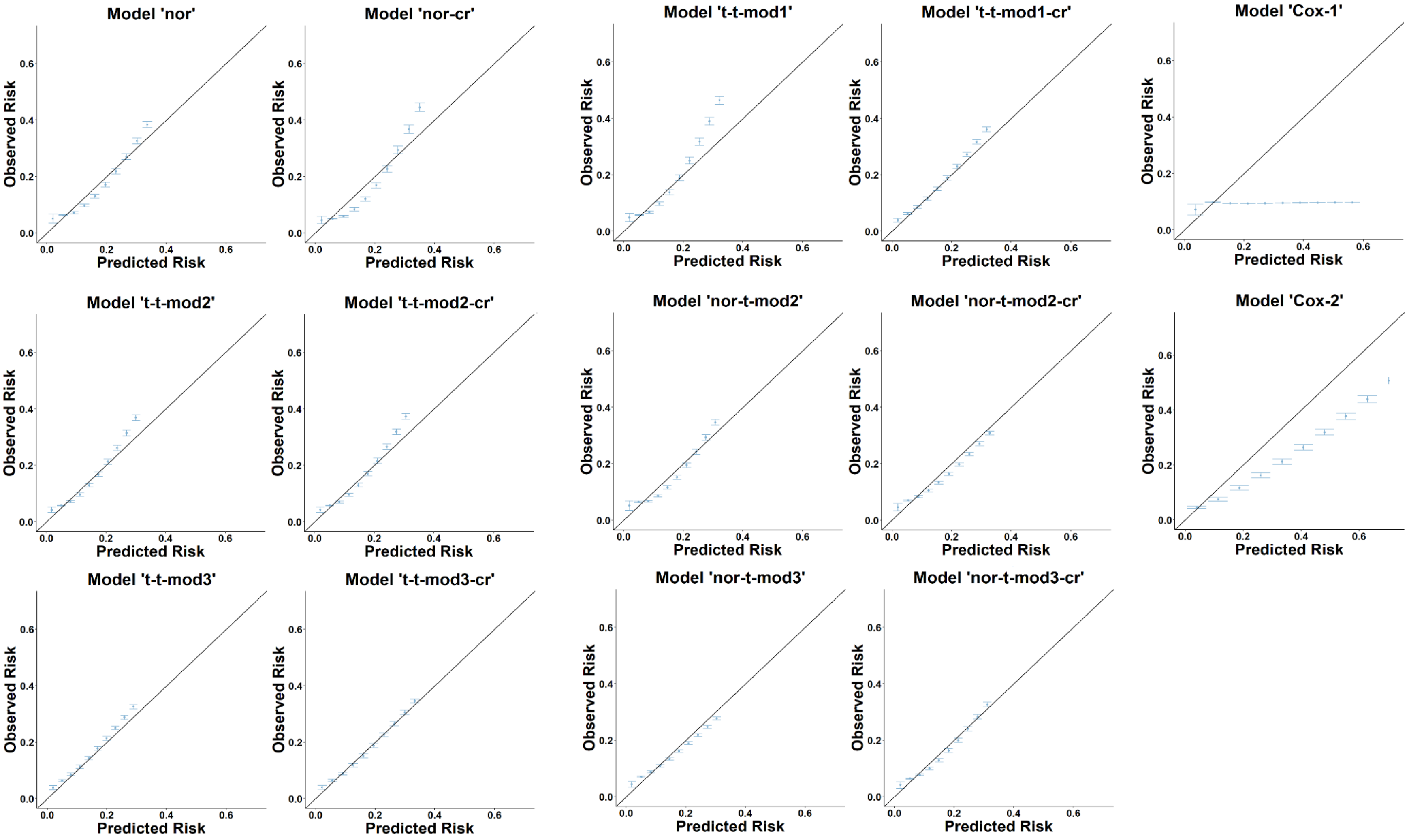

Figure S14. Calibration plots of all 12 joint models and the 2 Cox models in IDEAL trial PD testing dataset for landmark time 1.5 years, forecast horizon 6 months. Calibration plots compare predicted and observed mortality rates within deciles of risk score along with 95% confidence intervals. The number of patients at risk at selected landmark times and forecast horizons in IDEAL trial PD testing dataset, consisting of a total of 78 patients, are provided in main text.

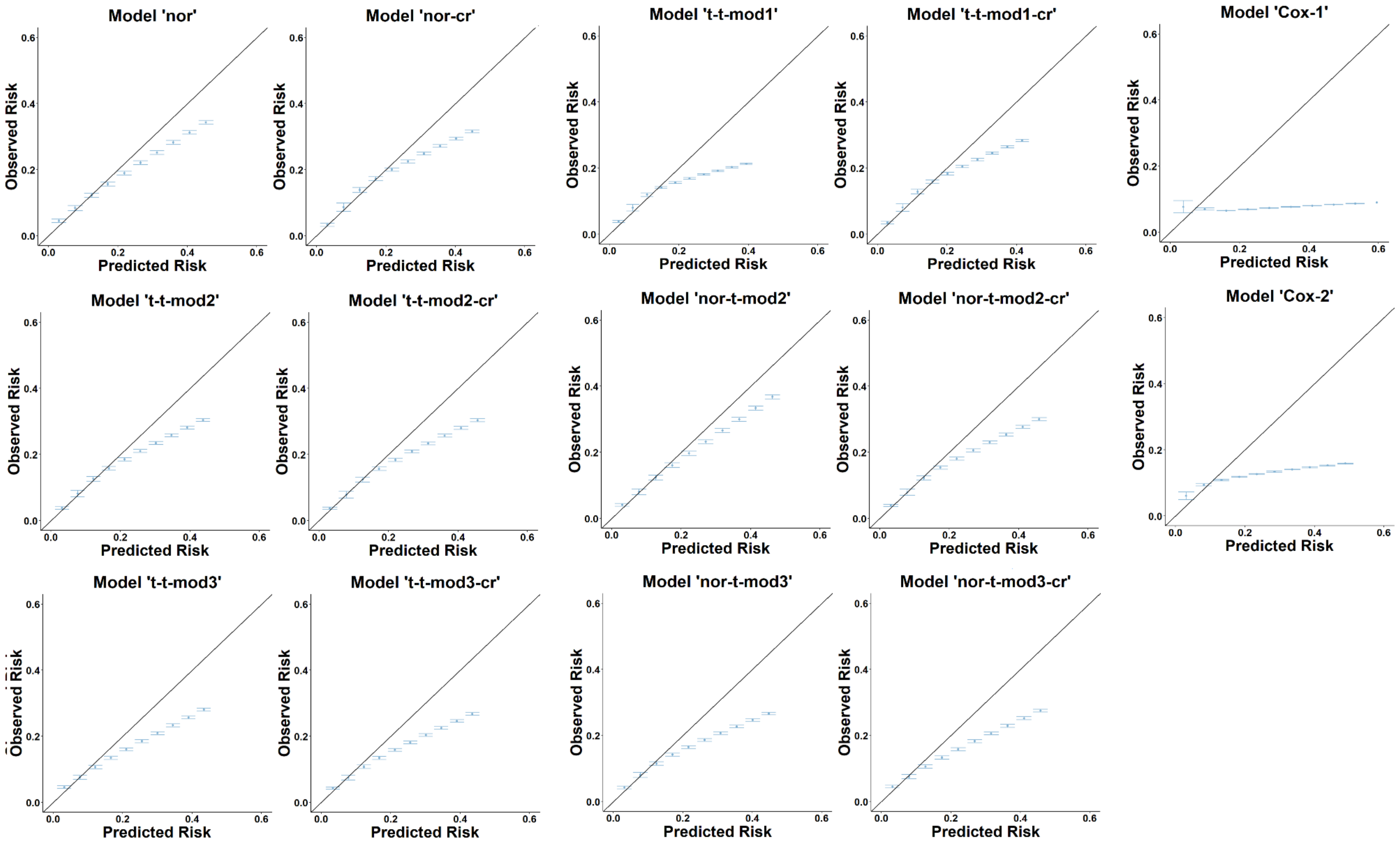

Figure S15. Calibration plots of all 12 joint models and the 2 Cox models in IDEAL trial PD testing dataset for landmark time 2 years, forecast horizon 6 months. Calibration plots compare predicted and observed mortality rates within deciles of risk score along with 95% confidence intervals. The number of patients at risk at selected landmark times and forecast horizons in IDEAL trial PD testing dataset, consisting of a total of 78 patients, are provided in main text.

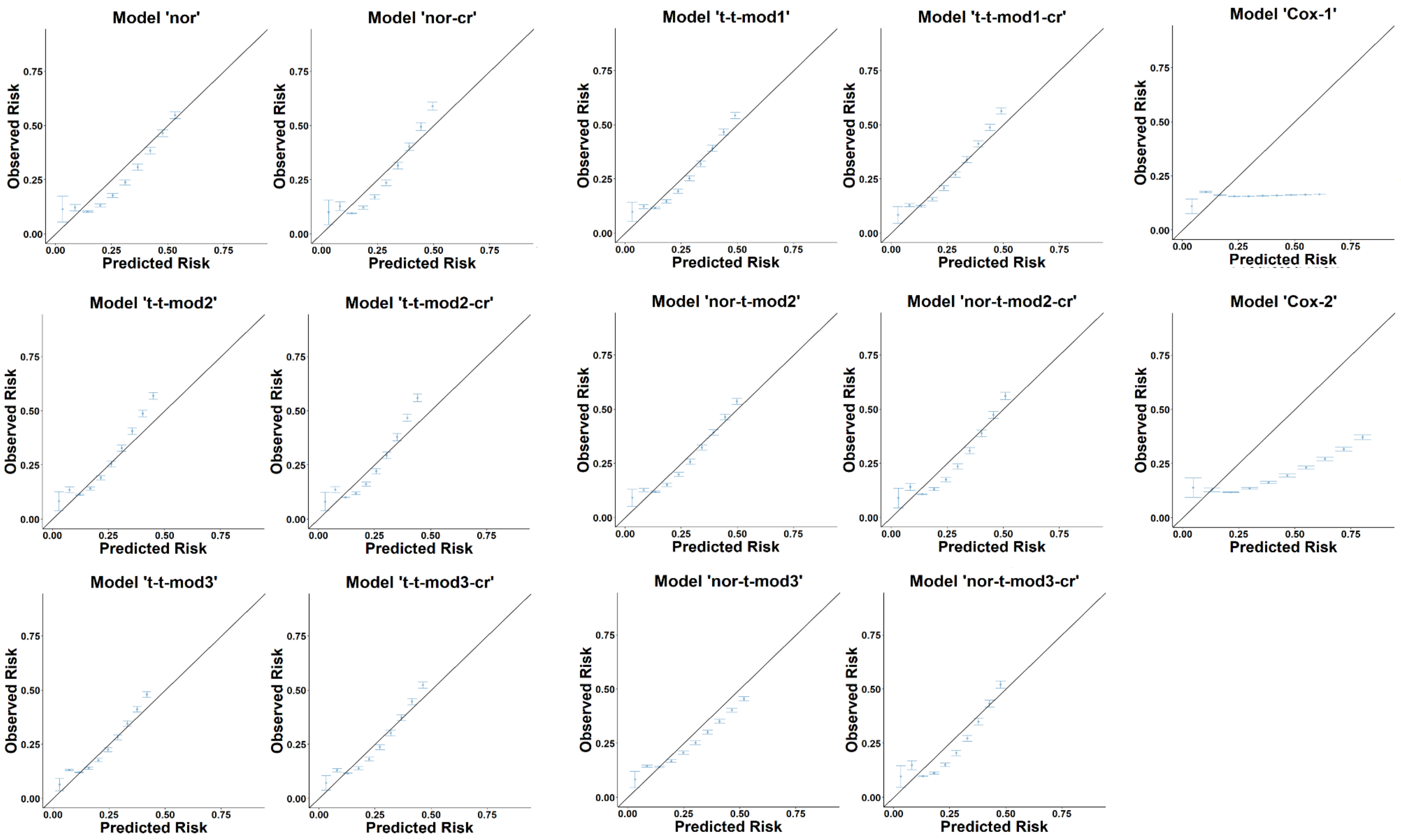

Figure S16. Calibration plots of all 12 joint models and the 2 Cox models in IDEAL trial PD testing dataset for landmark time 1 year, forecast horizon 1 year. Calibration plots compare predicted and observed mortality rates within deciles of risk score along with 95% confidence intervals. The number of patients at risk at selected landmark times and forecast horizons in IDEAL trial PD testing dataset, consisting of a total of 78 patients, are provided in main text.

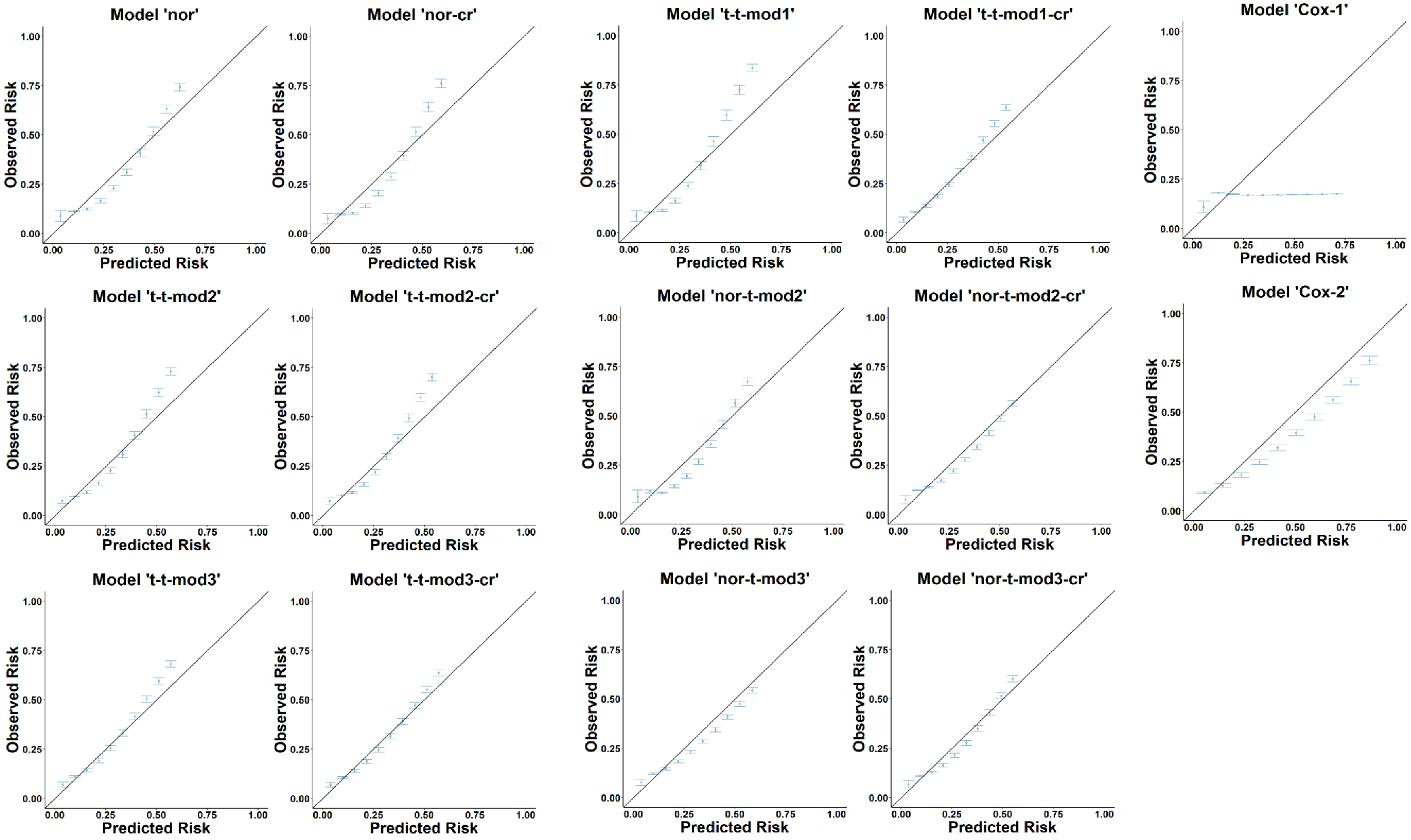

Figure S17. Calibration plots of all 12 joint models and the 2 Cox models in IDEAL trial PD testing dataset for landmark time 1.5 years, forecast horizon 1 year. Calibration plots compare predicted and observed mortality rates within deciles of risk score along with 95% confidence intervals. The number of patients at risk at selected landmark times and forecast horizons in IDEAL trial PD testing dataset, consisting of a total of 78 patients, are provided in main text.

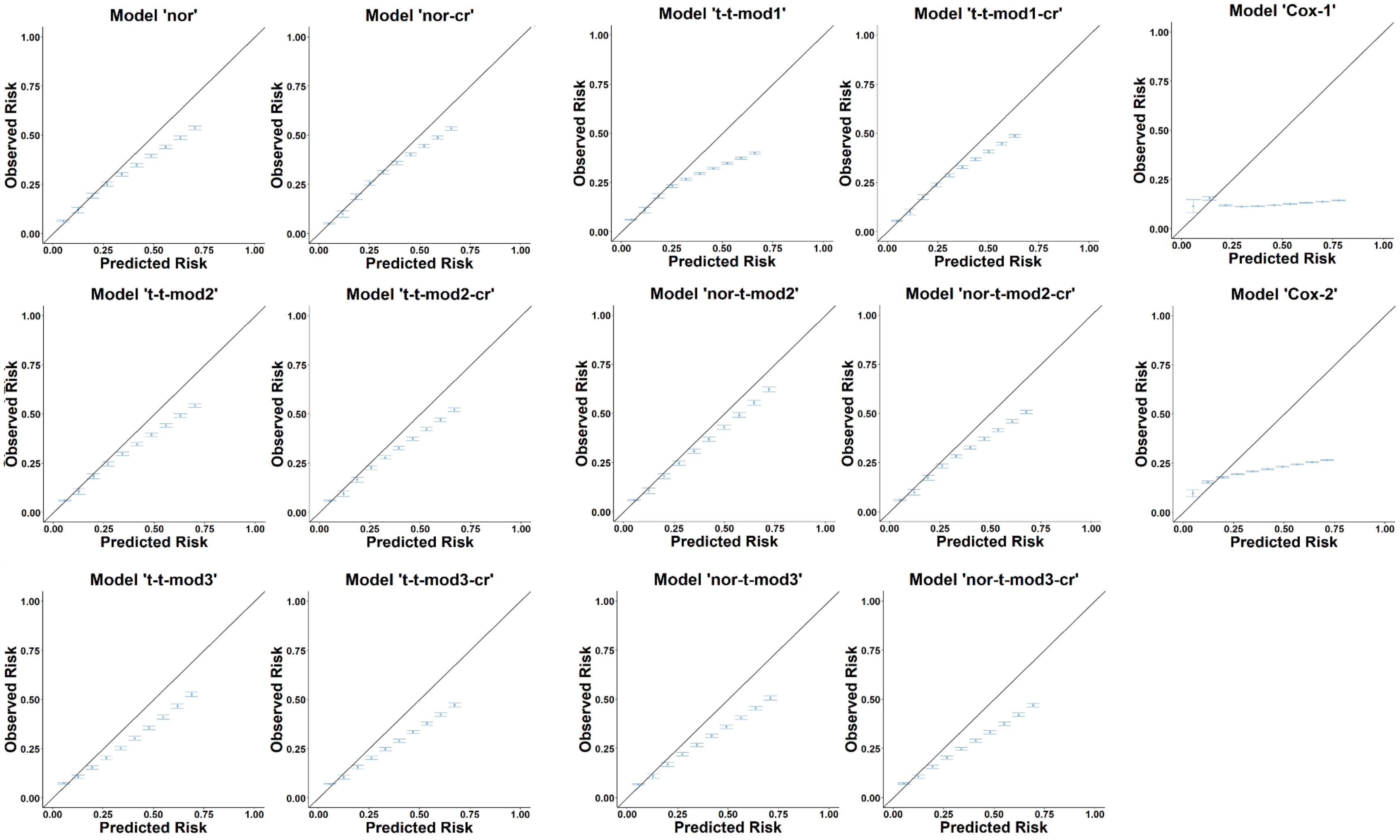

Figure S18. Calibration plots of all 12 joint models and the 2 Cox models in IDEAL trial PD testing dataset for landmark time 2 years, forecast horizon 1 year. Calibration plots compare predicted and observed mortality rates within deciles of risk score along with 95% confidence intervals. The number of patients at risk at selected landmark times and forecast horizons in IDEAL trial PD testing dataset, consisting of a total of 78 patients, are provided in main text.

3.9. Summary of Results from the Application of Novel JM to the HD Population of the IDEAL Trial

Baseline characteristics of patients in the IDEAL HD training dataset are presented in Table S14 below according to event status. Patients who died on HD are older (mean ± one standard deviation of 64.7 ± 9.7 years compared with 49.9 ± 12.4 and 58.2 ± 11.8 for transplanted and censored patients, respectively), with higher frequency of diabetes (54.8% versus 15.6% in transplanted patients) and cardiovascular disease comorbidities (61.3% versus 20% in transplanted patients) at baseline and lowest percentage of patients who never smoked (25.8% versus 51.1% in transplanted patients and 44.2% in censored patients). These results are well in line with the baseline risk variables selection procedure results, which identified age, cardiovascular disease, smoking status as well as use of statins medication at baseline from covariate selection procedure for time-to-event outcomes (Section 1.2) using the **My.stepwise** package in **R**. These baseline covariates represent established risk factors for morality in HD patients[37,38] as a confirmation for the variable selection procedure from prior literature. Baseline risk variables selected for the time to KTx sub-model almost matched the ones from PD analyses, namely, age, ethnicity and presence of diabetes comorbidity at baseline (smoking status was additionally included for the PD population) as a result of the contemporary trends in kidney recipients' characteristics for Australia and New Zealand at the time of IDEAL trial recruitment and conduct[39].

The baseline covariates in the albumin sub-model almost matched the ones in the survival sub-model, whereby age and body mass index, in addition to cardiovascular disease and intake of statins at baseline were considered. As a preliminary graphical check, Figure S19 demonstrates subject-specific longitudinal profiles of albumin against reverse time in patients from the IDEAL HD training dataset, stratified by event type. As before, blue lines indicate smoothed mean value from all patients in each group using LOWESS smoother. In line with the observations from the PD patients analyses, the trajectory of albumin declines steadily in patients, who pass away while on hemodialysis, suggesting again a potential significant inverse association between albumin trajectory and risk of all-cause mortality in patients treated with HD. Furthermore, one can observe the increase in albumin values in the final year before transplantation in patients who eventually receive kidney transplantation in the IDEAL HD training dataset. Thus, a potential positive association between longitudinal albumin and the occurrence of kidney transplantation could be investigated by the set of joint models that account for transplantation as a competing risk.

Table S14. Baseline patient characteristics of IDEAL trial HD training dataset (N=236) according to event type.

Characteristic	Total (N=236)	According to Event Type			
		Dead (N=62)	KTx (N=45)	Censored (N=129)	*P*-value
**Age (years)***	58.9 [50.4, 67.9]	64.7 ± 9.7	49.9 ± 12.4	58.2 ± 11.8	<.001
**Gender**					0.192
Female	62 (26.3%)	18 (29%)	7 (15.6%)	37 (28.7%)	
Male	174 (73.7%)	44 (71%)	38 (84.4%)	92 (71.3%)	
**Ethnicity**					0.061
Caucasian	159 (67.4%)	40 (64.5%)	37 (82.2%)	82 (63.6%)	
Non-Caucasian	77 (32.6%)	22 (35.5%)	8 (17.8%)	47 (36.4%)	
**BMI (kg/m^2)**	28.3 [24.6, 34.2]	27.1 [24.7,35.7]	28.1 [25.8,30.3]	29.4 [24.5,35.1]	0.574
**Primary kidney disease**					0.014
Diabetic nephropathy	88 (37.3%)	28 (45.2%)	7 (15.6%)	53 (41.1%)	
Hypertension / Renovascular disease	18 (7.6%)	5 (8.1%)	2 (4.4%)	11 (8.5%)	
Glomerulonephritis	52 (22.0%)	13 (21%)	10 (22.2%)	29 (22.5%)	
Polycystic kidney disease	20 (8.5%)	4 (6.5%)	8 (17.8%)	8 (6.2%)	
Other	58 (24.6%)	12 (19.4%)	18 (40%)	28 (21.7%)	
**Smoking status**					0.011
Never	96 (40.7%)	16 (25.8%)	23 (51.1%)	57 (44.2%)	
Current	39 (16.5%)	8 (12.9%)	6 (13.3%)	25 (19.4%)	
Former	101 (42.8%)	38 (61.3%)	16 (35.6%)	47 (36.4%)	
**Start of dialysis**					0.604
Early Start	103 (43.6%)	24 (38.7%)	23 (51.1%)	56 (43.4%)	
Late Start	133 (56.4%)	38 (61.3%)	22 (48.9%)	73 (56.6%)	
**Comorbidities**					
Diabetes mellitus	107 (45.3%)	34 (54.8%)	7 (15.6%)	66 (51.2%)	<.001
Hypertension	221 (93.6%)	57 (91.9%)	40 (88.9%)	124 (96.1%)	0.188
CVD	89 (37.7%)	38 (61.3%)	9 (20%)	42 (32.6%)	<.001
Hyperlipidaemia	150 (63.6%)	42 (67.7%)	26 (57.8%)	82 (63.6%)	0.572
Stoke/Davies Score	1.0 [0.0, 2.0]	1.5 [1.0,2.0]	0.0 [0.0,1.0]	1.0 [0.0,2.0]	<.001
**Laboratory parameters**					
Albumin (g/L)	38.0 [34.0, 41.0]	37.0 [32.0,39.0]	39.0 [36.0,42.0]	38.0 [34.0,41.0]	0.004
Creatinine (mmol/L)	11.0 [9.2, 13.5]	10.2 [8.1,11.9]	12.2 [10.1,14.9]	11.0 [9.5,13.7]	<.001
Urea (mmol/L)	5.9 [4.9, 7.1]	5.5 [4.8,6.6]	6.0 [5.2,7.8]	5.9 [4.9,7.4]	0.161
eGFR (mL/min/1.73m^2)	11.8 [9.7, 13.4]	12.1 [10.6,13.9]	11.5 [10.1,12.9]	11.8 [9.4,13.4]	0.621
Haemoglobin (g/L)*	111.0 [100.8, 121.2]	109.9 ± 15.4	112.5 ± 15.6	111.4 ± 18.1	0.726
White blood cell (x10^^{9}/L)	7.4 [5.9, 8.8]	7.4 [6.0,8.7]	7.1 [5.9,8.3]	7.5 [6.0,9.2]	0.247
Calcium (mmol/L)	2.3 [2.1, 2.4]	2.3 [2.2,2.4]	2.3 [2.1,2.4]	2.3 [2.1,2.4]	0.469
Phosphorus (mmol/L)	2.0 [1.6, 2.3]	2.0 [1.5,2.4]	1.9 [1.5,2.3]	1.9 [1.7,2.3]	0.770
Cholesterol (mmol/L)	4.2 [3.6, 5.1]	4.2 [3.5,5.4]	4.5 [3.9,5.1]	4.1 [3.5,5.0]	0.365
Triglycerides (mmol/L)	1.8 [1.3, 2.7]	2.0 [1.3,2.8]	1.8 [1.4,2.5]	1.8 [1.1,2.7]	0.783
Intact parathyroid hormone (pmol/L)**	29.1 [15.0, 55.9]	24.0 [11.0,52.0]	25.0 [14.0,39.0]	29.9 [17.0,56.3]	0.295

Table continued on next page

Table **S14 (continued).** Baseline patient characteristics of IDEAL trial HD training dataset (N=236) according to event type.

**Characteristic**	**Total (N=236)**	**According to Event Type**			
		**Dead (N=62)**	**KTx (N=45)**	**Censored (N=129)**	***P*-value**
**Medications**					
Angiotensin converting-enzyme inhibitor	92 (39.0%)	22 (35.5%)	16 (35.6%)	54 (41.9%)	0.609
Angiotensin II receptor blocker	45 (19.1%)	9 (14.5%)	11 (24.4%)	25 (19.4%)	0.431
Statin	153 (64.8%)	37 (59.7%)	27 (60%)	89 (69%)	0.339
Erythropoiesis stimulating agent	84 (35.6%)	23 (37.1%)	18 (40%)	43 (33.3%)	0.694

BMI: body mass index; CVD: cardiovascular disease (defined as a composite of ischemic heart disease, congestive heart failure and peripheral vascular disease); eGFR: estimated glomerular filtration rate (using the Cockcroft-Gault equation); KTx: kidney transplantation; N: number of patients

* Results for age and haemoglobin at baseline are presented as mean ± 1 standard deviation as these variables did not show deviation from normality as assessed with Shapiro-Wilk test. All other continuous variables showed deviation from normal distribution and were presented with median [interquartile range].

** Calculated after imputation of baseline data for 35 patients (14.8%) using the nearest K neighbours method with the **simputation** package in **R**.

Table S15. Baseline patient characteristics of IDEAL trial HD testing dataset (N=236) according to event type.

Characteristic	Total (N=79)	According to Event Type			
		Dead (N=16)	KTx (N=18)	Censored (N=45)	*P*-value
**Age (years)***	59.1 [51.3, 68.1]	66.8 [61.2,74.7]	53.8 [47.9,56.8]	59.1 [51.0,69.3]	<.001
**Gender**					0.117
Female	25 (31.6%)	7 (43.8%)	8 (44.4%)	10 (22.2%)	
Male	54 (68.4%)	9 (56.2%)	10 (55.6%)	35 (77.8%)	
**Ethnicity**					0.920
Caucasian	64 (81.0%)	13 (81.2%)	14 (77.8%)	37 (82.2%)	
Non-Caucasian	15 (19.0%)	3 (18.8%)	4 (22.2%)	8 (17.8%)	
**BMI (kg/m^2)**	28.6 [24.5, 33.5]	28.0 [24.6,34.0]	26.8 [24.3,31.9]	29.9 [25.7,33.6]	0.572
**Primary kidney disease**					0.004
Diabetic nephropathy	25 (31.6%)	10 (40.0%)	0 (0.0%)	15 (60.0%)	
Hypertension / Renovascular disease	8 (10.1%)	3 (37.5%)	1 (12.5%)	4 (50.0%)	
Glomerulonephritis	19 (24.1%)	1 (5.3%)	7 (36.8%)	11 (57.9%)	
Polycystic kidney disease	12 (24.1%)	1 (8.3%)	6 (50.0%)	5 (41.7%)	
Other	15 (19%)	1 (6.7%)	4 (26.7%)	10 (66.7%)	
**Smoking status**					0.484
Never	31 (39.2%)	4 (25%)	8 (44.4%)	19 (42.2%)	
Current	6 (7.6%)	2 (12.5%)	0 (0%)	4 (8.9%)	
Former	42 (53.2%)	10 (62.5%)	10 (55.6%)	22 (48.9%)	
**Start of dialysis**					0.242
Early Start	44 (55.7%)	9 (56.2%)	13 (72.2%)	22 (48.9%)	
Late Start	35 (44.3%)	7 (43.8%)	5 (27.8%)	23 (51.1%)	
**Comorbidities**					
Diabetes mellitus	31 (39.2%)	12 (75%)	0 (0%)	19 (42.2%)	<.001
Hypertension	79 (100%)	16 (100%)	18 (100%)	45 (100%)	NA
CVD	28 (35.4%)	14 (87.5%)	0 (0%)	14 (31.1%)	<.001
Hyperlipidaemia	42 (53.2%)	13 (81.2%)	5 (27.8%)	24 (53.3%)	0.008
Stoke/Davies Score	1.0 [0.0, 2.0]	2.0 [2.0,3.0]	0.0 [0.0,0.0]	1.0 [0.0,1.0]	<.001
**Laboratory parameters**					
Albumin (g/L)	39.0 [36.0, 42.0]	37.0 [34.5,42.0]	40.5 [37.0,42.0]	39.0 [37.0,43.0]	.192
Creatinine (mmol/L)	10.4 [8.6, 13.2]	8.3 [7.1,10.8]	10.4 [9.0,14.0]	11.7 [9.2,13.7]	.019
Urea (mmol/L)	5.7 [4.7, 6.6]	5.1 ± 1.6	6.2 ± 1.3	5.7 ± 1.3	.084
eGFR (mL/min/1.73m^2)	12.4 [10.4, 14.0]	13.6 ± 3.1	11.7 ± 2.2	11.9 ± 3.0	.115
Haemoglobin (g/L)*	110.0 [102.0, 122.0]	107.1 ± 11.9	117.2 ± 17.8	110.3 ± 13.2	.099
White blood cell (x10^^{9}/L)	7.0 [5.8, 8.4]	7.3 ± 1.9	6.6 ± 1.4	7.5 ± 2.1	.258
Calcium (mmol/L)	2.3 [2.2, 2.4]	2.3 ± 0.3	2.3 ± 0.2	2.3 ± 0.2	.493
Phosphorus (mmol/L)	1.8 [1.6, 2.2]	1.6 ± 0.4	1.9 ± 0.4	2.0 ± 0.5	.028
Cholesterol (mmol/L)	4.3 [3.6, 5.1]	3.8 [3.4,5.0]	5.0 [4.7,6.7]	4.1 [3.5,5.0]	.004
Triglycerides (mmol/L)	2.1 [1.4, 2.7]	2.4 [1.5,3.7]	2.2 [1.5,3.6]	2.0 [1.2,2.5]	.300
Intact parathyroid hormone (pmol/L)**	39.7 [18.3, 70.3]	36.0 [16.0,68.0]	40.4 [17.8,71.7]	39.7 [18.3,75.9]	.937

Table continued on next page

Table **S15 (continued).** Baseline patient characteristics of IDEAL trial HD testing dataset (N=236) according to event type.

Characteristic	Total (N=236)	According to Event Type			
		Dead (N=62)	KTx (N=45)	Censored (N=129)	*P*-value
**Medications**					
Angiotensin converting-enzyme inhibitor	31 (39.2%)	6 (37.5%)	10 (55.6%)	15 (33.3%)	0.261
Angiotensin II receptor blocker	18 (22.8%)	2 (12.5%)	4 (22.2%)	12 (26.7%)	0.509
Statin	49 (62.0%)	13 (81.2%)	9 (50%)	27 (60%)	0.339
Erythropoiesis stimulating agent	19 (24.1%)	4 (25%)	6 (33.3%)	9 (20%)	0.694

BMI: body mass index; CVD: cardiovascular disease (defined as a composite of ischemic heart disease, congestive heart failure and peripheral vascular disease); eGFR: estimated glomerular filtration rate (using the Cockcroft-Gault equation); KTx: kidney transplantation; N: number of patients; NA: not applicable

* Results for age and haemoglobin at baseline are presented as mean ± 1 standard deviation as these variables did not show deviation from normality as assessed with Shapiro-Wilk test. All other continuous variables showed deviation from normal distribution and were presented with median [interquartile range]. Haemoglobin baseline data for 3 patients (3.8%) were imputed using the nearest K neighbours method with the **simputation** package in **R**.

** Calculated after imputation of baseline data for 10 patients (12.7%) using the nearest K neighbours method with the **simputation** package in **R**.

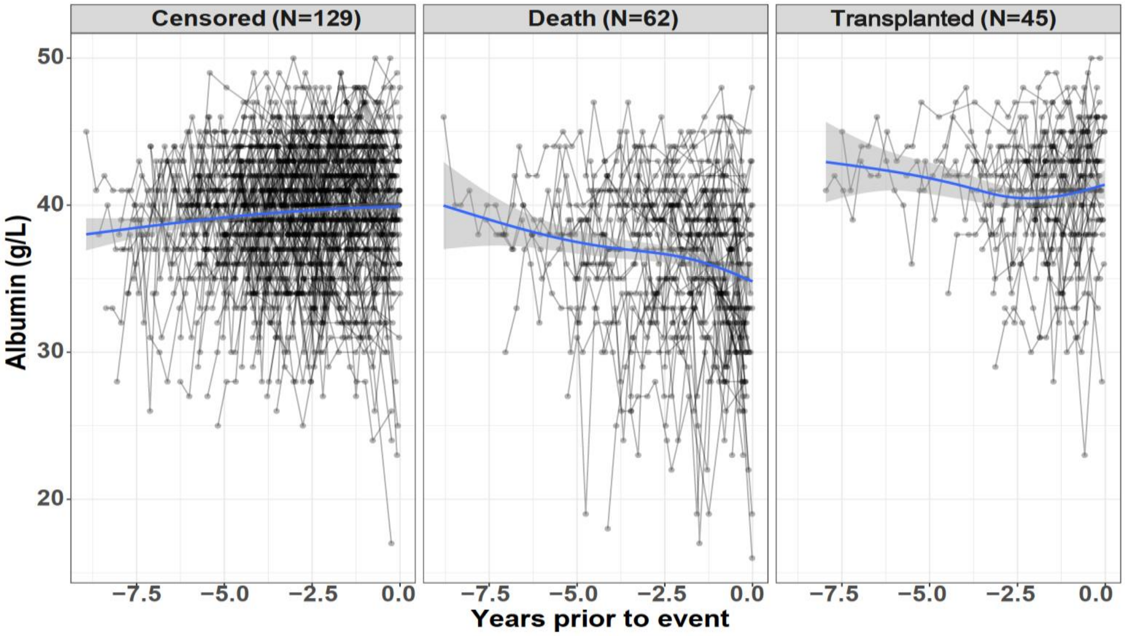

Figure S19. Albumin measurements in patients from the IDEAL trial HD training dataset in reverse time according to event type. Connected scatter plots (in grey) represent time trajectories of albumin for each individual obtained from recorded values during follow-up (each dot represents a different follow-up visit). Left panel contains 1855 individual albumin values from 129 patients, who were considered censored. Middle panel contains 670 individual albumin values from 62 patients, who passed away during study follow-up. Left panel contains 402 individual albumin values from 45 patients, who had KTx. The blue lines indicate the LOWESS smoothing curve. N: number of patients.

As noted in Section 3.1, the construction of JM involves the selection of the association between albumin trajectory and the survival sub-model as a first step. An initial JM consisted of a linear mixed effects model for albumin and a survival sub-model under a parametric baseline hazard under the Weibull PH specification as introduced in Section 1.3.1. All baseline covariates obtained from covariate selection procedure were included in the longitudinal and survival sub-models. The model selection results in terms of the WAIC values and rank according to WAIC are presented in Table S16 below with a total of six association functions. The relative rankings of the alternative association structures are almost a perfect copy of the results with the PD training dataset in main text. Thus, the current value link function is again used for further JM analyses in this Section. Visual diagnostics of the longitudinal sub-model for albumin trajectory fitted as part of the standard JM ('*nor-nor*') were performed. The left panel of Figure S20 displays a plot of Residuals vs. Fitted values, suggesting that the linear trend adequately represents the longitudinal albumin data. However, the right panel's quantile-quantile plot of standardised residuals reveals significant deviations from Gaussian (normal) assumptions. These deviations suggest the presence of outliers in albumin values, though pinpointing their origin - whether from individual extremes (*b*-outliers), intra-individual irregularities (*e*-outliers), or a combination, is challenging. Consequently, various robust joint models, encompassing diverse outlier structures detailed in Section 1.3.2, are relevant for patients treated with HD from the IDEAL trial. To highlight the time patterns of occurrence of all-cause mortality and KTx, Figure S21 plots the cumulative incidence curves of the two competing events. The shape of the curves is similar to the one from the PD trial dataset, justifying the applicability of the cause-specific Weibull PH hazard model in HD patients, too. However, under a similar follow-up of over 8 years, the death event rate in the HD population is comparatively lower than in the PD population (26% vs 35%) with a greater time-to-event (2.8 vs 2 years) suggesting lower observed incidence of all-cause mortality occurring at later time periods after dialysis start. Both the rate and median time-to-event for KTx in the HD training dataset (19% and 2 years, respectively) were comparable with ones from the PD training dataset (15% and 1.93 years respectively). While event rates of 26% and 19% for the main outcome and the competing risk in a training dataset of 236 patients could pose difficulties to estimation of JM with competing risks under classical frequentist methods, our estimation procedure utilising the latest Bayesian algorithms resulted in excellent convergence of all models considered (i.e. R-hat for all parameters is well below the recommended threshold of 1.1 for Bayesian estimation, similarly to what was shown in Tables S8,S9 from PD analyses).

Table S16. Comparison of JM fits with various alternative link structures between albumin and survival sub-models for IDEAL trial HD training dataset.

**Model**	**WAIC**	**Rank**	**Estimation time (min)**
Current value	15129.87	1	7
Current slope*	15166.3	5	6.5
Current value + Current slope*	15144.46	3	12.6
Cumulative effects (area under the curve of the linear predictor)*	15166.39	6	49
Shared random effects (b_0)**	15148.51	4	5.2
Shared random effects (b_0, b_1)**	15133.09	2	3.9

* Estimated with '*stan_jm*' function as part of **rstanarm** package in **R**.

** Estimated with a custom-built program, which was created for the purposes of this study as an extension of the '*stan_jm*' function of the **rstanarm** package in **R**.

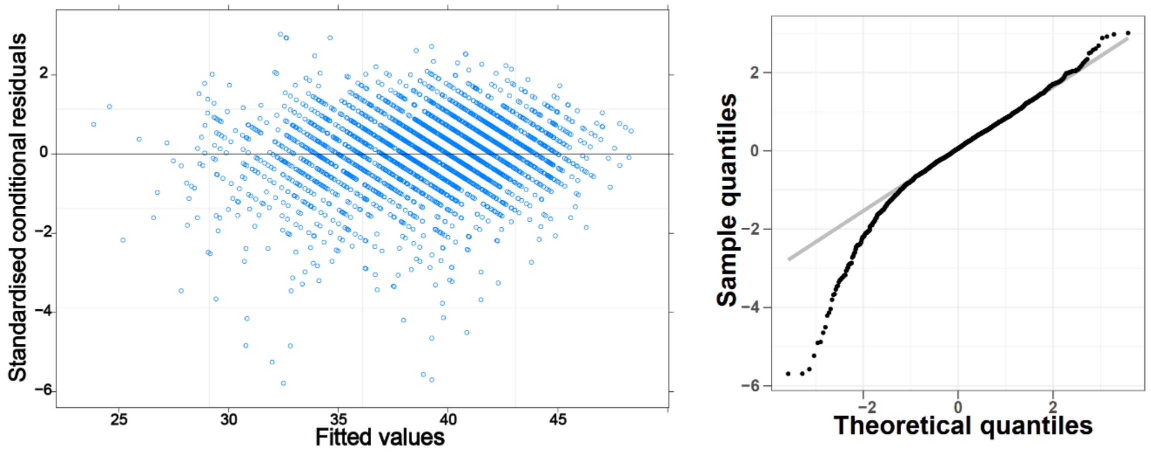

Figure S20. Visual inspection of the standardised conditional residuals obtained from the standard JM with Gaussian assumptions (‘*nor-nor’*) fitted to the IDEAL HD training dataset. Left panel: standardised conditional residuals vs fitted values; right panel: quantile-quantile (Q-Q) plot of standardised conditional residuals against standard Normal distribution. Both panels are plotted using all 2927 values of standardised conditional residuals for albumin as well as 2927 albumin fitted values for the 236 patients in the IDEAL HD training dataset.

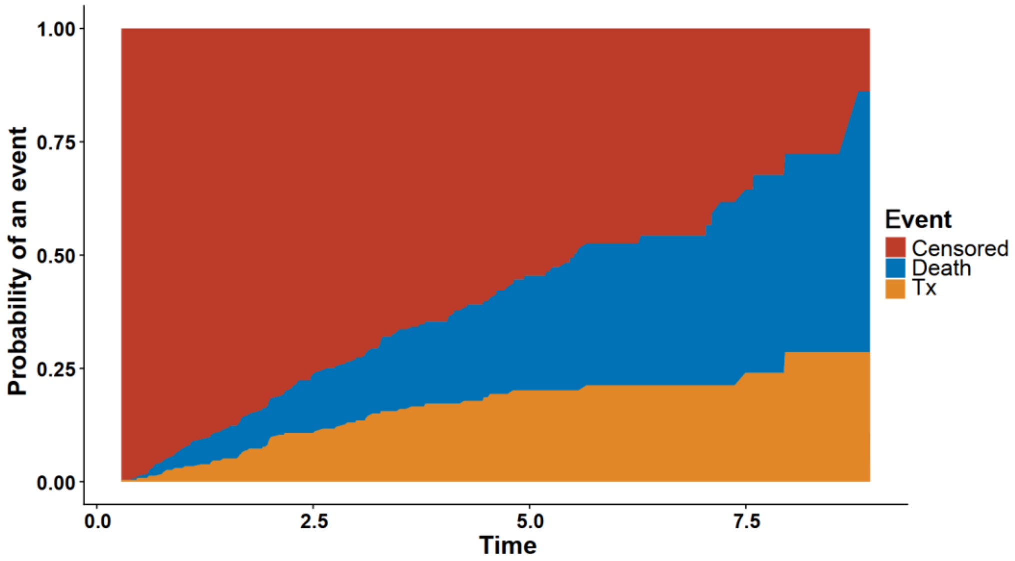

Figure S21. Cumulative incidence plot of competing events in the IDEAL HD training dataset. The plot was calculated using time-to-event data from all 236 patients in the IDEAL HD training dataset.

As with PD patients analyses, we first present results from the six JM without considering competing events, i.e., kidney transplantation is first considered as a censoring event. Table S15 provides posterior summaries of the standard and robust JM parameters and Table 3 in main text shows corresponding WAIC values and times for computation using our innovative software solution we discussed in the preceding sections. The relative rankings of the robust JM agree very well with the results from PD analyses. Again, the best fitting model is ‘*t-t-mod3*’, followed by ‘*nor-t-mod3*’, while the standard JM under normal

distribution assumptions is ranked last. Thus, the result that model '*t-t-mod3*', which assumes the heavy-tailed *t*-distribution for both random effects and error structure, best fits the clinical trial dataset, is an indication for the presence of both *b*- and *e*-outliers in albumin trajectories in HD patients, too. This impression is confirmed by the posterior means (95% credible interval) of the degrees of freedom parameters ϕ and δ of 3.95, (2.29, 8.15) and 3.56, (3.06, 4.13), respectively, whose low estimates support the adequacy of the heavy tailed *t*-distributions to model albumin trajectories. The relative rankings of the models are unaffected by the adjustment for the competing risk of KTx (Table 3 in main text) and such adjustment to the time-to-event sub-model specification results in little changes of parameter estimates from their counterparts treating KTx as right-censored. Thus, robust modelling approaches are warranted in HD regardless of the time-to-event sub-model structure.

From Table S15 one can see that the hazard ratio estimate is little affected by the specification of outlier structure and underlying distributional assumptions, with estimates for a unit decrease in albumin of 1.19 to 1.20. Adjusting for the competing risk of KTx (Table S17) does not affect these estimates allowing for a direct parallel with the application of the robust JM with and without competing risks in the PD population, in which little sensitivity of albumin HR estimates for death was observed, too. Therefore, the results in the HD population provide evidence for the inverse negative relationship between longitudinal albumin values and all-cause mortality, whereby a unit decrease in albumin value is associated with a 1.19- to 1.20-fold increase in the risk of death, which is of similar magnitude to HR estimates from analyses with the PD population (1.22 to 1.27). Interestingly, model specifications in Table S16 suggest a significant positive association between the occurrence of KTx and longitudinal albumin, so that a unit increase in albumin value is estimated to result in a 1.17- to 1.20-fold increase the occurrence of KTx. This is in line with the observation from preliminary graphical analysis in Figure S19 and in contrast with findings from the PD population, where no such significant relationship was observed. It is important to highlight that all baseline risk factors in the KTx sub-models demonstrate a significant association, which underscores the effectiveness of the covariate selection process and suggests that the comprehensive list of variables also offers valuable insights into the occurrence of competing events in the HD population, too.

Table S17. Posterior summaries of the JM parameters fitted to the IDEAL HD training dataset ignoring competing risks.

Parameter	nor-nor	t-t-mod1	t-t-mod2	nor-t-mod2	t-t-mod3	nor-t-mod3
	Mean (95% CI) | P-value*	Mean (95% CI) | P-value*	Mean (95% CI) | P-value*	Mean (95% CI) | P-value*	Mean (95% CI) | P-value*	Mean (95% CI) | P-value*
**Albumin sub-model**						
Intercept	45.67, (42.73, 48.61) | <0.001	46.68, (43.70, 49.60) | <0.001	46.10, (43.16, 49.03) | <0.001	45.54, (42.73, 48.46) | <0.001	47.14, (44.34, 50.04) | <0.001	45.86, (43.00, 48.88) | <0.001
Time on HD	0.24, (0.07, 0.41) | 0.004	0.34, (0.21, 0.48) | <0.001	0.33, (0.19, 0.46) | <0.001	0.31, (0.17, 0.45) | <0.001	0.35, (0.22, 0.47) | <0.001	0.31, (0.17, 0.44) | <0.001
Age**	-0.09, (-0.13, -0.05) | <0.001	-0.09, (-0.13, -0.05) | <0.001	-0.09, (-0.13, -0.06) | <0.001	-0.09, (-0.13, -0.05) | <0.001	-0.10, (-0.13, -0.06) | <0.001	-0.09, (-0.13, -0.05) | <0.001
BMI (kg/m^2)**	-0.06, (-0.12, -0.00) | 0.043	-0.09, (-0.15, -0.03) | 0.004	-0.07, (-0.13, -0.01) | 0.024	-0.06, (-0.12, -0.00) | 0.040	-0.09, (-0.14, -0.03) | 0.005	-0.07, (-0.13, -0.01) | 0.020
CVD**	-2.02, (-2.99, -1.04) | <0.001	-1.81, (-2.78, -0.78) | <0.001	-2.03, (-3.06, -1.02) | <0.001	-2.07, (-3.07, -1.07) | <0.001	-1.80, (-2.80, -0.82) | <0.001	-2.09, (-3.08, -1.15) | <0.001
Statin**	1.17, (0.29, 2.12) | 0.009	1.17, (0.25, 2.05) | 0.011	1.11, (0.16, 2.05) | 0.024	1.13, (0.20, 2.09) | 0.017	1.16, (0.27, 2.04) | 0.012	1.15, (0.24, 2.05) | 0.016
Σ(1,1)	10.92, (8.68, 13.51) | <0.001	8.98, (7.02, 11.27) | <0.001	8.54, (5.92, 11.60) | <0.001	10.61, (8.44, 13.06) | <0.001	6.63, (4.65, 9.18) | <0.001	10.30, (8.31, 12.77) | <0.001
Σ(1,2)	-0.38, (-1.05, 0.22) | 0.231	-0.20, (-0.68, 0.24) | 0.390	-0.12, (-0.58, 0.29) | 0.596	-0.11, (-0.64, 0.37) | 0.698	0.00, (-0.36, 0.34) | 0.964	0.08, (-0.41, 0.54) | 0.737
Σ(2,2)	0.97, (0.63, 1.41) | <0.001	0.54, (0.34, 0.81) | <0.001	0.46, (0.26, 0.75) | <0.001	0.58, (0.36, 0.87) | <0.001	0.35, (0.19, 0.57) | <0.001	0.52, (0.32, 0.78) | <0.001
γ***	—	6.34, (4.79, 8.26) | <0.001	—	—	—	—
ϕ***	—	—	11.97, (3.26, 48.39) | <0.001	—	3.95, (2.29, 8.15) | <0.001	—
σ	2.85, (2.77, 2.93) | <0.001	2.34, (2.22, 2.47) | <0.001	2.29, (2.16, 2.43) | <0.001	2.29, (2.16, 2.43) | <0.001	1.97, (1.87, 2.08) | <0.001	1.98, (1.86, 2.09) | <0.001
δ***	—	—	4.64, (3.59, 5.95) | <0.001	4.59, (3.56, 5.85) | <0.001	3.56, (3.06, 4.13) | <0.001	3.48, (2.93, 4.09) | <0.001
**Survival sub-model hazard ratios**						
Age**	1.05, (1.02, 1.08) | 0.002	1.05, (1.02, 1.08) | 0.001	1.05, (1.02, 1.08) | <0.001	1.05, (1.02, 1.08) | <0.001	1.05, (1.02, 1.08) | 0.001	1.05, (1.02, 1.08) | <0.001
CVD**	1.46, (0.85, 2.58) | 0.183	1.45, (0.84, 2.52) | 0.190	1.47, (0.86, 2.51) | 0.149	1.46, (0.86, 2.54) | 0.166	1.46, (0.85, 2.52) | 0.175	1.47, (0.85, 2.58) | 0.165
Smoking status** (Former vs Never)	1.91, (1.05, 3.57) | 0.033	1.90, (1.08, 3.43) | 0.025	1.89, (1.06, 3.48) | 0.031	1.90, (1.06, 3.55) | 0.029	1.94, (1.09, 3.61) | 0.022	1.92, (1.07, 3.51) | 0.030
Smoking status** (Current vs Never)	1.42, (0.57, 3.38) | 0.431	1.41, (0.56, 3.29) | 0.430	1.41, (0.56, 3.30) | 0.433	1.43, (0.59, 3.35) | 0.408	1.36, (0.56, 3.15) | 0.461	1.40, (0.56, 3.21) | 0.439
Statin**	0.68, (0.40, 1.17) | 0.153	0.68, (0.39, 1.16) | 0.161	0.68, (0.41, 1.17) | 0.153	0.68, (0.41, 1.17) | 0.159	0.68, (0.41, 1.15) | 0.149	0.68, (0.40, 1.18) | 0.167
Albumin****	1.20, (1.13, 1.28) | <0.001	1.20, (1.12, 1.28) | <0.001	1.19, (1.12, 1.27) | <0.001	1.19, (1.12, 1.27) | <0.001	1.19, (1.12, 1.28) | <0.001	1.19, (1.11, 1.27) | <0.001
Weibull shape	1.71, (1.38, 2.08) | <0.001	1.72, (1.38, 2.07) | <0.001	1.71, (1.37, 2.06) | <0.001	1.71, (1.38, 2.06) | <0.001	1.72, (1.39, 2.07) | <0.001	1.71, (1.37, 2.08) | <0.001

BMI: Body mass index; CI: credible interval; CVD: cardiovascular disease; HD: hemodialysis.

* Posterior means are given along with 95% credible intervals. P-values were calculated based on the posterior distribution tail probabilities for containing the zero value.

** Values taken at baseline.

*** γ denotes the degrees of freedom parameter from *t*-distribution for both random and residual error applicable to '*t-t-mod1*' only; ϕ and δ denote the degrees of freedom parameter for *t*-distributed random effects for random effects and residual error, respectively.

**** Hazard ratio per 1 g/L decrease in albumin is showed.

Table S18. Posterior summaries of the JM parameters fitted to the IDEAL HD training dataset accounting for competing risks.

Parameter	nor-nor-cr<br>Mean (95% CI) | P-value*	t-t-mod1-cr<br>Mean (95% CI) | P-value*	t-t-mod2-cr<br>Mean (95% CI) | P-value*	nor-t-mod2-cr<br>Mean (95% CI) | P-value*	t-t-mod3-cr<br>Mean (95% CI) | P-value*	nor-t-mod3-cr<br>Mean (95% CI) | P-value*
**Albumin sub-model**						
Intercept	45.70, (42.82, 48.73) | <0.001	46.68, (43.69, 49.59) | <0.001	46.23, (43.17, 49.24) | <0.001	45.65, (42.67, 48.68) | <0.001	47.14, (44.29, 49.99) | <0.001	45.83, (42.85, 48.85) | <0.001
Time on HD	0.27, (0.10, 0.44) | 0.002	0.36, (0.23, 0.50) | <0.001	0.35, (0.21, 0.48) | <0.001	0.33, (0.19, 0.48) | <0.001	0.36, (0.24, 0.50) | <0.001	0.33, (0.19, 0.46) | <0.001
Age**	-0.09, (-0.13, -0.05) | <0.001	-0.09, (-0.13, -0.05) | <0.001	-0.09, (-0.13, -0.06) | <0.001	-0.09, (-0.13, -0.05) | <0.001	-0.10, (-0.13, -0.06) | <0.001	-0.09, (-0.13, -0.05) | <0.001
BMI (kg/m^2) **	-0.07, (-0.13, -0.00) | 0.037	-0.09, (-0.15, -0.03) | 0.004	-0.07, (-0.13, -0.02) | 0.016	-0.07, (-0.13, -0.00) | 0.040	-0.09, (-0.15, -0.03) | 0.004	-0.07, (-0.13, -0.00) | 0.034
CVD**	-2.04, (-3.02, -1.06) | <0.001	-1.81, (-2.79, -0.82) | <0.001	-2.03, (-3.08, -1.01) | <0.001	-2.08, (-3.08, -1.08) | <0.001	-1.85, (-2.77, -0.88) | <0.001	-2.05, (-3.04, -1.11) | <0.001
Statin**	1.15, (0.22, 2.05) | 0.016	1.14, (0.26, 1.98) | 0.010	1.14, (0.20, 2.06) | 0.018	1.11, (0.16, 2.06) | 0.021	1.17, (0.26, 2.05) | 0.011	1.15, (0.25, 2.03) | 0.014
Σ(1,1)	10.90, (8.73, 13.56) | <0.001	8.91, (7.04, 11.25) | <0.001	8.38, (5.90, 11.19) | <0.001	10.62, (8.47, 13.12) | <0.001	6.63, (4.61, 9.13) | <0.001	10.30, (8.27, 12.76) | <0.001
Σ(1,2)	-0.38, (-1.07, 0.25) | 0.250	-0.19, (-0.68, 0.25) | 0.417	-0.10, (-0.55, 0.31) | 0.647	-0.09, (-0.64, 0.40) | 0.724	-0.00, (-0.36, 0.32) | 0.980	0.08, (-0.41, 0.54) | 0.714
Σ(2,2)	1.02, (0.66, 1.48) | <0.001	0.56, (0.35, 0.83) | <0.001	0.46, (0.26, 0.74) | <0.001	0.59, (0.37, 0.88) | <0.001	0.37, (0.20, 0.59) | <0.001	0.54, (0.33, 0.82) | <0.001
γ***	—	5.26, (4.09, 6.72) | <0.001	—	—	—	—
Φ***	—	—	8.87, (3.23, 23.95) | <0.001	—	3.78, (2.27, 7.45) | <0.001	—
Σ	2.85, (2.77, 2.93) | <0.001	2.34, (2.21, 2.47) | <0.001	2.29, (2.16, 2.43) | <0.001	2.29, (2.16, 2.42) | <0.001	1.97, (1.87, 2.08) | <0.001	1.97, (1.87, 2.08) | <0.001
δ***	—	—	4.67, (3.60, 5.97) | <0.001	4.59, (3.52, 5.85) | <0.001	3.58, (3.06, 4.18) | <0.001	3.46, (2.99, 3.98) | <0.001
**Survival sub-model hazard ratios**						
Age**	1.05, (1.02, 1.08) | 0.001	1.05, (1.02, 1.08) | 0.001	1.05, (1.02, 1.08) | <0.001	1.05, (1.02, 1.08) | <0.001	1.05, (1.02, 1.08) | <0.001	1.05, (1.02, 1.08) | <0.001
CVD**	1.45, (0.86, 2.56) | 0.174	1.47, (0.85, 2.53) | 0.175	1.48, (0.86, 2.56) | 0.173	1.47, (0.86, 2.50) | 0.154	1.45, (0.85, 2.52) | 0.181	1.48, (0.86, 2.54) | 0.151
Smoking status** (Former vs Never)	1.91, (1.07, 3.60) | 0.030	1.92, (1.06, 3.62) | 0.031	1.90, (1.07, 3.53) | 0.031	1.91, (1.07, 3.51) | 0.027	1.95, (1.12, 3.57) | 0.016	1.91, (1.08, 3.54) | 0.034
Smoking status** (Current vs Never)	1.42, (0.58, 3.28) | 0.415	1.42, (0.58, 3.32) | 0.428	1.41, (0.58, 3.32) | 0.439	1.43, (0.56, 3.44) | 0.442	1.37, (0.57, 3.04) | 0.466	1.39, (0.57, 3.24) | 0.448
Statin**	0.68, (0.40, 1.15) | 0.144	0.67, (0.40, 1.16) | 0.147	0.68, (0.41, 1.16) | 0.152	0.68, (0.41, 1.15) | 0.151	0.68, (0.40, 1.18) | 0.178	0.68, (0.40, 1.16) | 0.154
Albumin****	1.20, (1.13, 1.28) | <0.001	1.19, (1.12, 1.27) | <0.001	1.19, (1.12, 1.27) | <0.001	1.19, (1.12, 1.28) | <0.001	1.19, (1.12, 1.27) | <0.001	1.19, (1.11, 1.27) | <0.001
Weibull shape	5.48, (3.94, 7.85) | <0.001	5.61, (4.01, 8.12) | <0.001	5.52, (3.91, 7.92) | <0.001	5.52, (3.95, 7.85) | <0.001	5.54, (3.96, 7.97) | <0.001	5.51, (3.97, 7.91) | <0.001

Table continued on next page

Table S18 (Continued). Posterior summaries of the JM parameters fitted to the IDEAL HD training dataset accounting for competing risks.

Parameter	nor-nor-cr	t-t-mod1-cr	t-t-mod2-cr	nor-t-mod2-cr	t-t-mod3-cr	nor-t-mod3-cr
	Mean (95% CI) | P-value*	Mean (95% CI) | P-value*	Mean (95% CI) | P-value*	Mean (95% CI) | P-value*	Mean (95% CI) | P-value*	Mean (95% CI) | P-value*
**Transplantation sub-model hazard ratios**						
Age**	0.98, (0.95, 1.00) | 0.055	0.98, (0.95, 1.00) | 0.042	0.98, (0.95, 1.00) | 0.055	0.98, (0.95, 1.00) | 0.063	0.98, (0.95, 1.00) | 0.070	0.98, (0.95, 1.00) | 0.069
Ethnicity (Caucasian vs Non-Caucasian)	2.44, (1.13, 5.85) | 0.022	2.42, (1.10, 5.76) | 0.027	2.43, (1.12, 5.70) | 0.027	2.45, (1.11, 5.94) | 0.024	2.50, (1.13, 5.91) | 0.022	2.49, (1.16, 5.81) | 0.016
Diabetes**	0.38, (0.14, 0.89) | 0.023	0.37, (0.15, 0.87) | 0.025	0.37, (0.14, 0.87) | 0.022	0.37, (0.14, 0.87) | 0.020	0.38, (0.15, 0.88) | 0.022	0.37, (0.15, 0.85) | 0.016
Albumin*****	1.18, (1.07, 1.32) | 0.002	1.17, (1.05, 1.31) | 0.003	1.18, (1.06, 1.32) | <0.001	1.18, (1.06, 1.32) | 0.004	1.20, (1.08, 1.34) | <0.001	1.19, (1.08, 1.34) | <0.001
Weibull shape	3.28, (2.43, 4.53) | <0.001	3.30, (2.45, 4.63) | <0.001	3.28, (2.44, 4.60) | <0.001	3.28, (2.44, 4.58) | <0.001	3.25, (2.41, 4.56) | <0.001	3.25, (2.40, 4.56) | <0.001

BMI: Body mass index; CI: credible interval; CVD: cardiovascular disease; HD: hemodialysis.

* Posterior means are given along with 95% credible intervals. P-values were calculated based on the posterior distribution tail probabilities for containing the zero value.

** Values taken at baseline.

*** γ denotes the degrees of freedom parameter from *t*-distribution for both random and residual error applicable to '*t-t-mod1*' only; ϕ and δ denote the degrees of freedom parameter for *t*-distributed random effects for random effects and residual error, respectively.

**** Hazard ratio per 1 g/L decrease in albumin is showed.

***** Hazard ratio per 1 g/L increase in albumin is showed.

The above findings for the relevance of albumin trajectory for both all-cause mortality and KTx endpoints in HD patients encourage the application of considered JM for the dynamic prediction of death in the testing dataset. The total size of the IDEAL HD testing dataset trial considered in this study is 79 patients. Table S19 shows frequencies of subjects who were at risk, subjects who passed away or were transplanted, and who were censored within the forecast horizons at the selected landmark times of years 1, 1.5 and 2 following start of HD and the forecast horizons of 6 months, 1 year, 1.5 years and 2 years, respectively.

Table S19. Patients at risk at selected landmark times and forecast horizons in IDEAL HD testing dataset N=79). The able shows number of patients (and associated percentages) who were at risk, died, had kidney transplantation (KTx) or were censored at the respective prediction horizons and landmark times.

	At risk	Death	KTx	Censored
**Forecast horizon 6 months**				
LMT = 1 year	75	2 (2.7%)	3 (4%)	0 (0%)
LMT = 1.5 years	70	4 (5.7%)	0 (0%)	3 (4.3%)
LMT = 2 years	63	1 (1.6%)	3 (4.8%)	1 (1.6%)
**Forecast horizon 1 year**				
LMT = 1 year	75	6 (8%)	3 (4%)	3 (4%)
LMT = 1.5 years	70	5 (7.1%)	3 (4.3%)	4 (5.7%)
LMT = 2 years	63	4 (6.3%)	5 (7.9%)	2 (3.2%)
**Forecast horizon 1.5 years**				
LMT = 1 year	75	7 (9.3%)	6 (8%)	4 (5.3%)
LMT = 1.5 years	70	8 (11.4%)	5 (7.1%)	5 (7.1%)
LMT = 2 years	63	5 (7.9%)	8 (12.7%)	4 (6.3%)
**Forecast horizon 2 years**				
LMT = 1 year	75	10 (13.3%)	8 (10.7%)	5 (6.7%)
LMT = 1.5 years	70	9 (12.9%)	8 (11.4%)	7 (10%)
LMT = 2 years	63	6 (9.5%)	9 (14.3%)	9 (14.3%)

As performed with the PD population, the predictive performance of all 12 JM discussed as part of this study compared with simpler and more traditional approaches to risk prediction, such as the Cox model with baseline albumin ('*Cox-1*') and the extended Cox model in which longitudinal albumin is considered as a time-varying covariate measured without error and constant between follow-ups ('*Cox-2*'). Both of the Cox models also included the set of baseline risk factors included in survival sub-models of the JM (as per Tables S15 and S16). We provide AUC and BS values from dynamic predictions at the considered landmark times 1, 1.5 and 2 years of HD treatment for future time horizons until 5 years of dialysis treatment at increments of 6 months in Table 5 in main text and Table S20, respectively.

Table S20. Brier score (BS) of dynamic predictions for three landmark times and various prediction horizons with IDEAL HD testing dataset.

Landmark time (Year)	Forecast Time (Year)	Cox-1	Cox-2	nor	nor-cr	t-t-mod1	t-t-mod1-cr	t-t-mod2	t-t-mod2-cr	nor-t-mod2	nor-t-mod2-cr	t-t-mod3	t-t-mod3-cr	nor-t-mod3	nor-t-mod3-cr
1	1.5	0.03	0.02	0.02	0.02	0.02	0.02	0.02	0.02	0.02	0.02	0.02	0.02	0.02	0.03
	2	0.08	0.07	0.07	0.07	0.07	0.07	0.07	0.07	0.07	0.07	0.07	0.07	0.07	0.07
	2.5	0.08	0.08	0.08	0.07	0.08	0.08	0.08	0.08	0.08	0.08	0.08	0.07	0.08	0.07
	3	0.11	0.11	0.10	0.10	0.10	0.10	0.10	0.10	0.11	0.10	0.10	0.10	0.10	0.10
	3.5	0.12	0.11	0.11	0.10	0.11	0.10	0.11	0.10	0.11	0.10	0.11	0.10	0.11	0.10
	4	0.13	0.12	0.11	0.10	0.11	0.10	0.11	0.10	0.11	0.10	0.11	0.10	0.11	0.10
	4.5	0.13	0.11	0.11	0.10	0.11	0.10	0.11	0.10	0.12	0.10	0.11	0.10	0.11	0.10
	5	0.15	0.12	0.11	0.10	0.12	0.10	0.11	0.10	0.12	0.10	0.11	0.10	0.12	0.10
1.5	2	0.06	0.05	0.05	0.05	0.05	0.05	0.05	0.05	0.05	0.05	0.05	0.05	0.05	0.05
	2.5	0.07	0.06	0.06	0.06	0.06	0.06	0.06	0.06	0.06	0.06	0.06	0.06	0.06	0.06
	3	0.10	0.10	0.09	0.09	0.09	0.09	0.09	0.09	0.09	0.09	0.09	0.09	0.09	0.09
	3.5	0.11	0.12	0.10	0.10	0.10	0.10	0.10	0.10	0.10	0.10	0.11	0.10	0.10	0.10
	4	0.12	0.14	0.11	0.10	0.11	0.10	0.11	0.10	0.11	0.10	0.11	0.10	0.11	0.10
	4.5	0.13	0.15	0.11	0.10	0.11	0.10	0.11	0.10	0.11	0.10	0.11	0.10	0.11	0.10
	5	0.14	0.17	0.12	0.10	0.12	0.10	0.11	0.10	0.12	0.11	0.12	0.10	0.12	0.10
2	2.5	0.02	0.02	0.02	0.02	0.02	0.02	0.02	0.02	0.02	0.02	0.02	0.02	0.02	
	3	0.06	0.06	0.06	0.05	0.06	0.05	0.06	0.05	0.06	0.05	0.06	0.06	0.06	0.06
	3.5	0.07	0.08	0.08	0.07	0.07	0.07	0.07	0.07	0.07	0.07	0.08	0.07	0.08	0.07
	4	0.08	0.10	0.09	0.08	0.09	0.08	0.09	0.08	0.09	0.08	0.10	0.09	0.10	0.09
	4.5	0.10	0.12	0.10	0.09	0.10	0.09	0.10	0.09	0.10	0.09	0.10	0.09	0.10	0.09
	5	0.11	0.14	0.11	0.09	0.11	0.09	0.11	0.09	0.11	0.09	0.11	0.10	0.12	0.10

The accuracy results for predictions of all-cause mortality in the HD testing dataset follow closely the main patterns observed with JM for dynamic prediction in PD patients. Firstly, prediction discrimination increases with longer follow-up as assessed by higher AUC values at later landmark times. As reasoned before, this is a consequence of the greater ability of proposed JM to better estimate the individual underlying albumin trajectory as more follow-up measurements are collected resulting in more accurate predictions of all-cause mortality (or, equivalently, survival). Secondly, adjustment for the competing risk of KTx provides better discrimination as well as calibration of predictions (as judged by lower and thus better Brier score values) for almost all landmark and prediction horizons for each of the investigated albumin outlier structures. Thus, the significant association between longitudinal albumin and the occurrence of transplantation, combined with the adjustment for cumulative incidences of transplantations conditionally to the overall survival up to the respective landmark time, proves beneficial for the accuracy of survival dynamic predictions in the testing HD dataset. Thirdly, both Cox models are outperformed by all considered JM across all landmark times and forecast horizons in terms of both discrimination and calibration aspects of prediction accuracy. As with PD analyses, the '*Cox-2*' with time-varying albumin as a covariate along with the other included baseline risk factors (age, cardiovascular disease, smoking status as well as use of statins medication at baseline) is an improvement over the '*Cox-1*' model with baseline albumin and the remaining baseline risk factors, once gain underscoring the relevance of longitudinal albumin for more accurate survival predictions. However, the weakness of '*Cox-2*' is that it implicitly assumes the 'Last Value Carried Forward' imputation since the last available albumin value is treated as constant in future horizons. Instead, presented JM incorporate individual patient variability and potential *b*- and *e*-outliers in albumin trajectory based on all collected information to predict the individual path of future albumin, resulting in consistently higher prediction accuracy than Cox models, whereby the difference is increasing the longer the forecast horizon. Overall, almost all predictions from JM models across the various landmark times and forecast horizons in Table 5 could be classified as 'excellent' (i.e., in the interval for AUC of 0.80-0.89) with some 'outstanding' results (AUC of 0.90 and above), which demonstrate an equal, if not superior prediction accuracy than what was observed in the PD patients analyses.

4. Simulation Studies

4.1. Simulation Study to Assess Robust JM without Competing Risks

Hereby, the focus is placed on testing and validation of the proposed software solution in addition to investigating the effects of outliers at the patient level (i.e. patients within the population do not conform to population trends), as well as outlying observations within an individual's set of measurements, on the estimation of parameters for the proposed joint modelling approaches in the first six rows of Table 1 from main text. Hence, the time-to-event sub-model consists of a single outcome (e.g. death) without competing risks.

The JM data were simulated using the inverse transform sampling method as proposed previously in JM literature[40]. The simulation set-up was influenced by the IDEAL PD training dataset. A sample size of $N = 236$ individuals was considered with 200 datasets being simulated under '*t-t-mod3*' as the data generating model. This is selected as the best fitting model to the IDEAL PD training dataset when ignoring competing risks. More precisely, the longitudinal sub-model is given by:

$$
\begin{aligned}
Y_{ij} &= \mathrm{Y}_i^*(t_{ij}) + Z_{ij} \\
&= \alpha_1 + \alpha_2 t_{ij} + b_{1i} + b_{2i} t_{ij} + Z_{ij}\,, \qquad (25)
\end{aligned}
$$

where, similarly to IDEAL trial design, each individual has a record every 3 months during the first 3 years of follow-up, and then every 6 months until year 6 of follow up. A model incorporating both random intercept $(\alpha_1 + b_{1i})$ and random slope $(\alpha_2 + b_{2i})$ was adopted to match albumin longitudinal sub-model from analyses with IDEAL dataset, which is also the prevailing approach observed in the joint modelling discourse. As motivated by IDEAL trial results from model, '*t-t-mod3*', the assumed structure for the random effects and residual error involved parameters $\phi = 6$ and $\delta = 4$ and the true values for α_1, α_2 and σ were set to 25, -0.7 and 2.5, respectively. In addition, the entries of the variance-covariance matrix $\mathbf{\Sigma}$ were similarly motivated by the corresponding estimates of the '*t-t-mod3*' model: $\mathbf{\Sigma}_{11} = 9$, $\mathbf{\Sigma}_{12} = 0$ and $\mathbf{\Sigma}_{22} = 2$. Survival data were generated from the following model:

$$
h_i(t) = h_0(t)\exp\left(\omega_1 + \omega_2 X + \eta \mathrm{Y}_i^*(t)\right)\,, \qquad (26)
$$

where $X \sim Normal(0,1)$ as baseline continuous risk covariate, $h_0(t)$ is specified by a Weibull baseline hazard under proportional hazards, that is, $h_0(t) = \lambda t^{\lambda - 1}$, where λ is the shape parameter, and the intercept ω_1 represents the log of the scale parameter ν as explained in Section 1.2.1 as follows from Weibull PH baseline hazards model. Here, $\lambda = 2.2$ and log-scale $\log(\nu) = \omega_1 = 1.2$. The log-hazard ratio (log-HR) for the continuous baseline risk factor $\omega_2$was set to 1 and the log-HR for longitudinal biomarker η were set equal to -0.2 motivated by estimate for the association between longitudinal albumin and death from IDEAL dataset. The censoring time for each individual (assuming right censoring), was generated from a Weibull distribution with shape 3.5 and scale 4.5.

Under these simulation parameters, the structure of the simulated datasets closely mimics the characteristics of the observed IDEAL PD training dataset. Table S21 provides a comparison between the simulated data and the IDEAL PD training dataset ($N = 236$). As can be concluded from the table, the characteristics of the simulated data closely match the patterns observed in the real data used to fit the JM in terms of the number (and rate) of patients with the event, the median time to event and the median number of albumin records per patient, both for patients who had the event (death) as well as censored patients (treating the

events of transfer to HD and KTx as censoring events). This demonstrates adequacy of the data generation process to simulate complex JM data.

Table S21. Comparison of dataset characteristics between IDEAL PD training dataset used for model fitting and simulated data.

**Characteristic**	**IDEAL PD Training Dataset (N = 236)**	**Simulation Data* (N = 236)**
Number of dead patients (rate)	82 (35%)	86 (36%)
Median time to event, dead patients (years)	2.03	2.05
Number of records per patient, dead patients	8	9
Number of censored patients (rate)	154 (65%)	150 (64%)
Median time to event, censored patients (years)	2.41	2.39
Number of records per patient, censored patients	9	10

* Median values from 200 simulated datasets are shown.

All JM specifications without competing risks were fitted to each of the simulated datasets (i.e. standard JM with Gaussian assumptions for random effects and residual error, '*nor-nor*' as well as suggested robust joint models, '*t-t-mod1*', '*t-t-mod2*', '*nor-t-mod2*', '*t-t-mod3*' and '*nor-t-mod3*'). Our purpose-built program in Stan was used for Bayesian inference with two parallel chains. For models '*nor-nor*', '*t-t-mod1*', '*nor-t-mod2*' and '*nor-t-mod3*', each chain was of length 2000 with the first 1000 iterations considered as warm-up and not used for inference. For models '*t-t-mod2*' and '*t-t-mod3*', which have more parameters and of greater model complexity, each chain was of length 3000 with the first 1500 as warm-up and discarded for inference. Convergence for each simulated dataset was addressed by ensuring that each parameter had an estimated R-hat statistic less than 1.1.

The assessment of the capability to accurately estimate the true parameter values was conducted using a well-established procedure and metrics from literature on simulation studies with joint models under Bayesian inference[8]. For the model that was fit to the d-th simulated dataset ($d = 1, \dots, 200$) the following estimates were calculated:

- The mean of the posterior distribution for parameter p, denoted as $\widehat{\Theta}_p^{(d)}$
- The bias for parameter p, denoted as $\widehat{\mathrm{B}}_p^{(d)} = \widehat{\Theta}_p^{(d)} - \Theta_p$, where Θ_p denotes the true parameter value that was used to simulate the data
- The relative bias for parameter p, denoted as $\widehat{\mathrm{R}}_p^{(d)} = \Theta_k^{-1}\left(\widehat{\Theta}_p^{(d)} - \Theta_p\right)$, where Θ_p denotes the true parameter value that was used to simulate the data
- The standard deviation of the posterior distribution (i.e. estimated standard error) for parameter p, denoted as $\widehat{\mathrm{S}}_p^{(d)}$

For inference in the simulation study, the following quantities were then calculated using the estimates obtained across the $D = 200$ datasets:

- The mean bias for parameter p, defined as $\overline{\mathrm{B}}_p = \frac{1}{D}\sum_{d=1}^{D} \widehat{\mathrm{B}}_p^{(d)}$ (referred to as "mean bias")
- The mean relative bias for parameter p, defined as $\overline{\mathrm{R}}_p = \frac{1}{D}\sum_{d=1}^{D} \widehat{\mathrm{R}}_p^{(d)}$ (referred to as "mean relative bias")

- The mean standard deviation of the posterior distribution for parameter p, defined as $\bar{\mathrm{S}}_p = \frac{1}{D}\sum_{d=1}^{D} \hat{\mathrm{S}}_p^{(d)}$ (referred to as "mean estimated standard error")
- The standard error of the posterior mean for parameter p, defined as the standard deviation of the estimates $\left\{ \hat{\Theta}_p^{(d)}; d = 1, \ldots, D \right\}$ and denoted as $\mathrm{sd}(\hat{\Theta}_p)$ (referred to as "empirical standard error")

Table S22 presents the results of the simulation showing, for each of the 6 model specifications, the estimated mean bias ($\bar{\mathrm{B}}_p$), mean relative bias ($\bar{\mathrm{R}}_p$), mean estimated standard error ($\bar{\mathrm{S}}_p$), and empirical standard error ($\mathrm{sd}(\hat{\Theta}_p)$) for each of the parameters, as applicable. Of note, parameter γ belongs to '*t-t-mod1*' model only and its bias and relative bias are not presented as the truth is unknown (since structure from model '*t-t-mod3*' is used to simulate the data). Analogically, results for parameter ϕ are absent for models '*nor-nor*', '*t-t-mod1*', '*nor-t-mod2*' and '*nor-t-mod3*' and results for parameter δ were not calculated for models '*nor-nor*' and '*t-t-mod1*'. To allow comparison of variance-covariance matrix of the random effects, $\mathbf{\Sigma}$, between the standard JM for which both random effects and residual error follow Normal distribution assumptions (*'nor-nor'*) as well as models that have normally distributed random effects ('*nor-t-mod2'*, '*nor-t-mod3*'), with the remaining models, the relationship $\mathbf{\Sigma} \times \frac{\phi}{\phi-2}$ was used as the true variance-covariance matrix of the random effects simulated under the multivariate t-distribution as per '*t-t-mod3*' model. Similarly, the true residual error σ for model '*nor-nor*' was considered as $\sigma \times \frac{\delta}{\delta-2}$.

Overall, the results from the simulation study suggest that the proposed estimation program was able to recover the true parameter values used in the data generating model. Considering results from the true data generating model, '*t-t-mod3*', it can be seen that relative bias of the parameters were in the range of 1% to 2%, whereby the mean estimated standard error (i.e. the mean standard deviation for the posterior distribution) was close to the empirical standard error for all parameters, with one exception. The posterior mean from 200 replicates for parameter ϕ was 7.85 compared with a true value of 6, resulting in a mean relative bias of 31%. However, that positive bias is unlikely to influence the inferences from the model. It is important to highlight that the parameter governs the degree of outliers within the longitudinal random effects, that is, to what extent some individuals do not conform to population trends (baseline albumin value and/or rate of decline of albumin over time). A low value, such as 6 as estimated from '*t-t-mod3*' model with IDEAL trial PD training dataset, suggests an actual presence of outlying patients. In principle, as the value approaches infinity one would expect the random effects to converge to normal distribution. Usually in practice, values above the value of 30 are considered indicative of normal distribution. Hence, a posterior mean of 7.85 across the simulations still strongly suggests the presence of outlying patients as per simulation design.

All other models, which were in fact mis-specified according to the simulation design, demonstrated similar results for the longitudinal and event sub-models, with some major deviations under the '*t-t-mod1*' model. Concerning longitudinal sub-model parameters, all models gave a near-zero bias for α_1 and α_2. Looking at random effects variance-covariance parameters, mean relative bias was negligible (between -3% to 1%) for all models, except for model '*t-t-mod1*', which demonstrated mean relative bias of 26% and 27% for $\mathbf{\Sigma}_{11}$ and $\mathbf{\Sigma}_{22}$ parameters, which highlights that the unrealistic assumption that the mixing variable is equal for both random effects and error components leads to inaccuracies in variances of random effects. As the data was simulated under uncorrelated random effects (motivated by IDEAL trial PD results in Table S7), all models have yielded near-zero posterior mean values for the $\mathbf{\Sigma}_{12}$ parameter. Interestingly, models *'t-t-mod1*', '*t-t-mod2*' and '*nor-t-mod2*' overestimated residual error (mean relative bias 23%-25%) which is a result of the assumed dependence between Z_{ij} and Z'_{ij} , which is overcome by both the '*t-t-mod3*' and '*nor-*

t-mod3', which operate under the true residual error structure in simulated data. Perhaps due to the same reason, the degree of freedom parameter for the error term, δ, both '*t-t-mod2*' and '*nor-t-mod2*' severely overestimated it (mean relative bias of 131% and 126%, respectively), whereby models '*t-t-mod3*' and '*nor-t-mod3*' resulted in minimal bias for the parameter (2%) with the mean estimated standard error nearly matching the empirical standard error.

As part of the event sub-model, all models with normal random effects structure of the longitudinal biomarker resulted in a mild upward bias in the intercept ω_1 (log-scale parameter of the Weibull PH baseline hazard) with mean relative bias of 9% to 11% under '*nor-nor*', '*nor-t-mod2*' and '*nor-t-mod3*', however, all models estimated the shape parameter of the Weibull baseline hazard (denoted by λ) quite well (mean relative bias between 1% and 2%). The log hazard ratio (log-HR) corresponding to the continuous baseline risk factor, ω_2, was estimated with negligible bias (mean relative bias of 1%) with all models. Importantly, the log-HR corresponding to the longitudinal biomarker, η, was measured quite accurately across all models, with mean relative bias of 5% for model '*nor-t-mod2'*, 4% for '*nor-nor*' and '*nor-t-mod2*' and 3% for models '*t-t-mod1*', '*t-t-mod2*', '*t-t-mod3*', respectively. Thus, the simulation confirms the empirical results obtained from the IDEAL PD training dataset, that even in the presence of *b*- and *e*-outliers in a longitudinal parameter linked with a time-to-event outcome, to the extent observed in a real clinical trial data with peritoneal dialysis patients, the standard JM as well as other (and potentially mis-specified robust joint models) might capture the true relationship between the longitudinal biomarker and survival.

Table S22. Simulation results based on data assuming '*t-t-mod3*' model structure and absence of competing risks.

Parameter	True Value	nor-nor			t-t-mod1			t-t-mod2			nor-t-mod2			t-t-mod3			nor-t-mod3		
		$\hat{\Theta}$	$\bar{B}_p, \bar{R}_p$	$\bar{S}_p, sd(\hat{\Theta})_p$	$\hat{\Theta}$	$\bar{B}_p, \bar{R}_p$	$\bar{S}_p, sd(\hat{\Theta})_p$	$\hat{\Theta}$	$\bar{B}_p, \bar{R}_p$	$\bar{S}_p, sd(\hat{\Theta})_p$	$\hat{\Theta}$	$\bar{B}_p, \bar{R}_p$	$\bar{S}_p, sd(\hat{\Theta})_p$	$\hat{\Theta}$	$\bar{B}_p, \bar{R}_p$	$\bar{S}_p, sd(\hat{\Theta})_p$	$\hat{\Theta}$	$\bar{B}_p, \bar{R}_p$	$\bar{S}_p, sd(\hat{\Theta})_p$
α_1	25	25.02	0.02, 0	0.27, 0.28	25.02	0.02, 0	0.25, 0.28	25	0, 0	0.25, 0.27	25.01	0.01, 0	0.26, 0.28	25	0, 0	0.24, 0.26	25.01	0.01, 0	0.26, 0.28
α_2	-0.7	-0.7	0, -0.01	0.16, 0.14	-0.69	0.01, -0.01	0.15, 0.14	-0.7	0, 0	0.15, 0.14	-0.69	0.01, -0.02	0.15, 0.14	-0.7	0, 0	0.14, 0.13	-0.69	0.01, -0.01	0.15, 0.13
$\Sigma(1,1)^*$	9	13.62	0.12, 0.01	1.6, 2.01	11.36	2.36, 0.26	1.44, 1.51	9.13	0.13, 0.01	1.58, 1.47	13.34	-0.16, -0.01	1.57, 1.96	9.14	0.14, 0.02	1.52, 1.44	13.54	0.04, 0	1.52, 1.95
$\Sigma(1,2)^{**}$	0	0.01	0.01, —	0.67, 0.75	0.01	0.01, —	0.57, 0.58	0.06	0.06, —	0.48, 0.47	0.12	0.12, —	0.64, 0.69	0.04	0.04, —	0.46, 0.46	0.03	0.03, —	0.62, 0.75
$\Sigma(2,2)^*$	2	3	0, 0	0.49, 0.59	2.54	0.54, 0.27	0.44, 0.46	2.04	0.04, 0.02	0.43, 0.44	2.89	-0.11, -0.04	0.47, 0.56	2.05	0.05, 0.03	0.42, 0.42	2.99	-0.01, 0	0.46, 0.56
γ^{***}	—	—	—	—	10.24	—	1.78, 2.24	—	—	—	—	—	—	—	—	—	—	—	—
ϕ	6	—	—	—	—	—	—	8.17	2.17, 0.36	5.14, 4.32	—	—	—	7.85	1.85, 0.31	4.64, 3.96	—	—	—
σ^{****}	2.5	3.54	0, 0	0.06, 0.15	3.12	0.62, 0.25	0.08, 0.08	3.07	0.57, 0.23	0.08, 0.08	3.07	0.57, 0.23	0.08, 0.08	2.5	0, 0	0.07, 0.07	2.51	0.01, 0	0.07, 0.07
δ	4	—	—	—	—	—	—	9.23	5.23, 1.31	1.64, 2.07	9.05	5.05, 1.26	1.6, 2.08	4.07	0.07, 0.02	0.39, 0.39	4.07	0.07, 0.02	0.39, 0.4
ω_1	1.2	1.31	0.11, 0.09	0.64, 0.7	1.26	0.06, 0.05	0.64, 0.72	1.26	0.06, 0.05	0.63, 0.74	1.34	0.14, 0.11	0.65, 0.7	1.25	0.05, 0.04	0.61, 0.72	1.31	0.11, 0.09	0.63, 0.73
ω_2	1	1.01	0.01, 0.01	0.14, 0.14	1.01	0.01, 0.01	0.14, 0.14	1	0, 0	0.13, 0.14	1.01	0.01, 0.01	0.14, 0.14	1.01	0.01, 0.01	0.13, 0.13	1.01	0.01, 0.01	0.13, 0.13
η	-0.2	-0.21	-0.01, 0.04	0.03, 0.03	-0.21	-0.01, 0.03	0.03, 0.03	-0.21	-0.01, 0.03	0.03, 0.03	-0.21	-0.01, 0.05	0.03, 0.03	-0.21	-0.01, 0.03	0.03, 0.03	-0.21	-0.01, 0.04	0.03, 0.03
λ	2.2	2.24	0.04, 0.02	0.2, 0.19	2.24	0.04, 0.02	0.2, 0.19	2.24	0.04, 0.02	0.2, 0.18	2.24	0.04, 0.02	0.2, 0.18	2.24	0.04, 0.02	0.2, 0.18	2.23	0.03, 0.01	0.2, 0.18

* For models '*nor-nor*', '*nor-t-mod2*' and '*nor-t-mod3*', true values for these parameters were considered as $\Sigma(1,1) = 9*(6/(6\text{-}2)) = 13.5$, $\Sigma(2,2) = 2*(6/(6\text{-}2)) = 3$.

** Since true $\Sigma(1,2) = 0$, relative bias for this parameter is not defined.

*** This parameter belongs to model '*t_t_mod1*' only. Since data were simulated under '*t-t-mod3*' model and the truth is unknown, $\bar{B}_p$, $\bar{R}_p$, $\bar{S}_p$ and $sd(\hat{\Theta})_p$ were not calculated.

**** For model '*nor-nor*', true value was considered as $\sigma = \sqrt{2.5^2 * (4/(4-2))} = 3.54$.

4.2. Simulation Study to Assess Robust JM with Competing Risks

A second simulation study was performed to test and validate the proposed software solution as well as investigate the effects of outliers and competing risks. It extends the simulation study from the previous Section using identical longitudinal model set-up and parameter values; however, the event sub-model consists of a competing risks cause-specific Weibull specification. The algorithm for simulating JM data with outliers and competing risks presented in Section 2.1 was utilised. Again, sample size of $N = 236$ individuals was considered with 200 datasets being simulated under '*t-t-mod3-cr*' as the data generating model. This is selected as the best fitting model to the IDEAL PD training dataset when adjusting for competing risks. For the time-to-event sub-model, the following cause-specific risks structure with Weibull PH was assumed:

$$h_{ik}(t) = h_{0k}(t)\exp\left(\omega_{1k} + \omega_{2k}X + \eta_k Y_i^*(t_{ij})\right), \qquad (27)$$

where $K = 3$ to match IDEAL dataset analysis with $k = 1$ for death from all causes while on peritoneal dialysis, and $k = 2$ and $k = 3$ corresponding to the competing events of transfer to HD and KTx, respectively, and $X \sim Normal(0,1)$ is a simulated baseline continuous risk covariate where $\omega_{21} = \omega_{22} = \omega_{23} = 1$. As motivated by results from IDEAL PD training dataset (Table S8), there was no significant association between longitudinal albumin and either transfer to HD or KTx, hence association parameters were set as $\eta_2 = \eta_3 = 0$, whereby $\eta_1 = -0.2$ in line with empirical results for the association between longitudinal albumin and death in the presence of competing risks. The cause-specific shape parameter from the Weibull baseline hazard model was set as follows: $\lambda_1 = 1.7, \lambda_2 = 1.3, \lambda_3 = 1.2$ and the log-scale parameter true values were: $\omega_{11} = 1.9$, $\omega_{12} = -2.5$ and $\omega_{13} = -3.1$. The censoring time for each individual (assuming right censoring), was generated from a Weibull distribution with shape 3.5 and scale 4.5.

Under these simulation parameters, the characteristics of the simulated data closely match the patterns observed in the real data (Table S23 below) in terms of the number (and rate) of patients with the event, the median time to event and the median number of albumin records per patient, both for patients who had the main event of interest (death), the first competing event (transfer to HD), the second competing event (KTx) as well as censored patients (patients who dropped out without experiencing any of the three events). Hence, a realistic simulation experimental set-up is used, which is very close to the observed data characteristics of the training IDEAL dataset reflecting the appropriate parameters for the simulation of JM data under both competing risks and outliers in the longitudinal biomarker.

Table S23. Comparison of dataset characteristics between IDEAL PD training dataset used for model fitting and simulated data accounting for competing risks.

Characteristic	IDEAL Training Dataset (N = 236)	Simulation Data* (N = 236)
**Death**		
Number of patients (rate)	82 (35%)	78 (33%)
Median time to event (years)	2.03	1.82
Number of records per patient	8	8
**Transfer to HD**		
Number of patients (rate)	58 (25%)	65 (28%)
Median time to event (years)	1.56	1.31
Number of records per patient	7	6
**KTx**		
Number of patients (rate)	35 (15%)	33 (14%)
Median time to event (years)	1.93	1.23
Number of records per patient	7	6
**Censored**		
Number of patients (rate)	61 (26%)	60 (25%)
Median time to event (years)	3.8	3.45
Number of records per patient	13	14

* Median values from 200 simulated datasets are shown. KTx: kidney transplantation

All JM allowing for competing risks as specified in Table 1 from main text were fitted to each of the simulated datasets. Our purpose-built program in Stan was used for Bayesian inference with two parallel chains. Each chain was of length 2500 with the first 1000 iterations considered as warm-up and not used for inference. Convergence for each simulated dataset was addressed by ensuring that each parameter had an estimated R-hat statistic less than 1.1. The assessment of the capability to accurately estimate the true parameter values was conducted using a well-established procedure and metrics as defined in Section 2.1.

Results from the simulation study are presented in Table S24. As far as the longitudinal sub-model estimates and the survival-sub model results are concerned, one can find similar patterns to those observed in the first simulation without competing risks. That is an encouraging finding, as in the second simulation the number of parameters has increased due to the addition of competing risks sub-models, however, the size of the dataset remains as before, suggesting the adequacy of the proposed estimation procedure even in moderately sized clinical trial datasets. Furthermore, all new parameters from transfer to HD and KTx sub-models ($\omega_{12}, \omega_{22}, \eta_2, \lambda_2, \omega_{13}, \omega_{23}, \eta_3, \lambda_3$) were recovered with negligible bias and mean estimated standard errors is close to the empirical standard errors. Again, the log-HR corresponding to the longitudinal biomarker, η_1, was measured quite accurately across all models, with mean relative bias of between 3% and 5%. Thus, our second simulation confirms the empirical results obtained from the IDEAL PD training dataset, that even in the presence of b- and e-outliers in a longitudinal parameter linked with a time-to-event outcome competing with two other events, the standard JM (‘*nor-nor-cr*’) as well as other (and potentially mis-specified robust joint models) might capture the true relationship between the longitudinal biomarker and survival just as well as the true and most complex robust JM (‘*t-t-mod3-cr’*). Also, all models suggest no relationship between the longitudinal biomarker and the competing events, with corresponding log-HR parameters η_2 and η_3 close to 0 with minimal bias.

As an additional note, simulations in the above two sections include fewer baseline covariates than the IDEAL dataset and we hereby outline the rationale for this design choice. A single continuous baseline covariate is sufficient for this purpose. In joint models, the likelihood factorizes over covariates, so adding independent predictors enlarges the fixed-effect block but does not change the mechanisms by which outliers or competing risks stress estimation. With our outlier-robust priors and Hamiltonian Monte Carlo, inference acts primarily through the random-effects distributions and likelihood gradients rather than the number of fixed effects. We therefore expect additional baseline covariates to be neutral with respect to bias, coverage, and convergence. This expectation is consistent with Simulation 4.3 below, whereby the algorithms comfortably handle a rich slate of baseline variables and nearly recreates the full IDEAL covariate profile within its data-generating mechanism for dynamic prediction.

Table S24. Simulation results based on data assuming *'t-t-mod3-cr'* model structure in the presence of competing risks.

Parameter	True Value	nor-nor-cr			t-t-mod1-cr			t-t-mod2-cr			nor-t-mod2-cr			t-t-mod3-cr			nor-t-mod3-cr		
		$\hat{\theta}$	$\bar{B}_p, \bar{R}_p$	$\bar{S}_p, sd(\hat{\theta})_p$	$\hat{\theta}$	$\bar{B}_p, \bar{R}_p$	$\bar{S}_p, sd(\hat{\theta})_p$	$\hat{\theta}$	$\bar{B}_p, \bar{R}_p$	$\bar{S}_p, sd(\hat{\theta})_p$	$\hat{\theta}$	$\bar{B}_p, \bar{R}_p$	$\bar{S}_p, sd(\hat{\theta})_p$	$\hat{\theta}$	$\bar{B}_p, \bar{R}_p$	$\bar{S}_p, sd(\hat{\theta})_p$	$\hat{\theta}$	$\bar{B}_p, \bar{R}_p$	$\bar{S}_p, sd(\hat{\theta})_p$
α_1	25	25.01	0.01 , 0	0.29 , 0.31	25.02	0.02 , 0	0.28 , 0.3	24.98	-0.02 , 0	0.27 , 0.29	25.01	0.01 , 0	0.29 , 0.3	25	0 , 0	0.27 , 0.29	25.01	0.01 , 0	0.28 , 0.3
α_2	-0.7	-0.7	0 , 0	0.17 , 0.15	-0.7	0 , 0	0.16 , 0.15	-0.7	0 , 0	0.16 , 0.15	-0.7	0 , -0.01	0.16 , 0.15	-0.7	0 , 0	0.15 , 0.14	-0.69	0.01 , -0.02	0.16 , 0.15
$\Sigma(1,1)$*	11	16.63	0.13 , 0.01	1.91 , 2.34	13.75	2.75 , 0.25	1.72 , 1.75	11.05	0.05 , 0	1.89 , 1.71	16.19	-0.31 , -0.02	1.87 , 2.24	11.23	0.23 , 0.02	1.86 , 1.71	16.39	-0.11 , -0.01	1.81 , 2.34
$\Sigma(1,2)$**	0	0.05	0.05 , —	0.77 , 0.88	0.04	0.04 , —	0.65 , 0.64	0.06	0.06 , —	0.55 , 0.49	0.13	0.13 , —	0.74 , 0.76	0.04	0.04 , —	0.54 , 0.48	0.06	0.06 , —	0.72 , 0.81
$\Sigma(2,2)$*	2	3.04	0.04 , 0.01	0.52 , 0.71	2.6	0.6 , 0.3	0.46 , 0.51	2.06	0.06 , 0.03	0.45 , 0.47	2.98	-0.02 , -0.01	0.5 , 0.66	2.1	0.1 , 0.05	0.44 , 0.47	3.03	0.03 , 0.01	0.49 , 0.64
γ ***	—	—	—	—	10.29	—	1.89 , 2.29	—	—	—	—	—	—	—	—	—	—	—	—
ϕ	6	—	—	—	—	—	—	7.75	1.75 , 0.29	4.52 , 3.8	—	—	—	8.1	2.1 , 0.35	4.84 , 4.31	—	—	—
σ****	2.5	3.54	0 , 0	0.06 , 0.16	3.12	0.62 , 0.25	0.08 , 0.08	3.08	0.58 , 0.23	0.08 , 0.08	3.08	0.58 , 0.23	0.08 , 0.08	2.51	0.01 , 0	0.08 , 0.08	2.51	0.01 , 0	0.08 , 0.07
δ	4	—	—	—	—	—	—	9.24	5.24 , 1.31	1.75 , 2.21	9.08	5.08 , 1.27	1.7 , 2.15	4.08	0.08 , 0.02	0.4 , 0.41	4.06	0.06 , 0.01	0.4 , 0.38
ω_{11}	1.9	2.01	0.11 , 0.06	0.65 , 0.7	1.98	0.08 , 0.04	0.64 , 0.71	1.94	0.04 , 0.02	0.62 , 0.66	2.04	0.14 , 0.07	0.65 , 0.69	1.91	0.01 , 0.01	0.61 , 0.68	1.99	0.09 , 0.05	0.63 , 0.68
ω_{21}	1	1.02	0.02 , 0.02	0.15 , 0.16	1.02	0.02 , 0.02	0.15 , 0.16	1.02	0.02 , 0.02	0.15 , 0.16	1.02	0.02 , 0.02	0.15 , 0.16	1.02	0.02 , 0.02	0.15 , 0.16	1.02	0.02 , 0.02	0.15 , 0.16
η_1	-0.2	-0.21	-0.01 , 0.04	0.03 , 0.03	-0.21	-0.01 , 0.03	0.03 , 0.03	-0.21	-0.01 , 0.03	0.03 , 0.03	-0.21	-0.01 , 0.05	0.03 , 0.03	-0.2	0 , 0.02	0.03 , 0.03	-0.21	-0.01 , 0.03	0.03 , 0.03
ω_{12}	-2.5	-2.45	0.05 , -0.02	0.73 , 0.79	-2.47	0.03 , -0.01	0.72 , 0.79	-2.47	0.03 , -0.01	0.72 , 0.81	-2.44	0.06 , -0.02	0.73 , 0.77	-2.46	0.04 , -0.01	0.71 , 0.75	-2.43	0.07 , -0.03	0.72 , 0.76
ω_{22}	1	1.03	0.03 , 0.03	0.15 , 0.17	1.03	0.03 , 0.03	0.15 , 0.17	1.02	0.02 , 0.02	0.15 , 0.17	1.02	0.02 , 0.02	0.15 , 0.16	1.02	0.02 , 0.02	0.15 , 0.17	1.03	0.03 , 0.03	0.15 , 0.17
η_2 **	0	0	0 , —	0.03 , 0.03	0	0 , —	0.03 , 0.03	0	0 , —	0.03 , 0.03	0	0 , —	0.03 , 0.03	0	0 , —	0.03 , 0.03	-0.01	-0.01 , —	0.03 , 0.03
ω_{13}	-3.1	-3.07	0.03 , -0.01	1.03 , 1.14	-3.11	-0.01 , 0	1.02 , 1.12	-3.08	0.02 , -0.01	1.02 , 1.15	-3.09	0.01 , 0	1.03 , 1.14	-3.06	0.04 , -0.01	1.01 , 1.12	-3.04	0.06 , -0.02	1.01 , 1.05
ω_{23}	1	1	0 , 0	0.21 , 0.21	1	0 , 0	0.21 , 0.2	1.01	0.01 , 0.01	0.21 , 0.21	1	0 , 0	0.21 , 0.21	1.01	0.01 , 0.01	0.21 , 0.21	1	0 , 0	0.21 , 0.2
η_3 **	0	-0.01	-0.01 , —	0.04 , 0.05	0	0 , —	0.04 , 0.04	-0.01	-0.01 , —	0.04 , 0.05	0	0 , —	0.04 , 0.05	-0.01	-0.01 , —	0.04 , 0.04	-0.01	-0.01 , —	0.04 , 0.04
λ_1	1.7	1.73	0.03 , 0.02	0.16 , 0.16	1.73	0.03 , 0.02	0.16 , 0.16	1.73	0.03 , 0.02	0.16 , 0.16	1.72	0.02 , 0.01	0.16 , 0.16	1.74	0.04 , 0.02	0.16 , 0.16	1.72	0.02 , 0.01	0.16 , 0.16
λ_2	1.3	1.32	0.02 , 0.02	0.14 , 0.14	1.32	0.02 , 0.01	0.14 , 0.13	1.32	0.02 , 0.01	0.14 , 0.14	1.32	0.02 , 0.01	0.14 , 0.14	1.32	0.02 , 0.02	0.14 , 0.13	1.32	0.02 , 0.02	0.14 , 0.14
λ_3	1.2	1.25	0.05 , 0.04	0.18 , 0.17	1.25	0.05 , 0.04	0.18 , 0.16	1.25	0.05 , 0.04	0.18 , 0.16	1.25	0.05 , 0.04	0.18 , 0.17	1.26	0.06 , 0.05	0.18 , 0.17	1.25	0.05 , 0.04	0.18 , 0.17

* For models '*nor-nor-cr*', '*nor-t-mod2-cr*' and '*nor-t_mod3-cr*', true values for these parameters were considered as $\Sigma(1,1) = 11 * (6/(6-2)) = 16.5,\ \Sigma(2,2) = 2 * (6/(6-2)) = 3$.

** Since true value is 0 relative bias for this parameter is not defined.

*** This parameter belongs to model '*t-t-mod1-cr*' only. Since data were simulated under '*t-t-mod3-cr*' model and the truth is unknown, $\bar{B}_p$, $\bar{R}_p$, $\bar{S}_p$ and $sd(\hat{\Theta}_p)$ were not calculated.

**** For model '*nor-nor*', true value was considered as $\sigma = \sqrt{2.5^2 * (4/(4-2))} = 3.54$

4.3. Simulation Study to Assess Accuracy of Dynamic Predictions

A final simulation study to assess accuracy of dynamic predictions was performed. To account for the presence of both outliers in the longitudinal sub-model and the competing risks in the event sub-model, a '*t-t-mod3-cr'* competing risks JM structure was used to simulate the data, as the model with best fit to IDEAL PD training dataset adjusting for competing risks. The structure of the data generating model includes all baseline risk factors for death and competing risk events along with the linear mixed model for time-varying albumin, as per the '*t-t-mod3-cr'* model fitted to the IDEAL dataset with incident PD patients:

$$Y_{ij} = Y_i^*(t_{ij}) + Z_{ij} = \alpha_1 + \alpha_2 t_{ij} + b_{1i} + b_{2i} t_{ij} + \alpha_3 Gender + \alpha_4 BMI + \alpha_5 Stoke/Davies\ Score + \alpha_6 Calcium + \alpha_7 Creatinine + \alpha_8 Hemoglobin + Z_{ij} \quad (28)$$

$$h_{i1}(t) = \lambda_1 t^{\lambda_1 - 1} \exp(\omega_{11} + \omega_{21} Gender + \omega_{31} Age + \omega_{41} Stoke/Davies\ Score + \omega_{51} Initdose + + \omega_{61} Creatinine + \omega_{71} Urea + \omega_{91} Cholesterol + \eta_1 Y_i^*(t) \quad (29)$$

$$h_{i2}(t) = \lambda_2 t^{\lambda_2 - 1} \exp\big(\omega_{12} + \omega_{22} Age + \omega_{32} BMI + \omega_{42} Smoker + \omega_{52} Numperitepisodes + \eta_2 Y_i^*(t)\big) \quad (30)$$

$$h_{i3}(t) = \lambda_3 t^{\lambda_3 - 1} \exp\big(\omega_{13} + \omega_{23} Age + \omega_{33} Ethnicity + \omega_{43} Stoke/Davies\ Score + \omega_{53} Smoker + \eta_3 Y_i^*(t)\big), \quad (31)$$

where Eq. (28) represents the specification of the linear mixed sub-model, Eq. (29) corresponds to the survival sum-model and Eq. (30) and Eq. (31) describe the PH hazard models for the competing events of transfer to HD and KTx, respectively. Above, all variables are taken at baseline as in the actual models fitted to the IDEAL dataset. True parameter values used in the simulation were chosen as the posterior means of the MCMC samples that were estimated under model '*t-t-mod3-cr*' with IDEAL PD training dataset.

The simulation set-up follows a procedure described in a recent study on robust joint models[16] (however, no competing risks were considered in it). Repeated albumin measurements and competing events were simulated for a total of 7 patients selected at random from the internal validation dataset from the IDEAL trial (4 of them experienced death, 2 of them were transferred to HD and 1 was transplanted). Thus, these patients were not part of the training dataset used to fit the models and the value of their baseline covariates were kept fixed in the simulation study. Similarly to IDEAL trial design, albumin was simulated assuming data collection pattern with records every 3 months during the first 3 years of follow-up, and then every 6 months until year 6 of follow up. 20 replications were simulated for each of the patients under the '*t-t-mod3-cr*' model data generating structure. Then, the replicate datasets we combined to form a single dataset, consisting of a total of 140 patients. As suggested in prior literature, the reason for replications was to obtain data from the desired distribution, whereas the idea behind using 7 subjects was to keep heterogeneity due to individuals low[16]. As in the previous simulations, the inverse transform sampling method was used to find event times under each competing risk assuming Weibull cause-specific hazards along with a Weibull distribution for right censoring (shape = 6, scale = 7). Table S25 provides frequencies of subjects who were at risk, subjects who passed away or had a competing event, and who were censored within the forecast horizons at the selected landmark times of years 1, 1.5 and 2 following start of peritoneal dialysis and the two forecast horizons, 6 months and 1 year, respectively.

Table S25. Patients at risk at selected landmark times and forecast horizons in simulation dataset (N=140). The table shows number of patients (and percentages) who were at risk, died, were transferred to HD (TrHD), had kidney transplantation (KTx) or censored at the respective prediction horizons and landmark times (LMT).

	At risk	Death	TrHD	KTx	Censored
**Forecast horizon 6 months**					
LMT 1 year	115	5 (4.3%)	2 (1.7%)	2 (1.7%)	0 (0%)
LMT 1.5 years	106	7 (6.6%)	5 (4.7%)	2 (1.9%)	0 (0%)
LMT 2 years	92	6 (6.5%)	3 (3.3%)	2 (2.2%)	0 (0%)
**Forecast horizon 1 year**					
LMT 1 year	115	12 (10.4%)	7 (6.1%)	4 (3.5%)	0 (0%)
LMT 1.5 years	106	13 (12.3%)	8 (7.5%)	4 (3.8%)	0 (0%)
LMT 2 years	92	15 (16.3%)	7 (7.6%)	3 (3.3%)	0 (0%)

Similarly to prediction assessment of models using IDEAL internal validation dataset, the results for both time-varying Area Under the Curve (AUC) and Brier Score (BS) were calculated at landmark times $s = 1, 1.5$ and 2 years after PD start for two prediction horizons: 6 months and 1 year. All 12 JM (6 without competing risks and 6 with competing risks from Table 1 in main text) as fitted to IDEAL PD training dataset were used to compare the accuracy of the generated predictions with the simulated prediction dataset. Furthermore, the two Cox models fitted to the IDEAL PD training dataset for sensitivity analysis, one with baseline albumin ('*Cox-1*' as defined in main text) and the other being the extended Cox model with albumin as a time-varying covariate ('*Cox-2*' as defined in main text), which both share the same baseline risk factors as the survival sub-model in Eq. (29) above, were used to generate dynamic predictions and their results were compared with the results from the 12 joint models.

Results on AUC from Figure S22 largely support conclusions from Figure 4 from main text. It can be seen that, for the six models which ignore competing risks (black triangles) misspecifications in the longitudinal albumin sub-model do not have such a detrimental impact on prediction accuracy. However, accounting for competing risks provides an increase in prediction discrimination, across various albumin sub-model structures in different landmark and forecast horizons in Figure S22. Of note, the panels of Figure S22 are in good agreement with the corresponding results in IDEAL trial PD testing dataset (Figure 4 in main text), which once again confirms the suitability of model '*t-t-mod3-cr*' to model IDEAL trial PD data and validates the simulation set-up used to investigate prediction accuracy. All JM outperform dramatically the Cox model with baseline albumin only ('*Cox-1*' as defined in main text). In isolated cases, the Cox model with a time-varying albumin ('*Cox-2*' as defined in main text) competes well with JM, e.g. at landmark 1.5 years in both prediction horizons (middle panels of left and right columns of Figure S22). Thus, JM outperform simpler Cox models in terms of prognostic discriminatory potential (higher AUC values). Overall, JM demonstrate better calibration of model predictions depicted by lower BS values on Figure S23 than considered Cox models, with an advantage for JM with competing risks in some landmark and time horizon combinations.

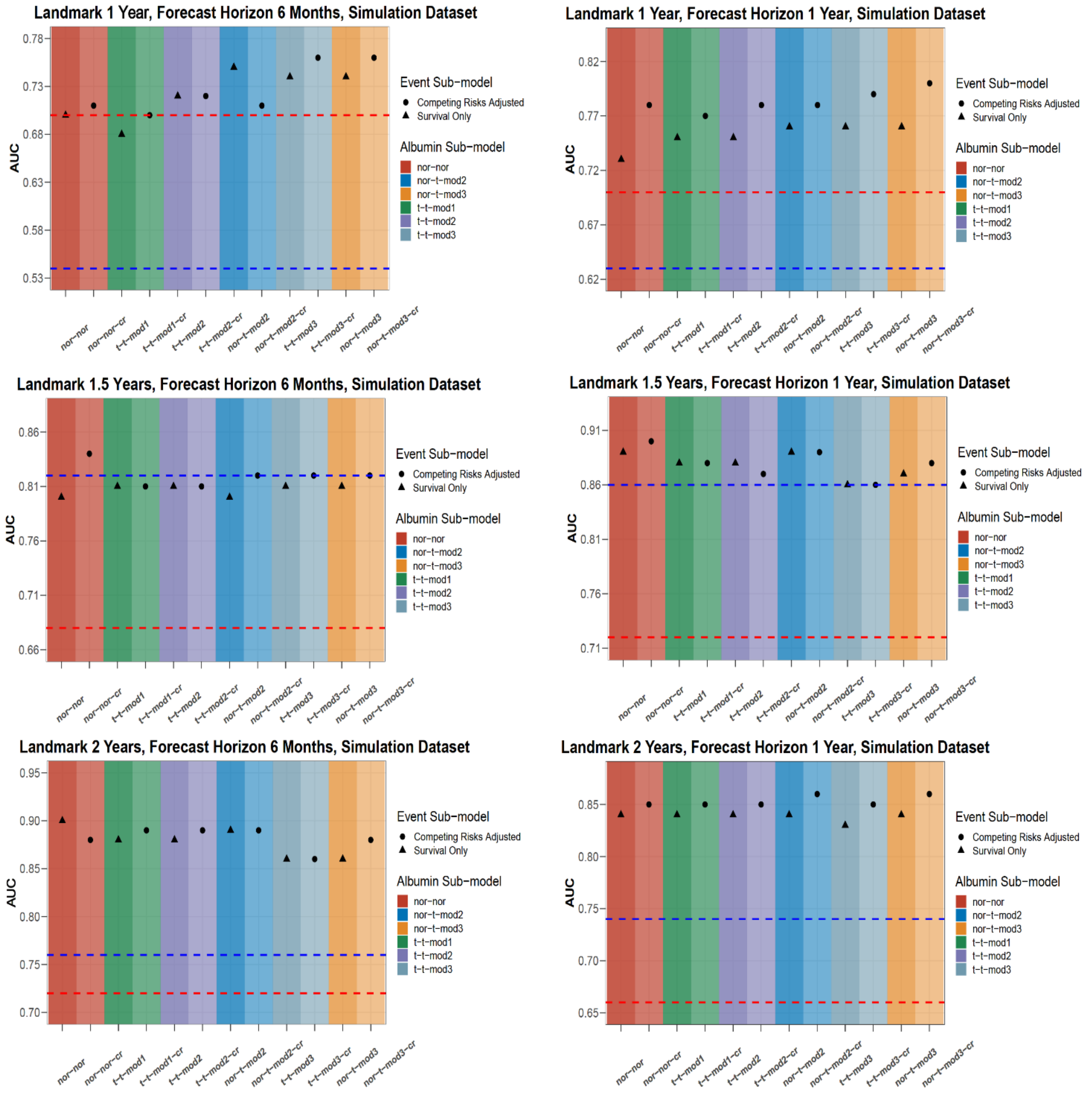

Figure S22. Area under the curve (AUC) of dynamic predictions for all landmark times and two prediction horizons from simulation study. Left column displays results for forecast horizons of 6 months, right column displays results for forecast horizons of 1 year. Red dashed horizontal line indicates result from Cox PH model with baseline albumin value (‘*Cox-1*’ in text) and blue dashed line indicates result from extended Cox PH model with albumin as a time-varying covariate (‘*Cox-2’* in text).

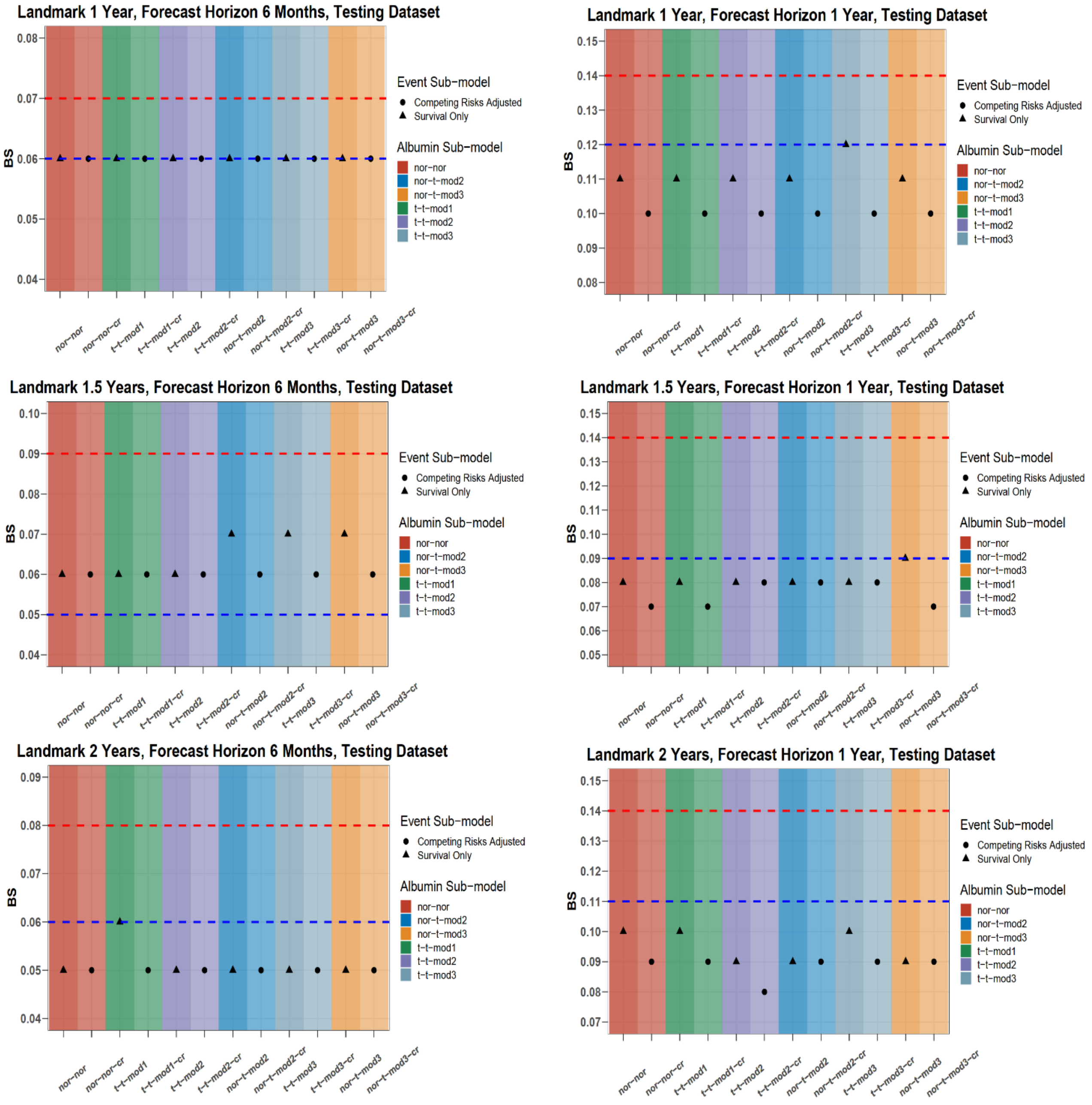

Figure S23. Brier score (BS) of dynamic predictions for all landmark times and two prediction horizons from simulation study. Left column displays results for forecast horizons of 6 months, right column displays results for forecast horizons of 1 year. Red dashed horizontal line indicates result from Cox PH model with baseline albumin value (‘*Cox-1*’ in text) and blue dashed line indicates result from extended Cox PH model with albumin as a time-varying covariate (‘*Cox-2*’ in text).

References

1 Zhudenkov, K. *et al.* A workflow for the joint modeling of longitudinal and event data in the development of therapeutics: Tools, statistical methods, and diagnostics. *CPT: Pharmacometrics & Systems Pharmacology* **11**, 425-437 (2022).

2 Ripley, B. *et al.* Package 'mass'. *Cran r* **538**, 113-120 (2013).

3 Faucett, C. L. & Thomas, D. C. Simultaneously modelling censored survival data and repeatedly measured covariates: a Gibbs sampling approach. *Statistics in medicine* **15**, 1663-1685 (1996).

4 Wulfsohn, M. S. & Tsiatis, A. A. A joint model for survival and longitudinal data measured with error. *Biometrics*, 330-339 (1997).

5 Rizopoulos, D. *Joint models for longitudinal and time-to-event data: With applications in R*. (CRC press, 2012).

6 McFetridge, L. M., Asar, Ö. & Wallin, J. Robust joint modelling of longitudinal and survival data: Incorporating a time-varying degrees-of-freedom parameter. *Biometrical Journal* **63**, 1587-1606 (2021).

7 Hsieh, F., Tseng, Y. K. & Wang, J. L. Joint modeling of survival and longitudinal data: likelihood approach revisited. *Biometrics* **62**, 1037-1043 (2006).

8 Brilleman, S. L. *Joint longitudinal and time-to-event models: development, implementation and applications in health research*, Unpublished doctoral dissertation, Monash University, (2018).

9 Prentice, R. L. Covariate measurement errors and parameter estimation in a failure time regression model. *Biometrika* **69**, 331-342 (1982).

10 van Boven, N. *et al.* Toward personalized risk assessment in patients with chronic heart failure: detailed temporal patterns of NT-proBNP, troponin T, and CRP in the Bio-SHiFT study. *American heart journal* **196**, 36-48 (2018).

11 Hickey, G. L., Philipson, P., Jorgensen, A. & Kolamunnage-Dona, R. Joint modelling of time-to-event and multivariate longitudinal outcomes: recent developments and issues. *BMC medical research methodology* **16**, 1-15 (2016).

12 Baghfalaki, T., Ganjali, M. & Berridge, D. Robust joint modeling of longitudinal measurements and time to event data using normal/independent distributions: a Bayesian approach. *Biometrical Journal* **55**, 844-865 (2013).

13 Baghfalaki, T., Ganjali, M. & Hashemi, R. Bayesian joint modeling of longitudinal measurements and time-to-event data using robust distributions. *Journal of biopharmaceutical statistics* **24**, 834-855 (2014).

14 Asar, Ö., Bolin, D., Diggle, P. J. & Wallin, J. Linear mixed effects models for non-Gaussian continuous repeated measurement data. *Journal of the Royal Statistical Society Series C: Applied Statistics* **69**, 1015-1065 (2020).

15 McCrink, L. *Outlier effects on robust joint modelling of longitudinal and survival data*, Doctoral Dissertation, Queen's University Belfast, (2014).

16 Asar, Ö., Fournier, M.-C. & Dantan, E. Dynamic predictions of kidney graft survival in the presence of longitudinal outliers. *Statistical Methods in Medical Research* **30**, 185-203 (2021).

17 Campbell, K. R. *et al.* Comparison of a time-varying covariate model and a joint model of time-to-event outcomes in the presence of measurement error and interval censoring: application to kidney transplantation. *BMC medical research methodology* **19**, 1-12 (2019).

18 Campbell, K. R., Martins, R., Davis, S. & Juarez-Colunga, E. Dynamic prediction based on variability of a longitudinal biomarker. *BMC Medical Research Methodology* **21**, 104 (2021).

19 Juárez, M. A. & Steel, M. F. Model-based clustering of non-Gaussian panel data based on skew-t distributions. *Journal of Business & Economic Statistics* **28**, 52-66 (2010).

20 Lau, B., Cole, S. R. & Gange, S. J. Competing risk regression models for epidemiologic data. *American journal of epidemiology* **170**, 244-256 (2009).

21 Lavalley-Morelle, A., Timsit, J. F., Mentré, F., Mullaert, J. & network, O. Joint modeling under competing risks: Application to survival prediction in patients admitted in Intensive Care Unit for sepsis with daily Sequential Organ Failure Assessment score assessments. *CPT: Pharmacometrics & Systems Pharmacology* **11**, 1472-1484 (2022).
22 Putter, H., Fiocco, M. & Geskus, R. B. Tutorial in biostatistics: competing risks and multi-state models. *Statistics in medicine* **26**, 2389-2430 (2007).
23 Andrinopoulou, E.-R., Rizopoulos, D., Takkenberg, J. J. & Lesaffre, E. Combined dynamic predictions using joint models of two longitudinal outcomes and competing risk data. *Statistical methods in medical research* **26**, 1787-1801 (2017).
24 Ferrer, L., Putter, H. & Proust-Lima, C. Individual dynamic predictions using landmarking and joint modelling: validation of estimators and robustness assessment. *Statistical methods in medical research* **28**, 3649-3666 (2019).
25 R Core Team, R. R: A language and environment for statistical computing. (2013).
26 Brooks, S., Gelman, A., Jones, G. & Meng, X.-L. *Handbook of markov chain monte carlo*. (CRC press, 2011).
27 Hoffman, M. D. & Gelman, A. The No-U-Turn sampler: adaptively setting path lengths in Hamiltonian Monte Carlo. *J. Mach. Learn. Res.* **15**, 1593-1623 (2014).
28 Gabry, J. Č., Rok. *CmdStanR: R Interface to CmdStan*, <https://mc-stan.org/cmdstanr> (2021).
29 Team, S. D. *RStan: the R interface to Stan.*, <https://mc-stan.org/. > (2023).
30 Khan, S. A. & Basharat, N. Accelerated failure time models for recurrent event data analysis and joint modeling. *Computational Statistics* **37**, 1569-1597 (2022).
31 Watanabe, S. & Opper, M. Asymptotic equivalence of Bayes cross validation and widely applicable information criterion in singular learning theory. *Journal of machine learning research* **11** (2010).
32 Martins, R. A flexible link for joint modelling longitudinal and survival data accounting for individual longitudinal heterogeneity. *Statistical Methods & Applications* **31**, 41-61 (2022).
33 Andrinopoulou, E.-R., Harhay, M. O., Ratcliffe, S. J. & Rizopoulos, D. Reflection on modern methods: dynamic prediction using joint models of longitudinal and time-to-event data. *International Journal of Epidemiology* **50**, 1731-1743 (2021).
34 Rizopoulos, D. JM: An R package for the joint modelling of longitudinal and time-to-event data. *Journal of statistical software* **35**, 1-33 (2010).
35 Furgal, A. *Bayesian Models for Joint Longitudinal and Multi-State Survival Data*, Doctoral Dissertation, University of Michigan, (2021).
36 Pinheiro, J. C., Liu, C. & Wu, Y. N. Efficient algorithms for robust estimation in linear mixed-effects models using the multivariate t distribution. *Journal of Computational and Graphical Statistics* **10**, 249-276 (2001).
37 Foley, R. N. *et al.* Mode of dialysis therapy and mortality in end-stage renal disease. *Journal of the American Society of Nephrology* **9**, 267-276 (1998).
38 Sameiro-Faria, M. d. *et al.* Risk factors for mortality in hemodialysis patients: two-year follow-up study. *Disease markers* **35**, 791-798 (2013).
39 Chang, S. H., Russ, G. R., Chadban, S. J., Campbell, S. B. & McDonald, S. P. Trends in kidney transplantation in Australia and New Zealand, 1993–2004. *Transplantation* **84**, 611-618 (2007).
40 Crowther, M. J. & Lambert, P. C. Simulating biologically plausible complex survival data. *Statistics in medicine* **32**, 4118-4134 (2013).
